# Evaluating the digital dispensation of HIV self-testing (HIVST) among men who have sex with men (MSM) in Pakistan: Seeing through the Consolidated Framework for Implementation Research (CFIR) lens

**DOI:** 10.1371/journal.pgph.0005994

**Published:** 2026-09-11

**Authors:** Usman Ali, Muhammad Safdar Kamal Pasha, Ramesh Kumar, Rab Nawaz Samo, Raza Haider Tirmizi, Siddique Wali

**Affiliations:** 1 Research Consultant, Dostana, Lahore, Pakistan; 2 PhD Scholar, Health Services Academy, Lahore, Pakistan; 3 Professor of Public Health, Health Services Academy, Lahore, Pakistan; 4 Program Officer, HIV, United Nations Development Program, Lahore, Pakistan; 5 Project Director, Dostana, Lahore, Pakistan; 6 Project Director, Humraz, Karachi, Pakistan; University of Cape Town, SOUTH AFRICA

## Abstract

HIV self-testing (HIVST) has recently been introduced through a digital platform for men who have sex with men (MSM) in Pakistan, where they can order and receive OraQuick HIVST via mail. The current study aimed to evaluate the digital dispensation of HIVST to the MSM community in Pakistan. Semi-structured interviews with MSM who availed HIVST services were conducted through in-depth interviews (IDIs) and focus group discussions (FGDs). Participants were recruited from HIV community-based organizations (CBOs) in Karachi and Lahore. The interviews were coded, and thematic analysis was performed using NVIVO 14.0. Twelve MSM were interviewed in the IDIs, and two FGDs were conducted with a total of 12 MSM. Key emergent themes highlighted that digital dispensation reduces HIV testing barriers and allows peer diffusion of testing services. MSMs can order HIVST digitally to dispense at the place of their choice. This allows them to bypass perceived stigma faced during in-person testing, including in the CBOs. However, MSM still fear stigma at home where HIVST might potentially be delivered. Participants noted difficulty in navigating the digital platform and a lack of comprehensive services including, condoms, lubricants, and counselling. The participants emphasized the importance of demand generation through social media and dating applications. Provision of a comprehensive service delivery package, digital demand generation through social media, and dating applications is recommended for strengthening digital HIVST services.

## Introduction

Men who have sex with men (MSM) include gay and bisexual men, male sex workers, men who may not identify with these categories but engage in sexual activity with other men, and transgender women [1]. MSM are one of the five key populations at risk for HIV infection [2]. Globally, 7.6% of MSM live with HIV, which is ten times the global prevalence of HIV in the general population [2]. MSM are stigmatized and discriminated against in various jurisdictions. Around 63 countries have some law that prohibits same-sex relationships, with 12 countries proposing capital punishment for same-sex relationships [2,3]. Globally, 40.5% of MSM have reported facing stigma in the last 12 months [4].

Pakistan is one of the 12 countries that proposes life imprisonment for same-sex relationships under Section 377 of the Pakistan Penal Code [5]. Criminalization is one aspect of manifested stigma against MSM. A recent study found that 10% of MSM living with HIV reported exclusion from family because of their MSM identity, 3% reported being blackmailed, and 13% reported being physically harassed [5]. Thus, MSM face layered stigma stemming from religion, culture, social values, and the legal system. At the same time, MSM are the largest key population in the country, with a population estimate of around 1 million; around 5.4% of these are HIV positive. This is in contrast to a 0.1% prevalence in the general population. Thus, for every four people living with HIV in the country, one is MSM [6]. This highlights the health inequalities MSM face in Pakistan [1].

Despite being at immense risk of HIV in the country, the coverage of HIV services for MSM is low, partly because of the hidden nature of the community. According to the Pakistan AIDS Strategy, only 9% of MSM had access to HIV prevention, and 6% had access to HIV testing in 2019 [6]. This was in contrast to the United Nations Goal of 90-90-90 by 2020, which stated that 90% of people know their HIV status; that is, they have been tested and counseled on their HIV status [7].

For a decade, HIV services had been solely provided by venue-based HIV testing either within the community-based organizations (CBOs) or through outreach at hotspots [8]. However, with increased digitalization, MSMs are increasingly using dating applications for partner cruising. In the Pre-exposure Prophylaxis (PrEP) feasibility study conducted in 2020, MSM and transgender people reported that 65.7% used dating applications such as Grindr or Tinder. In addition, TikTok, Instagram, and Facebook were used by 34.7% of the participants for client cruising, while hotspot client cruising was also similar [9]. Thus, hotspot-based HIV service provision may pose serious limitations in reaching the MSM community. In addition, stigma and discrimination may deter MSM from engaging in venue-based testing services. HIV self-testing is a validated method of screening for HIV. People can self-sample and self-screen for HIVST with minimal guidance from a healthcare provider [10]. Leveraging the potential of digital dispensation and HIVST, a digital HIV prevention and HIVST dispensation program is piloted in two major cities of Pakistan: Lahore and Karachi. The intervention was rolled out to improve testing coverage for MSM in the country. However, the intervention has not been rigorously evaluated. In the current study, we aim to qualitatively evaluate the intervention and identify gaps with recommendations for intervention improvement.

## Materials and methods

This qualitative exploratory study was conducted from May 2025 to July 2025 in Lahore and Karachi.

### Ethics statement

Ethical approval for this study was obtained from the Health Services Academy, Pakistan(No.00017/HSA/PhD-2023. Dated 08 January 2025) in accordance with the Declaration of Helsinki. Written informed consent was obtained before data collection from all study participants.

### Description of the digital dispensation HIVST model

Two digital platforms were developed and linked: one providing information on HIV prevention, treatment, and facilities (Sehat Dost), and the other for ordering HIVST kits (QuickRes). The interface of Sehat Dost (www.sehatdost.com) has seven key modules. The last module facilitates digital HIVST dispensation. Once a user clicks on HIVST dispensation, it lands on a webpage of QuickRes (www.quickres.org). QuickRes is a global platform used to make appointments and reservations for HIV services [11]. MSM can place an order for oral fluid-based HIVST on QuickRes. The order is placed, and the venue and time for collecting HIVST are selected.The CBO staff provides HIVST kits through a digital service or staff. Reporting of HIVST is not necessary to the CBO staff.

To generate demand for HIV services, advertisements are made in CBO drop-in centers (DIC) as well as messaging on WhatsApp, which includes a package of two videos, one focusing on how to perform HIVST and the other on how to use digital platforms. The demand generation faces significant challenges, as CBOs have to work in relative secrecy given the legal and social atmosphere of the country.

### Participants

The study was conducted at two CBOs, Dostana (Lahore) and Humraz (Karachi). People above 18 years of age, male, identified as men who have sex with men, who have received both in-person and digital HIV testing services, were identified and recruited by the outreach workers of two CBOs. CBO workers and those who had been diagnosed with HIV were excluded from the study. CBO workers were excluded even if they were MSM, as they have high health literacy and are providers of the same service that was evaluated, while people already living with HIV do not require testing services. Purposive sampling was conducted.

### Interviews and the data collection process

Once eligible participants were recruited, they were given the option of being interviewed either individually (in-depth interview) or in a focus group discussion (FGD). The same eligibility criteria and interview guide were used for the in-depth interviews and FGDs. Semi-structured interviews were conducted face-to-face by a lead investigator using an interview guide. Interviews were conducted on CBO premises in a separate room, ensuring confidentiality. Interviews lasted from twenty-five minutes for IDIs to over an hour for FGDs. Remuneration was not provided to the participants. Participants were asked about their experience with digital platforms for HIVST, how it compares with in-person HIV testing at the CBO, perceived barriers, and benefits of digital HIVST services.

All interviews were conducted in Urdu, the national language of the country. Audio from interviews was recorded on a personal device. This was followed by manual transcription and English translation. Data collection and preliminary analysis were conducted concurrently. Saturation was considered when no new codes or concepts emerged from successive interviews. Saturation was reached after 12 interviews, after which 2 additional interviews were conducted to confirm the stability of the findings.

To ensure confidentiality, the data were anonymized by assigning codes to individual participants, such as MSM1 and MSM2. Only the interview personnel had access to the anonymous data.

### Data analysis

The transcripts were imported into NVIVO version 14.0 [12]. Analysis was driven by an inductive thematic analysis followed by post-hoc interpretation using Consolidated Framework for Implementation Research (CFIR) as an analytic lens. CFIR is a key framework used in implementation science to evaluate complex interventions, including public health interventions [13–15].

The data transcript was available to two independent researchers. After becoming familiar with the data, the researchers independently conducted description- and interpretation-focused coding. In the description-focused coding technique, the researcher assigns a code that captures the participant's experience without inference. In interpretation-focused coding, the researcher assigns codes to capture the underlying processes of the experience. Any discrepancy in coding was settled by mutual consensus and deliberation guided by a third researcher. After coding had been completed, codes were sorted into clusters using an individual-based sorting strategy. Codes were sorted into clusters based on similarities and differences. Final themes were arrived at to answer the research questions. For this study, the key research questions were: 1) Is the current digital HIVST an acceptable and useful intervention for the MSM community? 2) What are the opportunities to adapt digital HIVST services to make them more effective for improving HIV testing among MSM?

In the final step, themes were interpreted onto CFIR domains and constructs. Where appropriate, themes were mapped on more than one CFIR domain to reflect their complexity and cross-cutting nature.

The whole process was iterative, conducted through deliberations between the research team. The detailed methodology of coding and sorting was adapted by Adu et al [16]. The results were reported using Consolidated Criteria for Reporting Qualitative Studies (COREQ) [17].

Throughout the research, reflexive discussions were undertaken to examine the impact of researchers’ backgrounds and experiences on research. The practice of independent coding by two researchers, followed by iterative sorting, helped bracket individual perspectives of the researchers and enhance methodological rigor. Findings were supported by verbatim quotes from the transcripts to ensure credibility and transparency.

### Reflexivity statement

The lead investigator has been working in HIV for nearly seven years, whereas MSKP has been working for nearly two decades. RHT has been involved in community research and various qualitative projects, whereas RK has worked at the national level in HIV response with various public health agencies. The lead investigator identifies as a gay man and is a community health rights advocate, a psychiatrist, and a public health professional. The lead investigator advocates for readily available services free of stigma and discrimination for the MSM community. RK has worked in public health for over two decades, providing an overview of the data coding and analysis processes.

## Results

Twelve IDIs were conducted in total. All participants had at least intermediate education (12 years of education). All except one were employed. All participants had their first HIVST (index) in a drop-in center (DIC). The summary characteristics of the participants interviewed through the IDI are given in the Table 1 below.

**Table 1:** Characteristics of in-depth interviews, n = 12.

Serial no.	Participant ID	Age in years	Education	Occupation	Income in PKR	Index HIVST
1.	MSM1	25	Bachelors	Lawyer	70000	DIC
2.	MSM2	23	Bachelors	Veterinary doctor	60,000	DIC
3.	MSM3	21	Bachelors	Freelance advertiser	100,000	DIC
4.	MSM4	21	Bachelors	Unemployed	0	DIC
5.	MSM5	22	Intermediate	Fashion designer	40,000	DIC
6.	MSM6	28	Bachelors	Paramedic	25000	DIC
7.	MSM7	23	Bachelors	Part-time employee	25,000	DIC
8.	MSM8	47	Masters	Artist	100,000	DIC
9.	MSM9	24	Bachelors	Advertising agency	140,000	DIC
10.	MSM10	25	Bachelors	Office boy	45,000	DIC
11.	MSM11	27	Masters	Display designer	100,000	DIC
12.	MSM12	28	Bachelors	Sex work/ office work	45,000	DIC

In addition, two focus group discussions were conducted with 12 MSM. The mean age of the participants was 25.85 years. All participants had at least matriculated. Three of the 12 participants were unemployed. The mean income of participants was 24,500 PKR. The summary characteristics of the participants interviewed through FGD are presented in the Table 2 below.

**Table 2:** Characteristics of participants interviewed in the focus group discussion (FGD), n = 12.

Serial no.	Characteristics	Frequency (Percentage)
1.	Mean age of participants in years	25.85
2.	Education	
	No education	0 (0%)
	Under Matric	0 (0%)
	Matric to Intermediate	9 (75%)
	Graduation and above	3 (25%)
3.	Employment status	
	Employed	9 (75%)
	Unemployed	3 (25%)
4.	Mean income of participants in Pakistani Rupees	24,500

Based on thematic analysis of the qualitative interviews, three themes and eleven subthemes emerged. The Table 3 below presents the thematic analysis mapping on CFIR domains and constructs.

**Table 3:** Mapping of themes and sub-themes on CFIR domains and constructs.

Theme	Subtheme	CFIR domain	CFIR constructs
Digital dispensation reduces HIV testing barriers and allows peer diffusion to MSM not reached by CBOs	Digital HIVST dispensation provides better privacy and control over sexuality and HIV status disclosure	Innovation, Individuals	Innovation relative advantage, Individual recipient's capability
	HIVST reduces cost and time barriers to HIV testing	Innovation	Innovation relative advantage
	Peer-driven diffusion of HIV testing to MSM not accessible by the CBOs	Innovation, outer setting, Individuals	Innovation adaptability, Innovation recipient needs, and resources
	Digital guidance and a simple interface facilitate platform usability	Innovation, outer setting, Individuals	Innovation adaptability, Communications, Implementation facilitators
Digital platform users trade off between stigma at CBOs vs at home for HIV testing	Fear of enacted stigma while dispensing HIVST at home	Innovation, Outer setting	Local attitudes, Local conditions, Innovation relative advantage
	Lack of privacy at home	Innovation, Outer setting	Local attitudes, Innovation relative advantage
HIVST digital platform needs system maturation, demand generation, and integration in broader HIV services	Users face problems related to the design and structure of the digital platform	Innovation, Inner setting	Innovation design, Information technology infrastructure, Compatibility
	Users face problems related to the content of the platform	Innovation	Innovation design
	Users request the provision of comprehensive services through digital order	Individual	Innovation recipient need
	Using diverse demand generation through in-person interactions and digital means	Outer setting, Inner setting	Communications, Partnership, and connections

### Theme 1. Digital dispensation reduces HIV testing barriers and allows peer diffusion to MSM not reached by CBOs

Based on participant interviews, it was highlighted that the intervention had been favored positively by the users at individual levels, as well as having an impact at the community level. The participants preferred HIVST dispensation using a digital platform as it allows them to keep their HIV status and sexuality confidential. Having a positive experience with digital HIVST dispensation allowed them to overcome time and cost barriers to CBO-based HIV testing. The participants agreed on user-friendly guidance to use the digital dispensation system. Given their positive experience, they engaged their peers and included them in the HIV testing network, allowing the intervention to reach MSM not reached by CBOs directly, through secondary distribution.

### Subtheme 1.1. Digital HIVST dispensation provides better privacy and control over sexuality and HIV status disclosure

The participants compared digital HIVST dispensation with HIV testing in CBOs. The participants reported greater control, as they can now choose not to disclose their sexuality or HIV status to anyone. The perceived stigma from the community within CBOs is seen as a barrier. Other community members in CBOs may speculate about HIV test results based on behavior, while HIVST at home through digital dispensation bypasses this barrier, creating a favorable perception of digital HIVST compared to testing in CBOs. A participant reported that

*Because at home (where I get HIVST dispensed), no one else is around in that moment. But if you're taking the test here at the center (CBO), your facial expressions and how you react can give something away. If you get tense or scared (after getting the test), people around you (in the drop-in center) who are watching might notice. They’ll realize you're doing an HIV self-test and might assume the result is bad. MSM7, 23 years old, part-time employee, 14 years of education*

Though CBOs are a place for the MSM community, there are varying positions, such as paramedics or doctors who are not community members. The participant reported that through digital HIVST dispensation, they have more autonomy in terms of confidentiality.

*When we come here, we have to first go to the office and give our identity. Then we visit the doctor or the paramedic, and he conducts the test. But at home, we do it ourselves. We already know how. In Punjabi, we say “don’t open your mouth… meaning, just do it quietly and easily.FGD1*

Community members have varying levels of comfort in disclosing their sexuality and varying levels of self-acceptance or self-identity within the MSM spectrum. Coming to CBO may mean that they have to identify with a particular identity for which they are not ready. The digital intervention also empowers participants to seek HIV testing services while still processing their identities.

*Some people hesitate a lot while coming to the CBOs. They don't want to accept (their sexuality) for whatever reason they have. So, self-testing is very easy for them. They can test themselves at their place or anywhere. Some people have social anxiety; they don't want to face it. They don't know what will happen if they come here. MSM4, 21 years old, graduate, unemployed*

These findings suggest that digital HIVST dispensation empowers the participants in terms of privacy and confidentiality, thus making it more acceptable.

### Subtheme 1.2. Digital HIVST reduces cost and time barriers to HIV testing

Even though HIV testing is free at the CBO, there are time and travel-related costs for availing of on-site HIV testing by coming to the CBO. Digital HIVST dispensation reduces these barriers; thus, participants favor it compared to CBO-based HIV testing. One participant during the FGD stated that

*I think it has a lot of benefits, because sometimes you don’t have money to go to the CBO. They provide it free of cost, including delivery, which is very convenient. You get it for free. Many friends are busy during the day and don’t have time. FGD 1*

Another participant from the second FGD stated

*Yes, I had done the test before. But because of my busy schedule with university and classes, it was a bit difficult to come here. So doing it at home was much easier for me. I ordered it through a link from a digital platform. …. I received the kit, and I did the test myself. I rubbed the swab on my gums and then dipped it, just like the instructions said. FGD2*

These accounts emphasize how digital HIVST dispensation can be taken up by reducing financial and time-related barriers, without interfering with daily routine tasks.

### Subtheme 1.3. Peer-driven diffusion of HIV testing to MSM not accessible by the CBOs

Having a favorable experience with the digital HIVST dispensation, there is peer-driven diffusion of HIV services to other MSM. This creates a mechanism of secondary dispensation, allowing an increase in testing coverage and network expansion. This can have a communal benefit. Participants who were not healthcare staff of the CBO stated that they can now provide a link to services to other MSM who have not yet been reached by the CBOs, and would refrain from getting in touch with the CBO staff because of stigma, and maintain privacy. The potential of this digital intervention is recognized by one MSM, who stated that:

*If we limit it to CBOs, then it will not reach everyone. MSM are those who don't believe in themselves. It is a good thing to reach them. MSM4, 21 years old, graduate, unemployed*

Another participant recalled his experience of getting his friends tested through this service

*I feel that the test done here is different from the one done at home…. When two of my friends visited me, I got their tests done at home. MSM5, 22 years old, fashion designer by profession, with 12 years of education*

Another participant recalled a similar experience

*I found out through an XX worker (at the CBO) that you can even get it (HIVST) delivered to your home. Then I passed that on to my friends. I guided them too. I explained how to use the kit and how the process works. I showed them everything step by step. MSM6, 26 years old, paramedic by profession, 14 years of education*

The accounts validate the diffusion of intervention, ensuring network expansion with users playing the role of network expanders.

### Subtheme 1.4. Digital guidance and a simple interface facilitate platform usability

As mentioned above, the current structure of the intervention is the demand generation of HIVST by sharing instructions in the form of videos on how to operate and place an order on the digital platform, and a video on how to perform HIVST. These instructions, as well as the simple interface of the digital platform, are positively appraised by the community, enhancing confidence in the digital platform regarding its usability. During the FGD, the participant stated:

*It is very good. I had no idea about the website. But the video has made it easy for us. We have sent it to many friends. It is very good. It has a lot of clarity about how to use the website in Urdu, what questions are there, and how to do it. It is very easy. It has all the to-the-point information. FGD1*

Another participant highlighted that sending videos on WhatsApp made it easy to use the digital platform.

*If you want to book a kit digitally, then you have to go to QuickRes, right? Now, they also send videos. Like, XX (CBO worker), who sent me the video, he told me that if you want to tell someone further, then first send him a video so that he understands what it is, how to use it. So, sending a video is very beneficial. I also sent a link to him. There are some Facebook friends with whom I have never met, but there is a lot of talk on Facebook. MSM6, 26 years old, paramedic by profession, 14 years of education*

The participants acknowledged that the use of the Urdu language made navigation easier on a digital platform.

*No, it was very smooth. The website had a full Urdu language option. I didn’t even know it could be done digitally until a friend told me. FGD2*

However, these findings were not universal, and some gaps were highlighted by the participants, as given in Theme 3.

### Theme 2. Digital platform users trade off between stigma at CBOs vs at home for HIV testing

As mentioned above, the digital dispensation of HIVST offers an acceptable private and confidential testing service; it does not eliminate stigma. Stigma from testing in CBOs is replaced by stigma in testing at home. This is compounded by increased scrutiny and a lack of privacy at home. The latter risks enacted stigma, because HIV is linked to sexual transmission and homosexuals, by default. To overcome these barriers, MSM, instead of getting HIV dispensed at home, may choose dispensation in the vicinity to avoid any risk of disclosure. Thus, MSMs weigh the benefits of testing at home as mentioned in the theme above, with potential risks highlighted in this section, choosing a personally suited strategy. However, digital HIVST dispensation provides them with an additional testing option to choose from in HIV services.

### Subtheme 2.1. Fear of enacted stigma while dispensing HIVST at home

The participants reported not being out to family members as MSM, and risk exclusion from the family due to discrimination. One participant reported.

*That fear is always there (the fear of people knowing that you are MSM)… if people get to know, the reputation you have made as a straight man (by being in the closet) is tarnished. Your family prestige is at stake (as MSM is seen as a sin, abomination,. MSM8, 47 years old, artist by profession, 16 years of education*

To navigate in this fearful environment, they choose to get HIVST dispensed other than in their homes, but in the vicinity

*Many people do not give the address of their house, but give the nearby location of their house. For example, if there is a shop or a famous thing, if there is a shop a little ahead of their house, the rider goes there and calls them. FGD 1*

Another participant in the FGD said that

*The first thing that comes to mind is, where should I have it delivered? Because I can’t have it sent to my home. So I try to make sure it’s delivered somewhere far from home. Apart from that, I worry about how it will be. It's difficult for a newbie, but for a guide, it's not an issue. FGD2*

For this reason, some MSMs prefer CBO testing, preferring a supportive environment over inadvertent disclosure of sexuality at home. One participant stated that

*Here, the environment is friendly. It feels easier and more comfortable. At home, the family doesn’t know about this. You have to keep the testing private from them. Naturally, we can’t tell them. Some families are friendly, but in some households, you just can’t share things like this. I don’t think anyone would openly share something like this with their family. The good thing here is that there are friends around at the center, and the environment is friendly like home, but better. Still, if you think about it, going to and from the center takes time. At home, you can just take the test. MSM2, 23 years old, Veterinary doctor by profession*

These fears may deter participants from getting HIVST dispensed at home, and instead, they choose a nearby place or prefer HIV testing in the CBO.

### Subtheme 2.2. Lack of privacy at home

Linked closely with the fear of enacted stigma is the lack of privacy at home. This lack of privacy serves as the mechanism for the fear. The participants reported that hiding HIV testing kits at home is not possible, due to the lack of privacy and questioning in family settings. A participant stated that

*I don't think anyone can hide it and keep it at home. If he uses a kit, they (family) will check it. He should not keep it in front of his family and face their questions. MSM2, 23 years old, Veterinary doctor by profession*

When probed if a change of packaging without mention of HIV testing kits would solve this lack of privacy, a participant responded;

*No matter what kind of packaging you use, if someone in the family even has the slightest doubt, they’ll open it. Either that, or your reputation at home needs to be so solid that your privacy is respected. MSM8, 47 years old, artist by profession, 16 years of education*

The lack of privacy is a concern because of the association with HIV-stigmatized communities. A participant reported that he could justify HIV kits at home because everyone is at risk, and it is not only contracted through sexual means; however, this participant did not challenge the lack of privacy at home either.

*Even if you get it delivered to your home, there’s no issue because this is common. It’s not a gay disease or anything. It can happen randomly, even if you just go to a barber; their instruments can also be a source. This is a disease that can be contracted in many ways. So family members don’t need to find out about it just because it’s ordered at home. MSM9, 24 years old, working in an advertising agency, 14 years of education*

The above excerpts from the interview focus on the prevalent generalized stigma against the HIV and MSM community in the households, triggering fear and posing barriers to HIV testing.

### Theme 3. HIVST digital platform needs system maturation, demand generation, and integration in broader HIV services

Although the intervention was valued by the participants, several technical limitations were also highlighted, which may impact sustainability. In addition, the participants called for system integration with comprehensive HIV services. The finding reflects community readiness and the realization of a need for associated services. Participants also highlighted that for maximum impact, diverse digital demand generation is needed, leveraging social media and dating applications. However, digital interventions should not replace human interaction, both in demand generation at the community level but also individual counselling for service users.

### Subtheme 3.1. Users face problems related to the design and structure of the digital platform

The current structure of having two platforms, one for HIV services and one for HIVST dispensation, was seen as complex by participants. The participants reported inconsistent linkages between the two, which make navigation difficult. Thus, optimal operation of the platforms requires a good level of digital literacy and comfort. However, given the literacy levels in MSM, who are marginalized, this is not optimal. This may limit the use of the platform to certain MSM rather than the MSM community as a whole. One participant stated that the platform is not designed for average MSM.

*This platform isn’t really for the average person; only someone who’s a graduate or at least well-educated can manage to place an order. An uneducated or less literate person wouldn’t be able to do it easily. Only graduates can do this. But there should also be an option to call. You should call and receive a call from the representative. You should tell him all the details, your address, and it should reach you. FGD2*

Since HIVST can be ordered from *Quickres*, but HIV prevention information is on the Sehat Dost platform, it may be cumbersome for some participants, as one participant stated:

*As far as I know, both things should be on the same platform….So, I will not need to visit other platforms. MSM6, 26 years old, paramedic by profession, 14 years of education*

Another participant highlighted that even among the two platforms, the linkage is inadequate

*No, there’s no option for Sehat Dost in QuickRes. The QuickRes option is available on Sehat Dost, but not vice versa. FGD2*

This lack of linkage between platforms causes people to miss content on the complementary platform.

*Some people know about it, and some don't. But it depends on the chain. As I know, if someone consults me, I will guide them properly. MSM6, 26 years old, paramedic by profession, 14 years of education*

Apart from having two platforms as part of the digital intervention, participants also reported some technical problems related to bookings. One participant stated the following:

*There are time slots shown at the beginning; you’re asked to select one for delivery. Different time slots appear, and you’re supposed to choose one. But at first, they don’t get selected. Then, when you move to the next page, it shows that the slots are available, but on the next screen, they aren’t. The system just assigns a random time on its own sometimes at a time when you’re not available or there is any problem, … MSM1, 25 years old, lawyer by profession*

These reflections emphasize the need for system maturation, comprehensive single platforms designed for those with low digital literacy for maximum utilization.

### Subtheme 3.2. Users face problems related to the content of the platform

The current language and data collection at the digital platform reflect use of technical jargon related to sexual identities. This creates confusion and mistrust among users. In addition, extensive data collection creates fear of data security and privacy. The participants’ emphasis on these points reflects that the current content is not user-centered. Speaking about the technical language used on the platform, one participant stated that

*There are a few questions that confuse people, like MSW, MSM, or bisexual. When we explain that these terms are for the community and that there’s nothing to be afraid of and that their data is safe, then they’re more comfortable. Some people did ask me what MSW, MSM, or bisexual means. These are just written differently, and people often get confused. But it’s not a problem once it’s explained; they’re fine. Yes, people are a little afraid at first. FGD2*

Some participants also criticized the platform for asking for too much information before booking. The participants are concerned about digital privacy, as giving personal information on a digital platform has risks and triggers anxiety.

*I saw their questions; there were quite a lot, maybe 2–3 pages worth. They were very detailed. MSM5, 22 years old, fashion designer by profession, with 12 years of education*

Thus, the content needs to be centered around user-friendly language, which shall improve trust. In addition, the collection of personal information should be minimized to encourage service use.

### Subtheme 3.3. Users request the provision of comprehensive services through digital order

Participants have recommended the provision of comprehensive HIV services digitally, reflecting the community's readiness for uptake of digital services. Provision of these services should not be limited to HIVST but include condoms and lubricants as well as digital counselling for HIV test-takers.

One participant stated that

*Condoms or lubricants, I believe, should be made available. MSM 2*

Similarly, another participant emphasized the same idea that

*And apart from that, there should be an option for condoms as well. For example, if someone wants to use condoms but doesn’t feel comfortable asking for them openly, then they should be able to get them delivered discreetly to their home, too. That would also be very beneficial in terms of protection. MSM6, 26 years old, paramedic by profession, 14 years of education*

Other participants recommended HIV digital counselling. One participant highlighted this

*It is a digital service, and they are providing a kit, so counselling should also be done as the kits are provided. There can be digital counselling, and it can also be done via video call. Now, obviously, it is the age of digital. If there can be digital classes and digital meetings. So why can't counselling be done digitally? MSM 2*

These excerpts highlight that the digital intervention can have a scope of expansion to comprehensive services, given the community demand.

### Subtheme 3.4. Using diverse demand generation through in-person interactions and digital means

The participants recommended the need for a multipronged digital demand generation. These include outreach at dating applications, as well as social media. However, digital demand generation should not replace human interaction. In fact, interpersonal interaction can complement digital demand generation, ensuring uptake of services. One participant stated that

*We can't send it through just by sharing the link, as it is sensitive. You can only share it after a deep conversation. Only then will the next person understand it and be able to use it positively. You can also give advertisements on Grindr and other dating applications*. *FGD1*

While some people have stressed the use of dating applications for advertising, others have stated the use of social media applications for the demand generation of HIV services and self-testing.

*Not everyone uses Grindr. For example, I don’t use Grindr. Most people use Instagram and Facebook (for hookups and dates). Even within the gay community, not everyone is on Grindr. MSM7, 23 years old, part-time employee, 14 years of education*

One participant stated that there should be group sessions to complement digital messaging, and stated that people may take digital messaging for granted.

*There should be more awareness sessions in the CBOs, too. They should hold regular sessions so people can learn. Because most people don’t read everything on the app. They either skip through or just don’t bother going into full detail. FGD2*

These reflections from the participants highlight the need for multipronged and well-connected digital outreach and demand generation, leveraging social media and dating applications, with interpersonal interactions, individually or as a group, rather than just sharing a platform link.

## Discussion

### Framework for evaluating digital HIVST dispensation

The real-world effectiveness of interventions depends on several factors, including factors other than the characteristics of the intervention itself [14,15]Various theories, models, and frameworks have been proposed to ascertain the effectiveness of interventions [14,15]. One of the most influential frameworks is CFIR, proposed by Damschroder et al. The framework includes five major domains for evaluating complex interventions: innovation, process, inner setting, outer setting, and individual characteristics of users, as well as facilitators and implementers [13]. Our evaluation highlights that each of these domains influences user experiences and contributes to intervention uptake.

### Acceptability of HIVST intervention

Zhu et al. conducted a randomized controlled trial comparing HIVST delivery through digital dispensation and in-person dispensation. The study showed that the option of digital dispensation increased HIV testing two-fold [18]. In two separate RCTs that used Internet-based HIVST dispensation, it was noted that testing rates increased 1.47 to 1.77-fold [19]. However, there is significant heterogeneity observed in acceptance and preference for digital HIVST interventions. In a recent review, the acceptance of digital HIVST ranged from 64.55% to 99.0%. The preference for digital HIVST interventions ranged as low as 4.6% to 99.6% [20]. In a meta-analysis pooling results of digital HIVST interventions from low- and middle-income countries, HIV testing uptake was similar between digital and non-digital interventions [21].

The participants in our sample emphasize the convenience and resource efficiency of digital dispensation of HIVST, stating relative advantage over facility-based HIV testing. In a previous pilot intervention of HIVST dispensation through CBO workers, 96.6% of MSM reported HIVST convenient to use. In the study, 55.3% of MSM preferred outreach through social media [22]. The current evaluation highlights that HIVST itself and its digital dispensation are acceptable to the MSM community. However, there are several caveats to this acceptability and preference which are covered in sections below.

### Digital HIVST dispensation, stigma and mental health support

MSMs are marginalized in many Muslim-majority countries, with Pakistan being one of them [3]. This poses unique challenges in reaching out to the community with health and HIV interventions. Stigma is faced in family settings, legal frameworks, as well as internalized or perceived stigma within community settings [23]. In the current study, participants have described how stigma, even within community settings such as CBOs, serves as a barrier to HIV testing uptake. HIVST through digital means provides an additional testing option for the MSM community in that context. However, the stigma in CBO testing is replaced by fear of stigma in home settings, an interaction of inner and outer settings. Thus, digital dispensation of HIVST is influenced by stigma but in different contexts.

The fear of enacted stigma is not unfounded, as MSM and people living with HIV (PLHIV) are highly stigmatized in the context of Pakistan. In the National HIV Stigma Index 2.0, 10% of MSM living with HIV reported that they were excluded from family settings in the last twelve months because of their identity as MSM [5]. Around 21.53% of MSM PLHIV also reported that they felt they were gossiped about and discriminated against by family in the last year because they are MSM. The stigma is not limited to same-sex prefrences or behaviors. PLHIV are also excluded and discriminated against in family settings [5]. Around 5% of PLHIV report that they are excluded from family events in the last twelve months. Due to this stigma, people refrain from sharing their HIV status with their family [5]. In the National Stigma Index 2.0, 26.6% of MSM PLHIV reported they feel uncomfortable sharing their HIV status with those close to them due to stigma. Testing in such a stigmatizing environment thus brings anxiety, as reported by the participants [5]. In a meta-synthesis of HIVST interventions among MSM in Asia, it was found that mental distress is in fact a barrier for HIVST uptake [10]. The participants from our study have, in fact, stressed the need for counselling services, though provided digitally for users of digital dispensation.

### Comprehensive service provision

According to the World Health Organization, comprehensive HIV prevention services include HIV testing services, condoms, PrEP, and the management of sexually transmitted infections [24]. The digital platform in our intervention provided HIV information and -care and HIVST kits. The participants, however, recommended comprehensive services that they can access at CBOs, such as condoms and lubricants. This reflects the participants’ needs and readiness for uptake of services. Provision of these services shall also require ‘relational connections’ with law enforcement agencies. Condoms are not positively seen in society, and dispensation using riders may precipitate harassment from police [25]. In Malaysia, another Muslim-majority context, researchers have evaluated a digital application that provided a comprehensive HIV prevention package, including HIVST and PrEP [26]. This study employed a pre-post design. Among 50 MSM using the application, 84% used HIVST, with 42% receiving dispensation more than once [26].

### Demand generation

In terms of demand generation, the participants in the current study emphasized digital means. This includes the use of social media applications, which may be used for various purposes but may include cruising clients or dates. These platforms include Facebook and Instagram. However, the participants also highlighted the use of digital dating applications exclusively by MSM, such as Grindr or Blued. While the profile and risk behaviors of MSM who use dating applications have not been studied in Pakistan, it is not known if their risk behaviors are higher than those of MSM who do not use dating applications. Researchers from the United States have shown that MSM using Grindr had higher rates of chlamydia or gonorrhea infection than those who did not use GrindrHowever, the cohort was more likely to initiate HIV PrEP than non-users [27]. This highlights the systematic differences among MSM who use dating applications. Thus, the digital dispensation of HIVST can be an effective method for engaging this community by linking Quickres with dating applications for demand generation. However, it should also be noted that dating applications are banned in Pakistan by the government. Despite being banned by the government, proxy web administrators use these applications [28].

### Digital platform integration

While information on these is available on the Sehat Dost platform, participants highlighted that the presence of a separate platform for HIVST dispensation (Quickres) may be cumbersome to use. Although the current model of intervention relies on digital dispensation and mailing of HIVST, participants stated that they shared the link to the digital dispensation platform with other community members. It should be noted that this method may be used to engage MSM who cannot be reached by conventional outreach by CBOs. These themes illustrate the need for innovation and adaptability. In addition, the users also highlighted provider-centered language and data capture that need to be addressed. These recommendations are important, given that innovation is inherently diffusing to late adopters, who may be less motivated to use the service [29]. Providing client-centered and easy usability of the platform will improve user satisfaction and increase use of the platform.

### Peer-driven intervention diffusion

Most of the study participants were young, educated, and employed. Previous research has shown that young and socioeconomically advantaged individuals are adopters of health technology. In addition, all participants in IDIs had index HIVST in DIC, followed by ordering HIVST on a digital platform. This highlights the increasing confidence of the MSM community in the HIVST intervention. According to the social network model of diffusion of innovation, opinion leaders help uptake of innovation or intervention [29]. Our study highlights that the MSM were first introduced to the use of HIVST in the DIC, followed by ordering through the digital platform. The study participants also highlighted that they recommended the digital HIVST intervention to their peers, who were previously out of network reach by the CBOs. Thus, the intervention was able to reach them through social networks. This can increase the testing coverage of HIV among MSM in Pakistan. The global targets for HIV by 2025 were that 95% of people living with HIV know their status [2]. In the last national HIV strategy 2021–2025, it was revealed that only 2% of MSM had been tested for HIV in the last 12 months [6]. According to the National AIDS Control Program (www.cmu.gov.pk), 84,421 PLHIV know their HIV status out of 330,000 infected. This reflects that only 25% of PLHIV in the country are aware of their HIV status. The use of digital HIVST intervention can thus offer support in increasing testing coverage for the MSM community in the country.

### Policy implications

There are several policy implications from our study. First, there are some navigation difficulties for the MSM on the digital dispensation platform. Second, there are two platforms, one for HIV information and the second for ordering HIVST. These platforms should be simplified, and only one comprehensive platform serving both these purposes should be rolled out. Furthermore, comprehensive HIV services should be provided. This includes HIVST but also STI medications, PrEP, and condom dispensation digitally.

Rolling out these services by digital platform shall require review of current regulations of dispensation of medicinal products digitally in the country. It should also require liaison with law enforcement agencies, as there have been reports of people harassed for carrying condoms [30]. Thus, if digital dispensation of condoms is made, there is a need for engagement with critical enablers such as medical regulators and law enforcement agencies.

Increasingly, MSM have moved from conventional hotspots to digital dating spaces; however, bans on these applications make it impossible for national HIV programs to introduce digital demand generation. HIV advocates should lobby with the Government of Pakistan to unban these applications and launch a digital demand generation campaign for effective reach to unreached MSM.

### Limitation of the study

The current study, however, has several limitations. First, it explored the qualitative experiences of the participants. The participants were interviewed by a researcher who was a former manager at the CBO; this may induce reporting and desirability bias, where participants only reported favorable themes. The participants were from two cities in Pakistan; thus, their experience may not be generalizable to all MSM residing in the country. Furthermore, we only used CFIR for post-hoc inductive analysis. CFIR is a comprehensive tool for appraisal and planning of intervention. Many of the CFIR domains are related to implementer, organizational, and donor perspectives. Our study, thus, uses CFIR as a lens for capturing user perspectives while not evaluating the intervention from the viewpoint of other stakeholders. Nonetheless, the study has several strengths allowing for greater understanding of how outer settings influence HIVST digital dispensation and how the innovation can be adapted for improved efficiency.

## Conclusion

Digital HIVST dispensation is acceptable. There is a community readiness to uptake a comprehensive package of HIV services digitally, in addition to HIVST. The intervention also holds promise of network expansion of services driven by peers to MSM not reached by the CBOs. However, participants noted difficulty in platform navigation. Thus, the current digital dispensation platform should be improved and should include comprehensive HIV services such as dispensation of condoms and STI medications.

There is a need of digital demand generation and interpersonal interaction at demand generation as well as the testing stage in the form of video counselling. The current banning of MSM dating sites poses a significant barrier to demand generation. Policymakers should advocate for destigmatizing HIV testing and unbanning MSM dating websites so that HIVST demand generation could be fostered through these applications.
