# Supplementary material for: Evaluating the digital dispensation of HIV self-testing (HIVST) among men who have sex with men (MSM) in Pakistan: Seeing through the Consolidated Framework for Implementation Research (CFIR) lens
Source: PLOS Glob Public Health. 2026 Sep 11;6(9):e0005994. doi: 10.1371/journal.pgph.0005994 (PMC13567783; doi:10.1371/journal.pgph.0005994)
Supplement: Supplementary file 1 [file pgph.0005994.s001.pdf]

PLOS Global Public Health

Qualitative evaluation of the digital HIV self-testing (HIVST) intervention among men who have sex with men (MSM) in Pakistan.

--Manuscript Draft--

**Manuscript Number:**	PGPH-D-26-00385
**Article Type:**	Research Article
**Full Title:**	Qualitative evaluation of the digital HIV self-testing (HIVST) intervention among men who have sex with men (MSM) in Pakistan.
**Short Title:**	Qualitative evaluation of the digital HIV self-testing (HIVST) intervention in Pakistan.
**Corresponding Author:**	Usman Ali<br>King Edward Medical College: King Edward Medical University<br>Lahore, PAKISTAN
**Order of Authors:**	Usman Ali
	Safdar Kamal Pasha
	Ramesh Kumar
	Rab Nawaz Samo
	Raza Haider Tirmizi
	Siddique Wali
**Keywords:**	Good health and well-being; gender equality; reduced inequalities
**Abstract:**	HIV self-testing (HIVST) has recently been introduced through an online platform for men who have sex with men (MSM) in Pakistan, where they can order and receive OraQuick HIVST via mail. The current study aimed to explore the experiences of the MSM community who utilize HIVST services through online platforms. Semi-structured interviews with MSM who availed HIVST services were conducted through in-depth interviews (IDI) and focus group discussions (FGDs). Participants were recruited from HIV community-based organizations in Karachi and Lahore. The interviews were coded, and thematic analysis was performed using NVIVO 14.0. Twelve MSM were interviewed in the IDI, and two FGDs were conducted with a total of 12 MSM. Key emergent themes highlighted online ordering as a preferable method for HIV testing, as it bypasses the stigma faced during in-person testing, ensures confidentiality, and provides convenience. Online HIVST ordering is acceptable given regular client support by a community-based organization (CBOs) with the potential to increase HIV testing coverage through peer-to-peer sharing of an online ordering platform. However, participants noted difficulty in platform navigation, confidentiality, and stigma issues by ordering HIVST at home, and a lack of comprehensive condoms, lubricants, and online counselling services. The participants also emphasized the importance of demand generation through social media and dating applications. Provision of a comprehensive service delivery package, digital demand generation through social media, and dating applications is recommended for strengthening online HIVST services.
**Additional Information:**	
**Question**	**Response**
**Financial Disclosure**<br><br>Enter a financial disclosure statement that describes the sources of funding for the work included in this submission and the role the funder(s) played. This includes grants and any commercial funding of the	None to disclose

Powered by Editorial Manager® and ProduXion Manager® from Aries Systems Corporation

work or authors.<br><br>This statement will be typeset if the manuscript is accepted for publication.<br><br>*Please review the submission guidelines and the instructions link below for detailed requirements and guidance.*	
**Competing Interests**<br><br>On behalf of all authors, disclose any competing interests that could be perceived to bias this work.<br><br>This statement will be typeset if the manuscript is accepted for publication.<br><br>*Please review the instructions link below and PLOS Global Public Health's competing interests policy to determine what information must be disclosed at submission.*	None to declare
**Data Availability**<br><br>Before publication, Authors are required to make fully available and without restriction all data underlying their findings. Please see our PLOS Data Policy page for detailed information on this policy.<br><br>A **Data Availability Statement**, detailing where the data can be accessed, is required at first submission. Insert your Data Availability Statement in the box below.<br><br>Please see the data reporting section of our submission guidelines for instructions on what you need to include in your Data Availability Statement.	Available on request

This statement will be typeset if the manuscript is accepted for publication.<br><br>*PLOS allows rare exemptions to address legal and ethical concerns. If you have legal or ethical restrictions, please use the box below to detail these in full sentences for the Journal team to consider.*	

Powered by Editorial Manager® and ProduXion Manager® from Aries Systems Corporation

Manuscript

Click here to access/download;Manuscript;Version with author details.docx

Qualitative evaluation of the digital HIV self-testing (HIVST) intervention among men who have sex with men (MSM) in Pakistan.

Usman Ali[1] , Muhammad Safdar Kamal Pasha[2], Ramesh Kumar[3], Rab Nawaz Samo[4], Raza Haider Tirmizi[5], Siddique Wali[6]

1. Research Consultant, Dostana, Lahore, Pakistan
   Usman Ali (Corresponding Author)
   ORIC ID: 0000-0001-9168-5911
   Email: econtactusman@gmail.com
2. PhD Scholar, Health Services Academy, Pakistan
   Email: mpasha@who.int
3. Professor of Public Health, Health Services Academy, Pakistan
   Email: drramesh1978@gmail.com
4. Program Officer, HIV, United Nations Development Program, Pakistan
   Email: rabnawaz.samo@undp.org
5. Syed Raza Haider Tirmizi
   Project Director, Dostana, Lahore
   Email: syedraza.tir@gmail.com
6. Siddique Wali
   Project Director, Humraz, Karachi
   Email: Siddique.wali@hmhs.org.pk

Acknowledgements

We acknowledge Dostana and Humraz for their generous help in participant recruitment and in facilitating data collection for this study.

Disclosure

The authors (s) declare no potential conflicts of interest concerning the research, authorship, and/or publication of this article.

Funding

The authors (s) received no financial support for the research, authorship, and/or publication of this article.

Ethical statement

Ethics Statement

Ethical approval for this study was obtained from the Health Services Academy, Islamabad (No. Fr.No. 000 I 7/l ISA/PhD-2023l Dated 8 Jan 2025

Abstract

HIV self-testing (HIVST) has recently been introduced through an online platform for men who have sex with men (MSM) in Pakistan, where they can order and receive OraQuick HIVST via mail. The current study aimed to explore the experiences of the MSM community who utilize HIVST services through online platforms. Semi-structured interviews with MSM who availed HIVST services were conducted through in-depth interviews (IDI) and focus group discussions (FGDs). Participants were recruited from HIV community-based organizations in Karachi and Lahore. The interviews were coded, and thematic analysis was performed using NVIVO 14.0. Twelve MSM were interviewed in the IDI, and two FGDs were conducted with a total of 12 MSM. Key emergent themes highlighted online ordering as a preferable method for HIV testing, as it bypasses the stigma faced during in-person testing, ensures confidentiality, and provides convenience. Online HIVST ordering is acceptable given regular client support by a community-based organization (CBOs) with the potential to increase HIV testing coverage through peer-to-peer sharing of an online ordering platform. However, participants noted difficulty in platform navigation, confidentiality, and stigma issues by ordering HIVST at home, and a lack of comprehensive condoms, lubricants, and online counselling services. The participants also emphasized the importance of demand generation through social media and dating applications. Provision of a comprehensive service delivery package, digital demand generation through social media, and dating applications is recommended for strengthening online HIVST services.

~~**Keywords:**~~

Good health and well-being; gender equality; reduced inequalities

Introduction

Men who have sex with men (MSM) are one of the five key populations at risk for HIV infection.(AIDS, Crisis and the Power to Transform: UNAIDS Global AIDS Update, 2025) Globally, 7.6% of MSM live with HIV, which is ten times the global prevalence of HIV in the general population.(AIDS, Crisis and the Power to Transform: UNAIDS Global AIDS Update, 2025) MSM are stigmatized and discriminated against in various jurisdictions. Around 63 countries have some law that prohibits same-sex relationships, with 12 countries proposing capital punishment for same-sex relationships.(AIDS, Crisis and the Power to Transform: UNAIDS Global AIDS Update, 2025; Human Dignity Trust, 2024) Globally, 40.5% of MSM have reported facing stigma in the last 12 months.(Hear Us out: Community HIV Measuring HIV-Related Stigma and Discrimination, 2023)

Pakistan is also one of the 12 countries that proposed capital punishment for MSM under Section 377 of the Pakistan Penal Code.(Usman Ali, 2024) Thus, MSM are a marginalized population at risk of HIV infection. It is estimated that of the PLHIV pool in the country, 25% are identified as MSM.(Pakistan AIDS Strategy IV 2021-2025, 2020) The prevalence of HIV infection among MSM in the country is 5.4%.(Integrated Biological and Behavioral Surveillance of HIV in Pakistan: Round 5 - 2016-17, 2017) It should be noted that the prevalence of HIV in the general population is 0.1%.(Integrated Biological and Behavioral Surveillance of HIV in Pakistan: Round 5 - 2016-17, 2017) Despite the high prevalence of HIV, extending HIV prevention services to MSM is difficult because of social stigma and their marginalized status. According to the Pakistan AIDS Strategy, only 9% of MSM had access to HIV prevention and 6% had access to HIV testing in 2019.(Pakistan AIDS Strategy IV 2021-2025, 2020) This is in contrast to the United Nations Goal of 90-90-90 by 2020, which stated that 90% of people know their HIV status; that is, they have been tested and counseled on their HIV status.(End Inequalities, End AIDS. Global AIDS Strategy 2021-2026, 2020)

To curtail the HIV epidemic in MSM, Pakistan's National AIDS Strategy has focused on establishing community-based organizations (CBOs) for MSM that provide in-person HIV prevention and testing services.(Ali & Ali, 2023; End Inequalities, End AIDS. Global AIDS Strategy 2021-2026, 2020) However, in recent years, there has been an increasing shift in the MSM community to digital spaces to find sexual ~~partners.(Ali et al., 2023)~~ This includes dating applications and social media platforms. In a feasibility study on HIV pre-exposure prophylaxis

(PrEP), 65.7% of MSM cruised partners through dating applications, whereas 34.7% used social media applications.(Ali et al., 2023) As a result of this increasing use of digital spaces by MSM to socialize, the HIV prevention program aimed to launch a digital intervention for the MSM community in two major cities: Lahore and Karachi. Two online platforms were developed, with one providing information on HIV prevention, treatment, and facilities, called the Sehat Dost application (Figure 1; www.sehatdost.pk). Sehat Dost was linked to QuickRes (www.quickres.org). Quickres is a global platform used to make appointments and reservations for HIV services.(EpiC, 2020) The services provided in Pakistan through the platform include delivery of oral fluid-based HIV self-testing (HIVST). After an appointment for HIVST is booked, oral fluid-based HIVST (OraQuick) is delivered to the community in Lahore and Karachi. To generate demand for HIV services, advertisements are made in CBO drop-in-centers (DIC) as well as messaging on WhatsApp, which includes a package of two videos, one focusing on how to perform HIVST and the other on how to use online platforms.

The current study aimed to explore the experiences of the MSM community who utilize HIV self-testing services through these online platforms.

Figure 1. Key modules on the Sehat Dost platform.

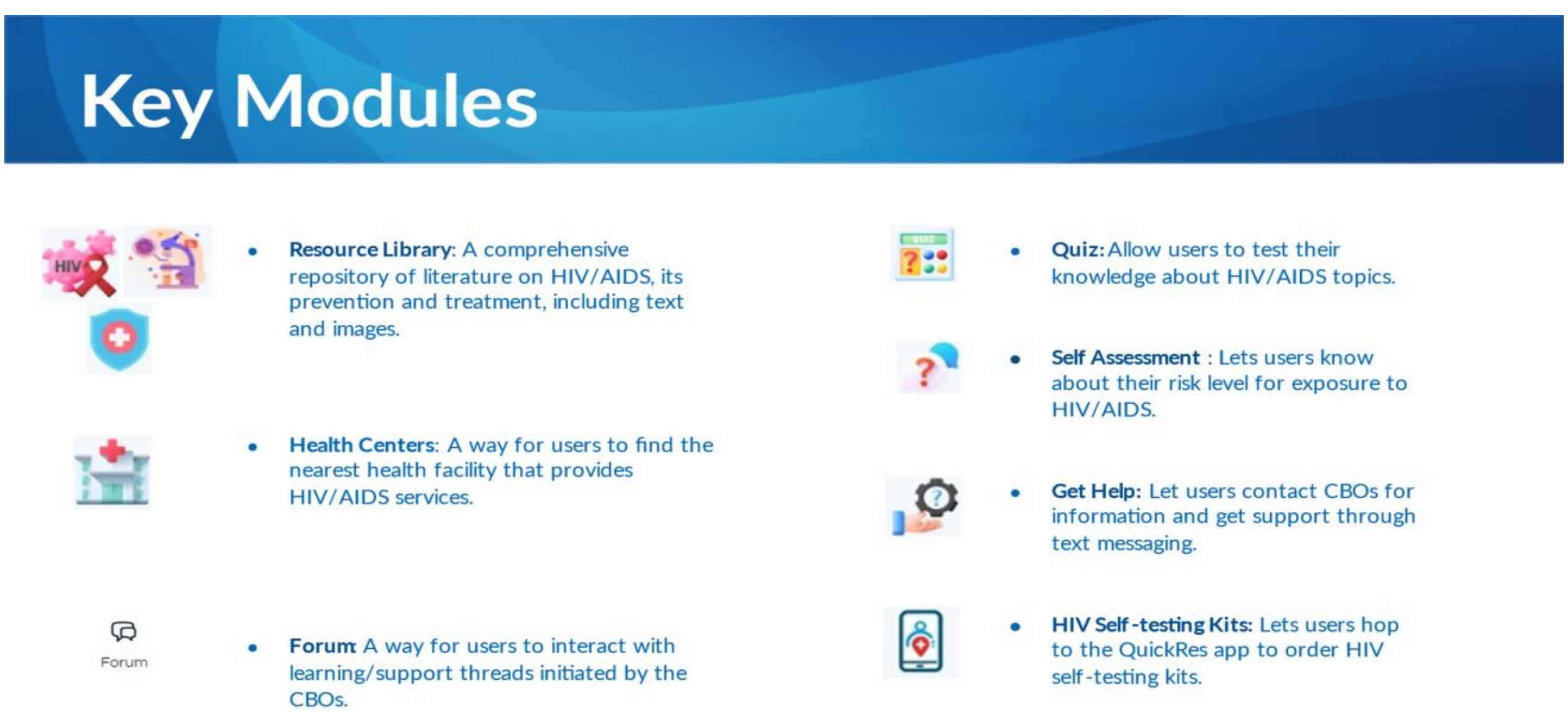

Materials and Methods

This qualitative exploratory study was conducted from May 2025 to July 2025 in Lahore and Karachi.

Ethics Statement

Ethical approval for this study was obtained from the Health Services Academy of Islamabad (No. 00017/HSA/PhD-2023, Dated 8 Jan 2025)

Participants

People above 18 years of age, male, identified as men who have sex with men, who have received both in-person and online HIV testing services, were identified and recruited by the outreach workers of two CBOs: one based in Lahore (Dostana) and one based in Karachi (Humraz). CBO workers and those who had been diagnosed with HIV were excluded from the study. Both CBOs provided services only to the MSM community. Convenient non-probability sampling was performed. Informed written consent was taken.

Interviews and the data collection process

Once eligible participants were recruited, they were given the option of being interviewed in individual in-depth interviews (IDI) or focus group discussions (FGD). Informed consent was obtained, and the participants were interviewed in person by a lead investigator using an interview guide. Semi-structured interviews were also conducted. Participants were asked about their experience with digital platforms for HIVST, how it compares with in-person HIV testing at the CBO, perceived barriers, and benefits of online HIVST. The interviews were conducted in Urdu, the national language of the country. Audio from interviews was recorded on a personal device, followed by manual transcription and English translation. To ensure confidentiality, the data were anonymized by assigning codes to individual participants, such as MSM1 and MSM2. Only the interview personnel had access to anonymous data.

Data analysis

The transcripts were imported into NVIVO version 14.0. (Dhakal, 2022) The data were coded using descriptive and interpretation-focused coding. Key emergent themes were identified in an iterative process through discussion between the two authors (AU and SKP). Thematic analysis

was performed as described by Adu et al, with consensus among both authors followed by review by RK.(Adu & Philip, 2019). The results were reported using Consolidated Criteria for Reporting Qualitative Studies (COREQ). (Tong et al., 2007)

Reflexivity Statement

The lead investigator has been working in HIV for nearly seven years, whereas SKP has been working for nearly two decades. AU has been involved in community research and various qualitative projects, where SKP has worked at the national level in HIV response with various agencies. Both coded the data after mutual discussions. RK has worked in public health for over two decades, providing an overview of the data coding and analysis processes.

Results

Twelve in-depth interviews (IDI) were conducted in total. All participants had at least intermediate (12 years of education). All except one were employed. All participants had their first HIVST (index) in a drop-in center (DIC). The summary characteristics of the participants interviewed through the IDI are given in the table below.

Table I. Characteristics of in-depth interviews, n=12.

Serial no.	Participant ID	Age in years	Education	Occupation	Income in PKR	Index HIVST
1.	MSM1	25	Bachelors	Lawyer	70000	DIC
2.	MSM2	23	Bachelors	Veterinary doctor	60,000	DIC
3.	MSM3	21	Bachelors	Freelance advertiser	100,000	DIC
4.	MSM4	21	Bachelors	Unemployed	0	DIC
5.	MSM5	22	Intermediate	Fashion designer	40,000	DIC
6.	MSM6	28	Bachelors	Paramedic	25000	DIC
7.	MSM7	23	Bachelors	Part-time employee	25,000	DIC
8.	MSM8	47	Masters	Artist	100,000	DIC

9.	MSM9	24	Bachelors	Advertising agency	140,000	DIC
10.	MSM10	25	Bachelors	Office boy	45,000	DIC
11.	MSM11	27	Masters	Display designer	100,000	DIC
12.	MSM12	28	Bachelors	Sex work/ office work	45,000	DIC

In addition, two focus group discussions were conducted with 12 MSM. The mean age of the participants was 25.85 years. All participants had at least matriculated. Three of the 12 participants were unemployed. The mean age of participants was 24,500 PKR. The summary characteristics of the participants interviewed through FGD are presented in the table below.

Table II. Characteristics of participants interviewed in the focus group discussion (FGD), n=12

**Serial no.**	**Characteristics**	**Frequency (Percentage)**
1.	Mean age of participants in years	25.85
2.	Education	
	No education	0 (0%)
	Under Matric	0 (0%)
	Matric to Intermediate	9 (75%)
	Graduation and above	3 (25%)
3.	Employment status	
	Employed	9 (75%)
	Unemployed	3 (25%)
4.	Mean income of participants in Pakistani Rupees	24,500

Based on the thematic analysis of the qualitative interviews, five themes emerged, along with eleven subthemes. The table below presents the thematic analysis.

Theme 1. Benefits of HIVST delivery to community individuals

This theme covers the self-perceived benefits of the intervention. This theme includes three subthemes. Overall, the theme reflects reasons for preferring the online ordering of HIVST in comparison with HIVST in the CBO.

Subtheme 1.1. Prefer online ordering of HIVST to avoid the stigma faced in the CBO

One self-perceived benefit of online ordering of HIVST is that it helps avoid the stigma faced by the CBO. Participants reported that they felt stigmatized by getting HIVST in the CBO drop-in center. One participant stated his fears about running into someone whom they knew in their personal life, but not out to them as MSM.

…because a lot of people still don't understand how to respond sensitively to this kind of thing (HIV). So there's fear. There is no issue coming here (CBO), but home is better. No fear of running into someone, no awkward eye contact. FGD 1

Yet another MSM stated that the stigma attached to HIV testing was one reason for preferring to order HIVST through the online platform at home.

Here, it's like you feel hesitant… But if it's positive, how will people treat you? Even with a negative result, how will people respond? On the other hand, if you do it on your own, in a personal space, you don't feel like you're sitting alone; there's just no one there to judge you. Also, if someone doesn't feel comfortable coming here, it's better for them to take the test on their own and see the results privately. Then they can decide how to proceed from there. MSM7, 23 years old, part time employee, 14 years of education

Subtheme 1.2. Online HIVST ordering allows maintaining confidentiality of HIV status and sexuality

Related the subtheme 1.1, the community stated a benefit of placing the order of HIVST online as it offers more confidentiality both in terms of sexuality and in terms of one's HIV status. The participants stated that in the DIC, others can judge the results of their HIV test through facial expressions, and thus, may lead to a lack of confidentiality, and if the result is positive, it leads to stigma. One participant stated the following:

Because at home, no one else is around in that moment. But if you're doing the test here at the center, your facial expressions and the way you react can give something away. If you get tense or

scared, people around you who are watching might notice. They'll realize you're doing an HIV self-test and might assume the result is bad. MSM7, 23 years old, part time employee, 14 years of education

In comparison with a rapid diagnostic test conducted by a healthcare provider, the participants stated that they preferred placing an online order because they wanted to keep their details confidential from the providers (in comparison to the participants above, who wanted to keep their HIV status and sexuality confidential from community-visiting CBOs.

When we come here, we have to first go to the office and get our ID. Then we visit the doctor, he's a paramedic, and he conducts the test. But at home, we do it ourselves. We already know how. In Punjabi, we say "don't open your mouth… meaning, just do it quietly and easily."FGD1

Another participant stated that those who are cautious about revealing their sexuality or who have social anxiety may prefer placing an online order.

Some people hesitate a lot while coming to the NGO. They don't want to accept (their sexuality) for whatever reason they have. So, self-testing is very easy for them. They can test themselves at their place or anywhere. Some people have social anxiety; they don't want to face it. They don't know what will happen if they come here. So, I think it is very good for them and for me too. Because my house is quite far. It's not easy for me to visit the NGO. So, I also do self-testing. MSM4, 21 years old, graduate, unemployed

Subtheme 1.3. Online ordering of HIVST is more convenient

The participants also highlighted that ordering HIVST online is convenient. This is because they do not have to take time out of their routine and place order. Thus, the online order saves time. The other reason for ordering HIVST online was the travel expenses they had to bear while visiting the CBO, which were saved as the CBO delivered HIVST free of cost. One participant during the FGD stated that

I think it has a lot of benefits, because sometimes you don't have money to go to the NGO. They provide it free of cost, including delivery, which is very convenient. You get it for free. Many friends are busy during the day and don't have time. FGD 1

Another participant from the second FGD stated

Yes, I had done the test before. But because of my busy schedule with university and classes it was a bit difficult to come here. So doing it at home was much easier for me. I ordered it through a link from an online platform. …. I received the kit, and I did the test myself. I rubbed the swab on my gums and then dipped it, just like the instructions said. FGD2

Theme 2. Acceptability of the online intervention

Subtheme 2.1. WhatsApp messaging, complemented by an easy user interface, makes the online platform easy to use

As mentioned above, the current structure of the intervention is the demand generation of HIVST by sharing instructions in the form of videos on how to operate and place an order on the online platform and a video on how to perform HIVST. During the FGD, the participant stated:

It is very good. I had no idea about the website. But the video has made it easy for us. We have sent it to many friends. It is very good. It has a lot of clarity about how to use the website in Urdu, what questions are there, and how to do it. It is very easy. It has all the to-the-point information. FGD1

Another participant highlighted that sending videos on WhatsApp made it easy to use the online platform.

If you want to book a kit online, then you have to go to QuickRes, right? Now, they also send videos. Like, XX (CBO worker) who sent me the video, he told me that if you want to tell someone further, then first send him a video so that he understands what it is, how to use it. So, sending a video is very beneficial. I also sent a link to him. There are some Facebook friends with whom I have never met, but there is a lot of talk on Facebook. MSM6, 26 years old, paramedic by profession, 14 years of education

The participants acknowledged that the use of the Urdu language made navigation easier on an online platform.

No, it was very smooth. The website had a full Urdu language option. I didn't even know it could be done online until a friend told me. FGD2

However, these findings were not universal, and some gaps were highlighted by the participants, as given in Theme 5.

Theme 3. Potential benefit of digital intervention on community health as a whole

Subtheme 3.1 Online ordering of HIVST can increase HIV testing coverage

Beyond personal benefits, digital intervention has a broader impact on community health. Participants who were not healthcare staff of the CBO stated that they can now provide a link to services to other MSM, who have not yet been reached by the CBOs, and would refrain from getting in touch with the CBO staff because of stigma and maintain privacy. The potential of this digital intervention is recognized by one MSM, who stated that:

If we limit it to CBOs, then it will not reach everyone. MSM are those who don't believe in themselves. It is a good thing to reach them. MSM4, 21 years old, graduate, unemployed

Another participant recalled his experience of getting his friends tested through this service

I feel that the test done here is different from the one done at home. The test we do at home is private. If we don't want to share our result, we don't have to tell anyone. And we still get to know what our result is. When two of my friends visited me, I got their tests done at home. MSM5, 22 years old, fashion designer by profession with 12 years of education

Another participant recalled a similar experience

I found out through an XX worker (at the CBO) that you can even get it (HIVST) delivered to your home. Then I passed that on to my friends I guided them too. I explained how to use the kit, how the process works. I showed them everything step by step. MSM6, 26 years old, paramedic by profession, 14 years of education

Theme 4. Perceived stigma and discrimination while placing the online HIVST order

Digital order for HIVST bypasses the stigma faced by individuals in CBO settings. The MSM community faces a similar stigma while ordering HIVST at home and has similar confidentiality issues.

Subtheme 4.1. Perceived stigma while ordering HIVST at home

The participants stated that they ordered HIVST not at home, but in the vicinity, fearing questioning from family members.

Many clients do not give the address of their house, but give the pin location of their house. For example, if there is a shop or a famous thing, if there is a shop a little ahead of their house, the rider goes there and calls them. We call the client before sending the kit to confirm the timing and the schedule. FGD 1

Speaking about stigma, and lack of privacy at home, one participant stated that

That fear is always there that your privacy might be exposed. And if it does, your reputation (as a straight man) could be tarnished. You have your whole family whose prestige is at stake it's not just about you. MSM8, 47 years old, artist by profession, 16 years of education

Another participant in the FGD said that

Yes, absolutely the first thing that comes to mind is where should I have it delivered? Because I can't have it sent to my home. So I try to make sure it's delivered somewhere far from home. Apart from that, I worry about how it will be. It's difficult for a newbie, but for a guide, it's not an issue. FGD2

Compared with HIVST at home and in DIC, one MSM stated that

Here, the environment is friendly. It feels easier and more comfortable. At home, the family doesn't know about this. You have to keep the testing private from them. Naturally, we can't tell them. Some families are friendly, but in some households, you just can't share things like this. I don't think anyone would openly share something like this with their family. The good thing here is that there are friends around at the center, and the environment is friendly like home, but better. Still, if you think about it, going to and from the center takes time. At home, you can just take the test. MSM2, 23 years old, Veterinary doctor by profession

Subtheme 4.2. Confidentiality issues at home

Interlinked with the issue of perceived stigma are the intrusiveness of family members and a lack of confidentiality. MSM stated that they feared that their family might open the HIVST parcel delivered at home. Family members may later question why they use HIVST. A participant said that

I don't think anyone can hide it and keep it at home. If he uses a kit, they (family) will check it. He should not keep it in front of his family and face their questions. MSM2, 23 years old, Veterinary doctor by profession

Another MSM stated that

No matter what kind of packaging you use, if someone in the family even has the slightest doubt, they'll open it. Either that, or your reputation at home needs to be so solid that your privacy is respected. MSM8, 47 years old, artist by profession, 16 years of education

However, this lack of privacy and perceived stigma are not universal. One participant highlighted while talking about HIV and self-testing.

Even if you get it delivered to your home, there's no issue because this is common. It's not a gay disease or anything. It can happen randomly, even if you just go to a barber, their instruments can also be a source. This is a disease that can be contracted in many ways. So it's not necessary that family members find out about it just because it's ordered at home. MSM9, 24 years old, working in an advertising agency, 14 years of education

Theme 5. Gaps and recommendations for strengthening digital intervention

Some participants stated that the use of video messaging made online platform navigation easy. Some participants highlighted these gaps. These were sorted into two sub-themes. Subtheme 5.1 pertains to the design and structure of the online platform, and Subtheme 5.2 pertains to the content of the platform.

Subtheme 5.1. Problems related to the design and structure of the online platform

Participants identified several issues related to the design and structure of online platforms. The most important of these platforms is their presence. One for information on HIV, including the use of condoms, lubricants, etc., and one for HIV. The online platform is described by some participants as difficult to navigate and confusing. One participant stated the following:

Basically, its link was very difficult. I want that if a person who does not know how to write or read goes to their link, and have an option to call. Call and get help. Not everyone knows how to write. People are uneducated. You already know how low the literacy rate is. For someone who can't read or write properly, ordering a kit online can be really difficult. This platform isn't really for

the average person; only someone who's a graduate or at least well-educated can manage to place an order. An uneducated or less literate person wouldn't be able to do it easily. Only graduates can do this. But there should also be an option to call. You should call and receive a call from the representative. You should tell him all the details, your address, and it should reach you. FGD2

Since HIVST can be ordered from Quickres, but HIV prevention information is on the Sehat Dost platform, it may be cumbersome for some participants, as one participant stated:

As far as I know, both things should be on the same platform….So, I will not need to visit other platforms.. MSM6, 26 years old, paramedic by profession, 14 years of education

Another participant highlighted that even among the two platforms, the linkage is inadequate

No, there's no option for Sehat Dost in QuickRes. The QuickRes option is available on Sehat Dost, but not vice versa. FGD2

This lack of linkage between platforms causes people to miss content on the complementary platform.

Some people know about it, and some don't. But it depends on the chain. As I know, if someone consults me, I will guide them properly. MSM6, 26 years old, paramedic by profession, 14 years of education

Apart from having two platforms as part of the digital intervention, participants also highlighted some technical problems related to bookings. One participant stated the following:

There are time slots shown in the beginning you're asked to select one for delivery. Different time slots appear, and you're supposed to choose one. But at first, they don't get selected. Then, when you move to the next page, it shows that the slots are available, but on the next screen, they aren't. The system just assigns a random time on its own sometimes at a time when you're not available or there is any problem, then… MSM1, 25 years old, lawyer by profession

Subtheme 5.2. Problems related to the content of the platform

Some participants also criticized the platform for asking for too much information before booking.

I saw their questions; there were quite a lot, maybe 2–3 pages worth. They were very detailed. So either the questions should be shortened a bit, or made more efficient, so that it saves people time. MSM5, 22 years old, fashion designer by profession, with 12 years of education

Speaking about the technical language used on the platform, one participant stated that

There are a few questions that confuse people, like MSW, MSM, or bisexual. When we explain that these terms are for the community and that there's nothing to be afraid of and that their data is safe, then they're more comfortable. Some people did ask me what MSW, MSM, or bisexual means. These are just written differently, and people often get confused. But it's not a problem once it's explained, they're fine. Yes, people are a little afraid at first. FGD2

Subtheme 5.3. Community recommends the provision of comprehensive services through online order

We also asked participants about any services they thought should be included in the current package of services through digital intervention. The participants stated that comprehensive services should be provided. This includes the provision of condoms and counselling services.

One participant stated that

At least when it comes to medication, you could say that it can't be provided without a prescription, so you have to go through that process. But when it comes to condoms or lubricants, I believe those should be made available. Otherwise, if you look at how it's done in other countries, you either upload a prescription or hand it over directly. They usually have a system; for example, if someone provides a phone number, it gets linked to their data, and everything is saved. So based on that data, your medication is sent to you directly. MSM 2

Similarly, another participant stated that

And apart from that, there should be an option for condoms as well. For example, if someone wants to use condoms but doesn't feel comfortable asking for them openly, then they should be able to get them delivered discreetly to their home, too. That would also be very beneficial in terms of protection. MSM6, 26 years old, paramedic by profession, 14 years of education

Other participants recommended online counselling. One participant suggested counselling online with a counsellor

It is an online service, and they are providing a kit, so counselling should also be done as the kits are provided. There can be online counselling, and it can also be done via video call. Now, obviously, it is the age of digital. If there can be online classes, online meetings. So why can't counselling be done online? MSM 2

Subtheme 5.4. Demand generation through personal and community interactions, as well as online means such as dating applications and social media

The interviewed community members provided recommendations on demand generation for digital services. Few have recommended sharing links to digital platforms after personal communication, whereas others have recommended the use of social media and dating applications for this purpose.

One participant stated that

We can't send it through just by sharing the link, as it is sensitive. You can only share it after a deep conversation. Only then will the next person understand it and be able to use it positively. You can also give advertisements on Grindr and other dating applications. FGD1

While some people have stressed the use of dating applications for advertising, others have stated the use of social media applications for the demand generation of HIV services and self-testing.

Not everyone uses Grindr. For example, I don't use Grindr. Most people use Instagram and Facebook (for hookups and dates). Even within the gay community, not everyone is on Grindr. MSM7, 23 years old, part-time employee, 14 years of education

One participant stated that there should be group sessions to complement digital messaging, and stated that people may take digital messaging for granted.

There should be more awareness sessions in the CBOs, too. They should hold regular sessions so people can learn. Because most people don't read everything on the app. They either skip through or just don't bother going into full detail. FGD2

Discussion

To the best of our knowledge, the current study is the second study that evaluated digital HIVST in a Muslim-majority country. A previous study conducted in Malaysia evaluated a digital

application that provided a comprehensive HIV prevention package, including HIVST and pre-exposure prophylaxis (PrEP).(Shrestha et al., 2023) This study employed a pre-post design. Among 50 MSM using the application, 84% used HIVST, with 42% ordering more than once.(Shrestha et al., 2023) Globally, HIVST delivery through digital platforms is acceptable and accurate.(Beecroft et al., 2025) Zhu et al. conducted a randomized controlled trial comparing HIVST delivery through digital ordering and in-person dispensation. The study showed that the option of digital ordering increased HIV testing two-fold.(Zhu et al., 2019) In two separate RCTs that used Internet-based HIVST dispensation, it was noted that testing rates increased 1.47 to 1.77-fold. (Young et al., 2022)(Wang et al., 2018)However, intervention design in Pakistan has major limitations. One of these is the lack of comprehensive HIV prevention services. According to the World Health Organization, comprehensive HIV prevention services include HIV testing services, condoms, PrEP, and the management of sexually transmitted infections.(Consolidated Guidelines on HIV, Viral Hepatitis and STI Prevention, Diagnosis, Treatment and Care for Key Populations, 2022) While information on these is available on the Sehat Dost platform, participants highlighted that the presence of a separate platform for HIVST ordering (Quickres) may be cumbersome to use.

Previously, two community pilot projects were evaluated for HIVST using OraQuick, one each for MSM and transgender individuals.(Altaf et al., 2022, 2023) Both pilot projects involved the dispensation of HIVST through outreach workers based on CBOs. Among transgender individuals, 90.6% reported that HIVST was easy to perform and 95% stated that the instructions for HIVST were easy. Approximately 95% of transgender individuals in the study stated that they would recommend HIVST to community friends.(Altaf et al., 2022) Although the current model of intervention relies on digital ordering and mailing of HIVST, participants stated that they shared the link of the online ordering platform with other community members. It should be noted that this method may be used to engage MSM who cannot be reached by conventional outreach by CBOs. In terms of demand generation, the participants in the current study emphasized digital means. This includes the use of social media applications, which may be used for various purposes but may include cruising clients or dates. These platforms include Facebook and Instagram. However, the participants also highlighted the use of digital dating applications exclusively by MSM, such as Grindr or Blued. In the previous pilot of HIVST, which relied on in-person HIVST dispensation, 55.3% of MSM preferred social media, while 45.3% preferred peer-to-peer methods

for demand generation.(Altaf et al., 2023) While the profile and risk behaviors of MSM who use dating applications have not been studied in Pakistan, it is not known if their risk behaviors are higher than those of MSM who do not use dating applications. Researchers from the United States have shown that MSM using Grindr had higher rates of chlamydia or gonorrhea infection than those who did not use Grindr, higher rates of HIV infection, condomless anal intercourse, and a higher number of male sexual partners. However, the cohort was more likely to initiate HIV PrEP than non-users.(Hoenigl et al., 2020) This highlights the systematic differences among MSM who use dating applications. Thus, the digital dispensation of HIVST can be an effective method for engaging this community by linking Quickres with dating applications for demand generation. However, it should also be noted that dating applications are banned in Pakistan by the government; thus, advertising on these applications may require unbanning of the applications. Despite being banned by the government, proxy web administrators use these applications.(Gibran Naiyyar Peshimam, 2020)

The current study has several limitations. First, it explored the qualitative experiences of the participants. In addition, participants were recruited through non-probability sampling using CBO staff, thus introducing selection bias. The participants were from two cities in Pakistan; thus, their experience may not be generalizable to all MSM residing in the country.

Conclusion

Online HIVST ordering is acceptable given regular client support by a community-based organization (CBOs) with the potential to increase HIV testing coverage through peer-to-peer sharing of an online ordering platform. However, participants noted difficulty in platform navigation, confidentiality, and stigma issues by ordering HIVST at home, and a lack of comprehensive condoms, lubricants, and online counselling services. The participants also highlighted the need for demand generation through social media and dating applications. Provision of a comprehensive service delivery package, digital demand generation through social media, and dating applications is recommended for strengthening online HIVST services.

Authors contribution

AU, SKP: Literature search, conceived, designed and supervised the study. Responsible and accountable for the accuracy and integrity of data.

RK, RS: Transcription and Critical Review.

SRHT and MSW: Data collection analysis

All authors have read and approved the final version

References

Adu, & Philip. (2019). *A Step-by-Step Guide to Qualitative Data Coding*.

AIDS, crisis and the power to transform: UNAIDS Global AIDS Update. (2025). http://www.wipo.int/amc/en/mediation/rules

Ali, U., & Ali, S. A. (2023). Prevalence of Depression and Subjective Job Stress Among Men who have Sex with Men and Transgender Community Health Workers in Pakistan. *Journal of the College of Physicians and Surgeons Pakistan*, *33*(7), 779–783. https://doi.org/10.29271/jcpsp.2023.07.779

Ali, U., Pasha, S. K., Patel, R., Schmidt, H. M., & Altaf, A. (2023). Assessment of Acceptability of Pre-exposure Prophylaxis for HIV Prevention Among Men Who Have Sex with Men and Transgender Community in Pakistan. *Journal of the College of Physicians and Surgeons Pakistan*, *33*(9), 983–989. https://doi.org/10.29271/jcpsp.2023.09.983

Altaf, A., Pasha, M. S. K., Majeed, A., Ali, W., Alaama, A. S., & Jamil, M. S. (2022). Acceptability and feasibility of HIV self-testing among transgender people in Larkana, Pakistan: Results from a pilot project. *PloS One*, *17*(7), e0270857. https://doi.org/10.1371/journal.pone.0270857

Altaf, A., Pasha, M. S. K., Wali, M. S., & Majeed, A. (2023). Acceptability and feasibility of intra-peer and social media approach for HIV self-testing among men who have sex with men in Karachi: lessons from a pilot study. *Journal of the Pakistan Medical Association*, *73*(3). https://doi.org/10.47391/JPMA.6846

Beecroft, A., Vaikla, O., Engel, N., Duchaine, T., Liang, C., & Pant Pai, N. (2025). Evidence on Digital HIV Self-Testing From Accuracy to Impact: Updated Systematic Review. *Journal of Medical Internet Research*, *27*, e63110. https://doi.org/10.2196/63110

Consolidated guidelines on HIV, viral hepatitis and STI prevention, diagnosis, treatment and care for key populations. (2022).

Dhakal, K. (2022). NVivo. *Journal of the Medical Library Association*, *110*(2). https://doi.org/10.5195/jmla.2022.1271

End inequalities, end AIDS. Global AIDS Strategy 2021-2026. (2020). https://www.unaids.org/sites/default/files/PCBSS_Global_AIDS_Strategy_2021--2026_EN.pdf

EpiC. (2020). *QuickRes Technical Guide; A Global Online Reservation and Case Management Tool for HIV Programs*. https://quickres.org

Gibran Naiyyar Peshimam. (2020, September 2). Pakistan blocks five dating apps including Tinder and Grindr. *Reuters*.

Hear us out: Community HIV measuring HIV-related stigma and discrimination. (2023). https://www.stigmaindex.org/wp-content/uploads/2023/11/PLHIV-Stigma-Index-Global-Report-2023-2.pdf

Hoenigl, M., Little, S. J., Grelotti, D., Skaathun, B., Wagner, G. A., Weibel, N., Stockman, J. K., & Smith, D. M. (2020). Grindr Users Take More Risks, but Are More Open to Human Immunodeficiency Virus (HIV) Pre-exposure Prophylaxis: Could This Dating App Provide a Platform for HIV Prevention Outreach? *Clinical Infectious Diseases*, *71*(7), e135–e140. https://doi.org/10.1093/cid/ciz1093

Human Dignity Trust. (2024). *Map of Jurisdictions that Criminalise LGBT People*. Https://Www.Humandignitytrust.Org/.

Integrated Biological and Behavioral Surveillance of HIV in Pakistan: Round 5 - 2016-17. (2017).

Pakistan AIDS Strategy IV 2021-2025. (2020).

Shrestha, R., Altice, F. L., Khati, A., Azwa, I., Gautam, K., Gupta, S., Sullivan, P. S., Ni, Z., Kamarulzaman, A., Phiphatkunarnon, P., & Wickersham, J. A. (2023). Clinic-Integrated Smartphone App (JomPrEP) to Improve Uptake of HIV Testing and Pre-exposure Prophylaxis Among Men Who Have Sex With Men in Malaysia: Mixed Methods Evaluation of Usability and Acceptability. *JMIR MHealth and UHealth*, *11*, e44468. https://doi.org/10.2196/44468

Tong, A., Sainsbury, P., & Craig, J. (2007). Consolidated criteria for reporting qualitative research (COREQ): a 32-item checklist for interviews and focus groups. *International Journal for Quality in Health Care*, *19*(6), 349–357. https://doi.org/10.1093/intqhc/mzm042

Usman Ali. (2024). *National HIV Stigma Index*.

Wang, Z., Lau, J. T. F., Ip, M., Ho, S. P. Y., Mo, P. K. H., Latkin, C., Ma, Y. L., & Kim, Y. (2018). A Randomized Controlled Trial Evaluating Efficacy of Promoting a Home-Based HIV Self-Testing with Online Counseling on Increasing HIV Testing Among Men Who Have Sex with Men. *AIDS and Behavior*, *22*(1), 190–201. https://doi.org/10.1007/s10461-017-1887-2

Young, S. D., Cumberland, W. G., Singh, P., & Coates, T. (2022). A Peer-Led Online Community to Increase HIV Self-Testing Among African American and Latinx MSM: A Randomized Controlled Trial. *Journal of Acquired Immune Deficiency Syndromes*, *90*(1), 20–26. https://doi.org/10.1097/QAI.0000000000002919

Zhu, X., Zhang, W., Operario, D., Zhao, Y., Shi, A., Zhang, Z., Gao, P., Perez, A., Wang, J., Zaller, N., Yang, C., Sun, Y., & Zhang, H. (2019). Effects of a Mobile Health Intervention to Promote HIV Self-testing with MSM in China: A Randomized Controlled Trial. *AIDS and Behavior*, *23*(11), 3129–3139. https://doi.org/10.1007/s10461-019-02452-5

Figure 1 Click here to access/download;Figure;Figure `.png

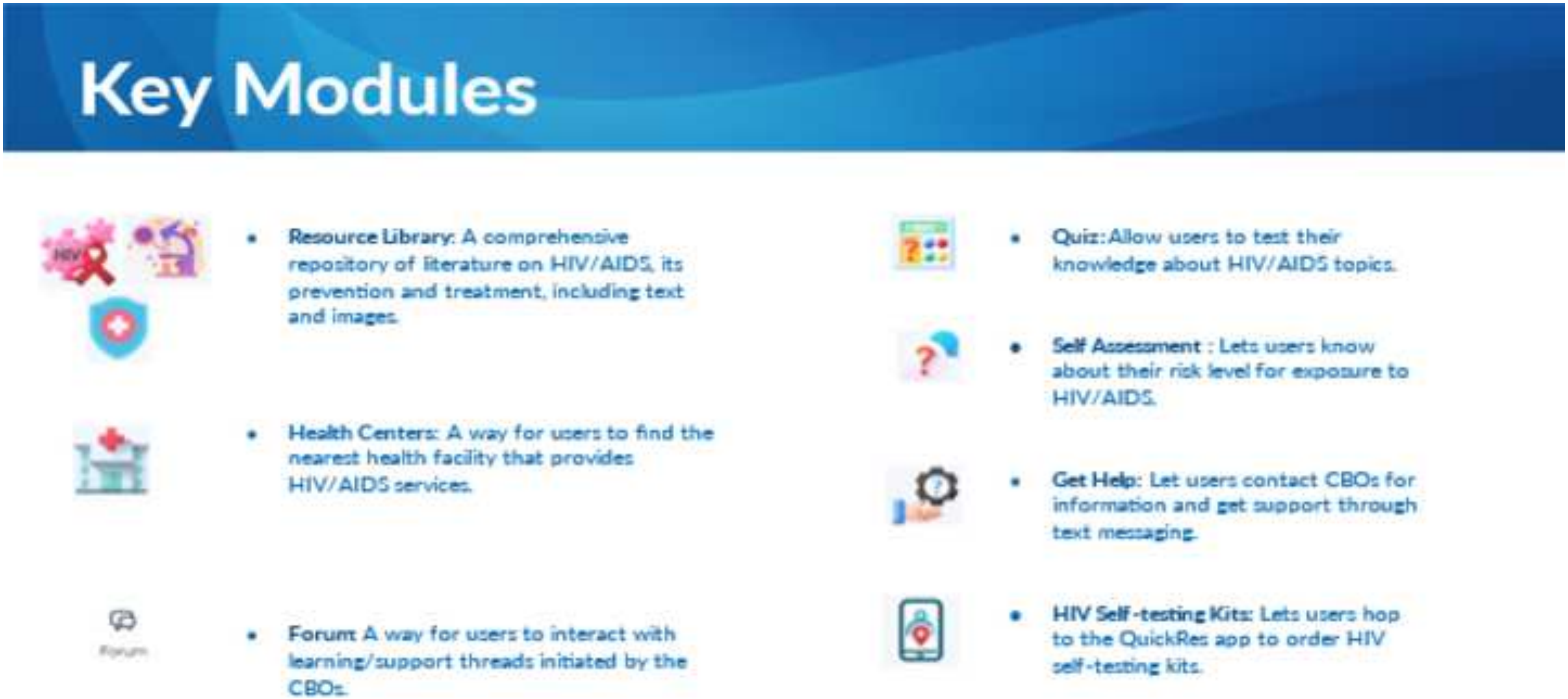

Click here to access/download
Supporting Information
IRB LETTER.pdf
