# Supplementary material for: Evaluating the digital dispensation of HIV self-testing (HIVST) among men who have sex with men (MSM) in Pakistan: Seeing through the Consolidated Framework for Implementation Research (CFIR) lens
Source: PLOS Glob Public Health. 2026 Sep 11;6(9):e0005994. doi: 10.1371/journal.pgph.0005994 (PMC13567783; doi:10.1371/journal.pgph.0005994)
Supplement: Supplementary file 2 [file pgph.0005994.s002.pdf]

Summary

This study qualitatively assesses a digital HIV self-testing (HIVST) intervention among men who have sex with men (MSM) in Pakistan, focusing on user experiences with an online platform that allows ordering of OraQuick self-test kits for home delivery. Using 12 in-depth interviews and two focus group discussions, the authors found that digital HIVST is generally acceptable and preferred because it reduces stigma, enhances confidentiality, and offers convenience compared to facility-based testing. Participants also noted the importance of peer sharing and digital platforms in potentially increasing HIV testing uptake within the MSM community. However, they identified key barriers, including concerns about confidentiality at home, ongoing stigma, challenges in navigating the platform, and fragmentation between different digital services. Participants further emphasized the need for a more comprehensive service package, including condoms, lubricants, and online counseling, as well as improved demand generation through social media and dating apps. Overall, the study indicates that while digital HIVST holds great potential in this context, optimizing platform design, service integration, and delivery strategies is necessary for effective scale-up.

Major Comments

1. The study does not clearly demonstrate whether data saturation was achieved, which is essential for qualitative rigor.
2. Sampling bias is a significant limitation, as participants were recruited through community-based organizations and are likely more engaged with HIV services than the broader MSM population.
3. The sample size is small and lacks diversity (e.g., mostly educated, urban participants), limiting generalizability.
4. The methodological description lacks detail, particularly regarding coding strategy, development of themes, and how discrepancies between coders were resolved.
5. The analysis is largely descriptive, with limited interpretive depth or engagement with relevant theoretical frameworks (e.g., stigma, digital health adoption).
6. There is conceptual overlap between themes (e.g., stigma, confidentiality, and acceptability), which weakens the clarity and structure of the findings.
7. The manuscript does not sufficiently explore contradictory perspectives (e.g., home testing as both private and potentially risky).
8. The discussion section lacks depth, particularly in translating findings into actionable policy or programmatic implications within the Pakistani context.
9. Ethical considerations are underdeveloped, given the criminalization and stigmatization of MSM, especially in relation to digital privacy and potential risks of home delivery.
10. The data availability statement ("available on request") may not meet journal requirements and needs clarification or revision.

Minor Comments

1. Several grammatical and language issues are present; the manuscript would benefit from professional editing for clarity and flow.
2. There is repetition of participant quotes and ideas, particularly around stigma and confidentiality.
3. Table II contains an error ("mean age… 24,500 PKR"), indicating incorrect units and poor formatting.
4. Some references are incorrectly formatted or incomplete, and links (e.g., UNAIDS citation) appear inaccurate.
5. The ethical statement is repeated, suggesting duplication that should be corrected.
6. Terminology such as MSM, MSW, and bisexual is noted as confusing by participants, but not clearly defined in the manuscript.
7. The relationship between the two digital platforms (Sehat Dost and QuickRes) is not clearly explained and may confuse readers.

8. Transitions between sections and themes are abrupt, affecting overall readability.
9. The manuscript would benefit from clearer subheadings in the discussion to improve structure.
10. Some sentences are awkwardly phrased or unclear, requiring refinement for precision and readability.
