# Supplementary material for: Evaluating the digital dispensation of HIV self-testing (HIVST) among men who have sex with men (MSM) in Pakistan: Seeing through the Consolidated Framework for Implementation Research (CFIR) lens
Source: PLOS Glob Public Health. 2026 Sep 11;6(9):e0005994. doi: 10.1371/journal.pgph.0005994 (PMC13567783; doi:10.1371/journal.pgph.0005994)
Supplement: Supplementary file 3 [file pgph.0005994.s005.pdf]

PLOS Global Public Health

Qualitative evaluation of the digital HIV self-testing (HIVST) intervention among men who have sex with men (MSM) in Pakistan.

--Manuscript Draft--

**Manuscript Number:**	PGPH-D-26-00385R2
**Article Type:**	Research Article
**Full Title:**	Qualitative evaluation of the digital HIV self-testing (HIVST) intervention among men who have sex with men (MSM) in Pakistan.
**Short Title:**	Qualitative evaluation of the digital HIV self-testing (HIVST) intervention in Pakistan.
**Corresponding Author:**	Usman Ali<br>King Edward Medical College: King Edward Medical University<br>Lahore, PAKISTAN
**Order of Authors:**	Usman Ali
	Safdar Kamal Pasha
	Ramesh Kumar
	Rab Nawaz Samo
	Raza Haider Tirmizi
	Siddique Wali
**Keywords:**	Good health and well-being; gender equality; reduced inequalities
**Abstract:**	HIV self-testing (HIVST) has recently been introduced through a digital platform for men who have sex with men (MSM) in Pakistan, where they can order and receive OraQuick HIVST via mail. The current study aimed to evaluate the digital dispensation of HIVST to the MSM community in Pakistan. Semi-structured interviews with MSM who availed HIVST services were conducted through in-depth interviews (IDIs) and focus group discussions (FGDs). Participants were recruited from HIV CBOs in Karachi and Lahore. The interviews were coded, and thematic analysis was performed using NVIVO 14.0. Twelve MSM were interviewed in the IDIs, and two FGDs were conducted with a total of 12 MSM. Key emergent themes highlighted that digital dispensation reduces HIV testing barriers and allows peer diffusion of testing services. In addition, the digital dispensation bypasses the stigma faced during in-person testing, including in the CBOs. However, MSM and HIV related stigma forces MSM to find innovative methods of HIVST delivery at home or a place of choice. The stigma in household settings also makes them vulnerable to enacted stigma in these settings. Participants noted difficulty in platform navigation, and a lack of comprehensive condoms, lubricants, and digital counselling services. The participants emphasized the importance of demand generation through social media and dating applications. Provision of a comprehensive service delivery package, digital demand generation through social media, and dating applications is recommended for strengthening digital HIVST services. The current banning of MSM dating sites in Pakistan poses a significant barrier to demand generation.
**Additional Information:**	
**Question**	**Response**
**Financial Disclosure**<br><br>Enter a financial disclosure statement that describes the sources of funding for the work included in this submission and the role the funder(s) played. This includes	The authors received no specific funding for this work.

Powered by Editorial Manager® and ProduXion Manager® from Aries Systems Corporation

grants and any commercial funding of the work or authors.<br><br>This statement will be typeset if the manuscript is accepted for publication.<br><br>*Please review the submission guidelines and the instructions link below for detailed requirements and guidance.*	
**Competing Interests**<br><br>On behalf of all authors, disclose any competing interests that could be perceived to bias this work.<br><br>This statement will be typeset if the manuscript is accepted for publication.<br><br>*Please review the instructions link below and PLOS Global Public Health's competing interests policy to determine what information must be disclosed at submission.*	The authors have declared that no competing interests exist.
**Data Availability**<br><br>Before publication, Authors are required to make fully available and without restriction all data underlying their findings. Sharing the data underlying your findings increases the visibility, credibility, and impact of your work, while supporting transparency and future scientific discovery. Please see our PLOS Data Policy page for detailed information on this policy.<br><br>A **Data Availability Statement**, detailing where the data can be accessed, is required at first submission. Insert your Data Availability Statement in the box below.	Data analyzed is in the manuscript. Interview transcripts can be available on reasonable request.

Powered by Editorial Manager® and ProduXion Manager® from Aries Systems Corporation

Please see the data reporting section of our submission guidelines for instructions on what you need to include in your Data Availability Statement. Examples of acceptable Data Availability Statements are included here for reference:<br>1. The minimal data set [and accompanying code, where generated] is available at [repository name] via [DOI hyperlink]<br>2. No data was generated by this study. The following existing data sources were used: [dataset name] from [dataset location] available via [web address or DOI hyperlink].<br>3. For Study Protocols: No datasets were generated or analysed during the current study. All relevant data from this study will be made available upon study completion.<br><br>*PLOS allows rare exemptions to address legal and ethical concerns. If you have legal or ethical restrictions, please use the box below to detail these in full sentences for the Journal team to consider.*<br><br>This statement will be typeset if the manuscript is accepted for publication.	

Powered by Editorial Manager® and ProduXion Manager® from Aries Systems Corporation

Click here to access/download;Manuscript;Revsied clean version 2.docx

Evaluating the digital HIV self-testing (HIVST) dispensation among men who have sex with men (MSM) in Pakistan: Seeing through the Consolidated Framework for Implementation Research (CFIR) lens.

Acknowledgements

We acknowledge XX and YY for their generous help with participant recruitment and data collection for this study.

Disclosure

The authors declare no potential conflicts of interest concerning the research, authorship, and/or publication of this article.

Funding

The authors received no financial support for the research, authorship, and/or publication of this article.

Abstract

HIV self-testing (HIVST) has recently been introduced through a digital platform for men who have sex with men (MSM) in Pakistan, where they can order and receive OraQuick HIVST via mail. The current study aimed to evaluate the digital dispensation of HIVST to the MSM community in Pakistan. Semi-structured interviews with MSM who availed HIVST services were conducted through in-depth interviews (IDIs) and focus group discussions (FGDs). Participants were recruited from HIV CBOs in Karachi and Lahore. The interviews were coded, and thematic analysis was performed using NVIVO 14.0. Twelve MSM were interviewed in the IDIs, and two FGDs were conducted with a total of 12 MSM. Key emergent themes highlighted that digital dispensation reduces HIV testing barriers and allows peer diffusion of testing services. In addition, the digital dispensation bypasses the stigma faced during in-person testing, including in the CBOs. However, MSM and HIV related stigma forces MSM to find innovative methods of HIVST delivery at home or a place of choice. The stigma in household settings also makes them vulnerable to enacted stigma in these settings. Participants noted difficulty in platform navigation, and a lack of comprehensive condoms, lubricants, and digital counselling services. The participants emphasized the importance of demand generation through social media and dating applications.

Provision of a comprehensive service delivery package, digital demand generation through social media, and dating applications is recommended for strengthening digital HIVST services. The current banning of MSM dating sites in Pakistan poses a significant barrier to demand generation.

Keywords:

Good health and well-being; gender equality; reduced inequalities

Introduction

Men who have sex with men (MSM) include gay and bisexual men, male sex workers, men who may not identify with these categories but engage in sexual activity with other men, and transgender women.[1] MSM are one of the five key populations at risk for HIV infection.[2] Globally, 7.6% of MSM live with HIV, which is ten times the global prevalence of HIV in the general population.[2] MSM are stigmatized and discriminated against in various jurisdictions. Around 63 countries have some law that prohibits same-sex relationships, with 12 countries proposing capital punishment for same-sex relationships.[2,3] Globally, 40.5% of MSM have reported facing stigma in the last 12 months.[4]

Pakistan is one of the 12 countries that proposes life imprisonment for same-sex relationships under Section 377 of the Pakistan Penal Code. [5] Criminalization is one aspect of manifested stigma against MSM. A recent study found that 10% of MSM living with HIV reported exclusion from family because of their MSM identity, 3 % reported being blackmailed, and 13% reported being physically harassed.[5] Thus, MSM face layered stigma stemming from religion, culture, social values, and the legal system. At the same time, MSM are the largest key population in the country, with a population estimate of around 1 million; around 5.4% of these are HIV positive. This is in contrast to a 0.1% prevalence in the general population. Thus, for every four people living with HIV in the country, 1 is MSM.[6] This highlights the health inequalities MSM face in Pakistan. [1]

Despite being at immense risk of HIV in the country, the coverage of HIV services for MSM is low, partly because of the hidden nature of the community. According to the Pakistan AIDS Strategy, only 9% of MSM had access to HIV prevention, and 6% had access to HIV testing in 2019. [6] This is in contrast to the United Nations Goal of 90-90-90 by 2020, which stated that

90% of people know their HIV status; that is, they have been tested and counseled on their HIV status.[7]

For a decade, HIV services had been solely provided by venue-based HIV testing either within the community-based organizations (CBOs) or through outreach at hotspots. [8] However, with increased digitalization, MSMs are increasingly using dating applications for partner cruising. In the Pre-exposure Proxphylaxis (PrEP) feasibility study conducted in 2020, MSM and transgender people reported that 65.7% used dating applications such as Grindr or Tinder. In addition, TikTok, Instagram, and Facebook were used by 34.7% of the participants for client cruising, while hotspot client cruising was also similar.[9] Thus, hotspot-based HIV service provision may pose serious limitations in reaching the MSM community. In addition, stigma and discrimination may deter MSM from engaging in venue-based testing services. HIV self-testing is a validated method of screening for HIV. People can self-sample and self-screen for HIVST with minimal guidance from a healthcare provider.[10] Leveraging the potential of digital outreach and HIVST. A digital HIV prevention and HIVST dispensation program is piloted in two major cities of Pakistan: Lahore and Karachi.

The current study aimed to evaluate the digitally facilitated HIV self-testing services for MSM in Pakistan.

Materials and Methods

This qualitative exploratory study was conducted from May 2025 to July 2025 in Lahore and Karachi.

Ethics Statement

Ethical approval for this study was obtained from the (No.XXX Fr. No. Dated XXXX2025)

Description of the Digital HIVST model

Two digital platforms were developed and linked: one providing information on HIV prevention, treatment, and facilities (Sehat Dost), and the other for ordering HIVST kits (QuickRes). The interface of Sehat Dost (www.sehatdost.com) has seven key modules. The last module facilitates digital HIVST dispensation. Once someone clicks on HIVST dispensation, it lands on a webpage of QuickRes (www.quickres.org). QuickRes is a global platform used to make appointments and

reservations for HIV services.[11] MSM can place an order for oral fluid-based HIVST on QuickRes. The order is placed, and the venue and time for collecting HIVST are selected; at the given time and venue, the CBO staff provides HIVST kits through a digital service or staff.

To generate demand for HIV services, advertisements are made in CBO drop-in centers (DIC) as well as messaging on WhatsApp, which includes a package of two videos, one focusing on how to perform HIVST and the other on how to use digital platforms. The demand generation faces significant challenges, as CBOs have to work in relative secrecy given the legal and social atmosphere of the country.

Participants

People above 18 years of age, male, identified as men who have sex with men, who have received both in-person and digital HIV testing services, were identified and recruited by the outreach workers of two CBOs: one based in Lahore (Dostana) and one based in Karachi (Humraz). CBO workers and those who had been diagnosed with HIV were excluded from the study. Both CBOs performed. Purposive sampling was conducted.

Interviews and the data collection process

Once eligible participants were recruited, they were given the option of being interviewed either individually (in-depth interview) or in a focus group discussion (FGD). Written informed consent was obtained before data collection. Semi-structured interviews were conducted face-to-face by a lead investigator using an interview guide. Interviews were conducted on CBO premises in a separate room, ensuring confidentiality. Interviews lasted from 25 minutes for IDIs to over an hour for FGDs. Remuneration was not provided to the participants. Participants were asked about their experience with digital platforms for HIVST, how it compares with in-person HIV testing at the CBO, perceived barriers, and benefits of digital HIVST services.

All interviews were conducted in Urdu, the national language of the country. Audio from interviews was recorded on a personal device. This was followed by manual transcription and English translation. Data collection and preliminary analysis were conducted concurrently. Saturation was considered achieved when no new codes or concepts emerged from successive interviews. Saturation was reached after 12 interviews, after which 2 additional interviews were conducted to confirm the stability of the findings.

To ensure confidentiality, the data were anonymized by assigning codes to individual participants, such as MSM1 and MSM2. Only the interview personnel had access to the anonymous data.

Data analysis

The transcripts were imported into NVIVO version 14.0.[12] Analysis was driven by an inductive thematic analysis followed by post-hoc interpretation using Consolidated Framework for Implementation Research (CFIR) as an analytic lens. CFIR is a key framework used in implementation science to evaluate complex interventions, including public health interventions.[13–15]

The data transcript was available to two independent researchers. After becoming familiar with the data, the researcher independently conducted description- and interpretation-focused coding. In the description-focused coding technique, the researcher assigns a code that captures the participant's experience without inference. In interpretation-focused coding, the researcher assigns codes to capture the underlying processes of the experience. Any discrepancy in coding was settled by mutual consensus and deliberation guided by a third researcher. After coding had been completed, codes were sorted into clusters using an individual-based sorting strategy. Codes were sorted into clusters based on similarities and differences. Final themes were arrived at to answer the research questions. For this study, the key research questions were: 1) Is the current digital HIVST an acceptable and useful intervention for the MSM community? 2) What are the opportunities to adapt digital HIVST services to make them more effective for improving HIV testing among MSM?

In the final step, themes were interpreted onto CFIR domains and constructs. Where appropriate, themes were mapped on more than one CFIR domain to reflect their complexity and cross-cutting nature.

The whole process was iterative, conducted through deliberations between the research team. The detailed methodology of coding and sorting was adapted by Adu et al. [16] The results were reported using Consolidated Criteria for Reporting Qualitative Studies (COREQ). [17]

Reflexivity Statement

The lead investigator has been working in HIV for nearly seven years, whereas XX has been working for nearly two decades. YY has been involved in community research and various qualitative projects, whereas XX has worked at the national level in HIV response with various agencies. The lead investigator is a community advocate. Throughout the research, reflexive discussions were undertaken to examine the impact of researchers' backgrounds and experiences on research. The practice of independent coding by two researchers, followed by iterative sorting, helped bracket individual perspectives of the researchers and enhance methodological rigor. Findings were supported by verbatim from the transcripts to ensure credibility and transparency.YY has worked in public health for over two decades, providing an overview of the data coding and analysis processes.

Results

Twelve IDIs were conducted in total. All participants had at least intermediate education (12 years of education). All except one were employed. All participants had their first HIVST (index) in a drop-in center (DIC). The summary characteristics of the participants interviewed through the IDI are given in the table below.

Table I. Characteristics of in-depth interviews, n=12.

Serial no.	Participant ID	Age in years	Education	Occupation	Income in PKR	Index HIVST
1.	MSM1	25	Bachelors	Lawyer	70000	DIC
2.	MSM2	23	Bachelors	Veterinary doctor	60,000	DIC
3.	MSM3	21	Bachelors	Freelance advertiser	100,000	DIC
4.	MSM4	21	Bachelors	Unemployed	0	DIC
5.	MSM5	22	Intermediate	Fashion designer	40,000	DIC
6.	MSM6	28	Bachelors	Paramedic	25000	DIC
7.	MSM7	23	Bachelors	Part-time employee	25,000	DIC
8.	MSM8	47	Masters	Artist	100,000	DIC

9.	MSM9	24	Bachelors	Advertising agency	140,000	DIC
10.	MSM10	25	Bachelors	Office boy	45,000	DIC
11.	MSM11	27	Masters	Display designer	100,000	DIC
12.	MSM12	28	Bachelors	Sex work/ office work	45,000	DIC

In addition, two focus group discussions were conducted with 12 MSM. The mean age of the participants was 25.85 years. All participants had at least matriculated. Three of the 12 participants were unemployed. The mean income of participants was 24,500 PKR. The summary characteristics of the participants interviewed through FGD are presented in the table below.

Table II. Characteristics of participants interviewed in the focus group discussion (FGD), n=12

Serial no.	Characteristics	Frequency (Percentage)
1.	Mean age of participants in years	25.85
2.	Education	
	No education	0 (0%)
	Under Matric	0 (0%)
	Matric to Intermediate	9 (75%)
	Graduation and above	3 (25%)
3.	Employment status	
	Employed	9 (75%)
	Unemployed	3 (25%)
4.	Mean income of participants in Pakistani Rupees	24,500

Based on thematic analysis of the qualitative interviews, three themes and eleven subthemes emerged. The table III below presents the thematic analysis mapping on CFIR domains and constructs.

Table III. Mapping of themes and sub-themes on CFIR domains and constructs.

Theme	Subtheme	CFIR domain	CFIR constructs
Digital dispensation reduces HIV testing barriers and allows peer diffusion to MSM not reached by CBOs	Digital HIVST dispensation provides better privacy and control over sexuality and HIV status disclosure	Innovation, Individuals	Innovation relative advantage, Individual recipient's capability
	HIVST reduces cost and time barriers to HIV testing	Innovation	Innovation relative advantage
	Peer-driven diffusion of HIV testing to MSM not accessible by the CBOs	Innovation, outer setting, Individuals	Innovation adaptability, Innovation recipient needs, and resources
	Digital guidance and a simple interface facilitate platform usability	Innovation, outer setting, Individuals	Innovation adaptability, Communications, Implementation facilitators
Digital platform users trade off between stigma at CBOs vs at home for HIV testing	Fear of enacted stigma while dispensing HIVST at home	Innovation, Outer setting	Local attitudes, Local conditions, Innovation relative advantage
	Lack of privacy at home	Innovation, Outer setting	Local attitudes, Innovation relative advantage
HIVST digital platform needs system maturation, demand generation, and integration in broader HIV services	Users face problems related to the design and structure of the digital platform	Innovation, Inner setting	Innovation design, Information technology infrastructure, Compatibility
	Users face problems related to the content of the platform	Innovation	Innovation design
	Users request the provision of comprehensive services through digital order	Individual	Innovation recipient need
	Using diverse demand generation through in-person interactions and digital means	Outer setting, Inner setting	Communications, Partnership, and connections

Theme 1. Digital dispensation reduces HIV testing barriers and allows peer diffusion to MSM not reached by CBOs

Based on participant interviews, it was highlighted that the intervention had been favored positively by the users at individual levels, as well as having an impact at the community level. The participants preferred HIVST dispensation using a digital platform as it allows them to keep their HIV status and sexuality confidential. Having a positive experience with digital HIVST dispensation allowed them to overcome time and cost barriers to CBO-based HIV testing. The participants agreed on user-friendly guidance to use the digital dispensation system. Given their positive experience, they engaged their peers and included them in the HIV testing network, allowing the intervention to reach MSM not reached by CBOs directly, through secondary distribution.

Subtheme 1.1. Digital HIVST dispensation provides better privacy and control over sexuality and HIV status disclosure

The participants compared digital HIVST dispensation with HIV testing in CBOs. The participants reported greater control, as they can now choose not to disclose their sexuality or HIV status to anyone. The perceived stigma from the community within CBOs is seen as a barrier. Other community members in CBOs may speculate about HIV test results based on behavior, while HIVST at home through digital dispensation bypasses this barrier, creating a favorable perception of digital HIVST compared to testing in CBOs. A participant reported that

Because at home, no one else is around in that moment. But if you're taking the test here at the center, your facial expressions and how you react can give something away. If you get tense or scared, people around you who are watching might notice. They'll realize you're doing an HIV self-test and might assume the result is bad. MSM7, 23 years old, part-time employee, 14 years of education

Though CBOs are a place for the MSM community, there are varying positions, such as paramedics or doctors who are not community members. Participant reported that through digital HIVST dispensation, they have more autonomy in terms of confidentiality.

When we come here, we have to first go to the office and give our ID. Then we visit the doctor; he's a paramedic, and he conducts the test. But at home, we do it ourselves. We already know how. In Punjabi, we say "don't open your mouth… meaning, just do it quietly and easily."FGD1

Community members have varying levels of comfort in disclosing their sexuality and varying levels of self-acceptance or self-identity within the MSM spectrum. Coming to CBO may have meaning that they have to identify with a particular identity for which they are not ready. The digital intervention also empowers participants to seek HIV testing services while still processing their identities.

Some people hesitate a lot while coming to the CBOs. They don't want to accept (their sexuality) for whatever reason they have. So, self-testing is very easy for them. They can test themselves at their place or anywhere. Some people have social anxiety; they don't want to face it. They don't know what will happen if they come here. MSM4, 21 years old, graduate, unemployed

These findings suggest that digital HIVST dispensation empowers the participants in terms of privacy and confidentiality, thus making it more acceptable.

Subtheme 1.2. HIVST reduces cost and time barriers to HIV testing

Even though HIV testing is free at the CBO, there are time and travel-related costs for availing of on-site HIV testing. Digital HIVST dispensation reduces these barriers, thus participants favor it compared to CBO-based HIV testing. One participant during the FGD stated that

I think it has a lot of benefits, because sometimes you don't have money to go to the CBO. They provide it free of cost, including delivery, which is very convenient. You get it for free. Many friends are busy during the day and don't have time. FGD 1

Another participant from the second FGD stated

Yes, I had done the test before. But because of my busy schedule with university and classes, it was a bit difficult to come here. So doing it at home was much easier for me. I ordered it through a link from a digital platform. .... I received the kit, and I did the test myself. I rubbed the swab on my gums and then dipped it, just like the instructions said. FGD2

These accounts emphasize how digital HIVST dispensation can be taken up by reducing financial and time-related barriers, without interfering with daily routine tasks.

Subtheme 1.3. Peer-driven diffusion of HIV testing to MSM not accessible by the CBOs

Having a favorable experience with the digital HIVST dispensation, there is peer-driven diffusion of HIV services to other MSM. This creates a mechanism of secondary dispensation, allowing an increase in testing coverage and network expansion. This can have a communal benefit.

Participants who were not healthcare staff of the CBO stated that they can now provide a link to services to other MSM who have not yet been reached by the CBOs, and would refrain from getting in touch with the CBO staff because of stigma, and maintain privacy. The potential of this digital intervention is recognized by one MSM, who stated that:

If we limit it to CBOs, then it will not reach everyone. MSM are those who don't believe in themselves. It is a good thing to reach them. MSM4, 21 years old, graduate, unemployed

Another participant recalled his experience of getting his friends tested through this service

I feel that the test done here is different from the one done at home…. When two of my friends visited me, I got their tests done at home. MSM5, 22 years old, fashion designer by profession, with 12 years of education

Another participant recalled a similar experience

I found out through an XX worker (at the CBO) that you can even get it (HIVST) delivered to your home. Then I passed that on to my friends I guided them too. I explained how to use the kit and how the process works. I showed them everything step by step. MSM6, 26 years old, paramedic by profession, 14 years of education

The accounts validate the diffusion of intervention, ensuring network expansion with users playing the role of network expanders.

Subtheme 1.4. Digital guidance and a simple interface facilitate platform usability

As mentioned above, the current structure of the intervention is the demand generation of HIVST by sharing instructions in the form of videos on how to operate and place an order on the digital platform, and a video on how to perform HIVST. These instructions, as well as the simple interface of the digital platform, are positively appraised by the community, enhancing confidence in the digital platform regarding its usability. During the FGD, the participant stated:

It is very good. I had no idea about the website. But the video has made it easy for us. We have sent it to many friends. It is very good. It has a lot of clarity about how to use the website in Urdu, what questions are there, and how to do it. It is very easy. It has all the to-the-point information. FGD1

Another participant highlighted that sending videos on WhatsApp made it easy to use the digital platform.

If you want to book a kit digital, then you have to go to QuickRes, right? Now, they also send videos. Like, XX (CBO worker), who sent me the video, he told me that if you want to tell someone further, then first send him a video so that he understands what it is, how to use it. So, sending a video is very beneficial. I also sent a link to him. There are some Facebook friends with whom I have never met, but there is a lot of talk on Facebook. MSM6, 26 years old, paramedic by profession, 14 years of education

The participants acknowledged that the use of the Urdu language made navigation easier on a digital platform.

No, it was very smooth. The website had a full Urdu language option. I didn't even know it could be done digitally until a friend told me. FGD2

However, these findings were not universal, and some gaps were highlighted by the participants, as given in Theme 3.

Theme 2. Digital platform users trade off between stigma at CBOs vs at home for HIV testing

As mentioned above, the digital dispensation of HIVST offers an acceptable private and confidential testing service; it does not eliminate stigma. Stigma from testing in CBOs is replaced by stigma in testing at home. This is compounded by increased scrutiny and a lack of privacy at home. The latter risks enacted stigma, because HIV is linked to sexual transmission and homosexuals, by default. To overcome these barriers, MSM, instead of getting HIV dispensed at home, may choose dispensation in the vicinity to avoid any risk of disclosure. Thus, MSMs weigh the benefits of testing at home as mentioned in the theme above, with potential risks highlighted in this section, choosing a personally suited strategy. However, digital HIVST dispensation provides them with an additional testing option to choose from in HIV services.

Subtheme 2.1. Fear of enacted stigma while dispensing HIVST at home

The participants reported not being out to family members as MSM, and risk exclusion from the family due to discrimination. One participant reported.

That fear is always there… if your MSM status is disclosed, your reputation (as a straight man) could be tarnished. You have your whole family whose prestige is at stake; it's not just about you. MSM8, 47 years old, artist by profession, 16 years of education

To navigate in this fearful environment, they choose to get HIVST dispensed other than in their homes, but in the vicinity

Many people do not give the address of their house, but give the nearby location of their house. For example, if there is a shop or a famous thing, if there is a shop a little ahead of their house, the rider goes there and calls them. FGD 1

Another participant in the FGD said that

The first thing that comes to mind is, where should I have it delivered? Because I can't have it sent to my home. So I try to make sure it's delivered somewhere far from home. Apart from that, I worry about how it will be. It's difficult for a newbie, but for a guide, it's not an issue. FGD2

For this reason, some MSMs prefer CBO testing, preferring a supportive environment over inadvertent disclosure of sexuality at home. One participant stated that

Here, the environment is friendly. It feels easier and more comfortable. At home, the family doesn't know about this. You have to keep the testing private from them. Naturally, we can't tell them. Some families are friendly, but in some households, you just can't share things like this. I don't think anyone would openly share something like this with their family. The good thing here is that there are friends around at the center, and the environment is friendly like home, but better. Still, if you think about it, going to and from the center takes time. At home, you can just take the test. MSM2, 23 years old, Veterinary doctor by profession

These fears may deter participants from getting HIVST dispensed at home, and instead, they choose a nearby place or prefer HIV testing in the CBO.

Subtheme 2.2. Lack of privacy at home

Linked closely with the fear of enacted stigma is the lack of privacy at home. This lack of privacy serves as the mechanism for the fear. The participants reported that hiding HIV testing kits at home is not possible, due to the lack of privacy and questioning in family settings. A participant stated that

I don't think anyone can hide it and keep it at home. If he uses a kit, they (family) will check it. He should not keep it in front of his family and face their questions. MSM2, 23 years old, Veterinary doctor by profession

When probed if a change of packaging without mention of HIV testing kits would solve this lack of privacy, a participant responded;

No matter what kind of packaging you use, if someone in the family even has the slightest doubt, they'll open it. Either that, or your reputation at home needs to be so solid that your privacy is respected. MSM8, 47 years old, artist by profession, 16 years of education

The lack of privacy is a concern because of the association with HIV-stigmatized communities. A participant reported that he could justify HIV kits at home because everyone is at risk, and it is not only contracted through sexual means; however, this participant did not challenge the lack of privacy at home either.

Even if you get it delivered to your home, there's no issue because this is common. It's not a gay disease or anything. It can happen randomly, even if you just go to a barber; their instruments can also be a source. This is a disease that can be contracted in many ways. So family members don't need to find out about it just because it's ordered at home. MSM9, 24 years old, working in an advertising agency, 14 years of education

The above excerpts from the interview focus on the prevalent generalized stigma against the HIV and MSM community in the households, triggering fear and posing barriers to HIV testing.

Theme 3. HIVST digital platform needs system maturation, demand generation, and integration in broader HIV services

Although the intervention was valued by the participants, several technical limitations were also highlighted, which may impact sustainability. In addition, the participants called for system integration with comprehensive HIV services. The finding reflects community readiness and the realization of a need for associated services. Participants also highlighted that for maximum impact, diverse digital demand generation is needed, leveraging social media and dating applications. However, digital interventions should not replace human interaction, both in demand generation at the community level but also individual counselling for service users.

Subtheme 3.1. Users face problems related to the design and structure of the digital platform

The current structure of having two platforms, one for HIV services and one for HIVST dispensation, was seen as complex by participants. The participants reported inconsistent linkages between the two, which make navigation difficult. Thus, optimal operation of the platforms requires a good level of digital literacy and comfort. However, given the literacy levels in MSM, who are marginalized, this is not optimal. These may limit the use of the platform to certain MSM rather than the MSM community as a whole. One participant stated that the platform is not designed for average MSM.

This platform isn't really for the average person; only someone who's a graduate or at least well-educated can manage to place an order. An uneducated or less literate person wouldn't be able to do it easily. Only graduates can do this. But there should also be an option to call. You should call and receive a call from the representative. You should tell him all the details, your address, and it should reach you. FGD2

Since HIVST can be ordered from Quickres, but HIV prevention information is on the Sehat Dost platform, it may be cumbersome for some participants, as one participant stated:

As far as I know, both things should be on the same platform....So, I will not need to visit other platforms. MSM6, 26 years old, paramedic by profession, 14 years of education

Another participant highlighted that even among the two platforms, the linkage is inadequate

No, there's no option for Sehat Dost in QuickRes. The QuickRes option is available on Sehat Dost, but not vice versa. FGD2

This lack of linkage between platforms causes people to miss content on the complementary platform.

Some people know about it, and some don't. But it depends on the chain. As I know, if someone consults me, I will guide them properly. MSM6, 26 years old, paramedic by profession, 14 years of education

Apart from having two platforms as part of the digital intervention, participants also reported some technical problems related to bookings. One participant stated the following:

There are time slots shown at the beginning; you're asked to select one for delivery. Different time slots appear, and you're supposed to choose one. But at first, they don't get selected. Then, when you move to the next page, it shows that the slots are available, but on the next screen, they aren't. The system just assigns a random time on its own sometimes at a time when you're not available or there is any problem, then… MSM1, 25 years old, lawyer by profession

These reflections emphasize the need for system maturation, comprehensive single platforms designed for those with low digital literacy for maximum utilization.

Subtheme 3.2. Users face problems related to the content of the platform

The current language and data collection at the digital platform reflect use of technical jargon related to sexual identities. This creates confusion and mistrust among users. In addition, long data collection creates fear of data security and privacy. The participants' emphasis on these points reflects that the current content is not user-centered. Speaking about the technical language used on the platform, one participant stated that

There are a few questions that confuse people, like MSW, MSM, or bisexual. When we explain that these terms are for the community and that there's nothing to be afraid of and that their data is safe, then they're more comfortable. Some people did ask me what MSW, MSM, or bisexual means. These are just written differently, and people often get confused. But it's not a problem once it's explained; they're fine. Yes, people are a little afraid at first. FGD2

Some participants also criticized the platform for asking for too much information before booking. The participants are concerned about digital privacy, as giving personal information on a digital platform has risks and triggers anxiety.

I saw their questions; there were quite a lot, maybe 2–3 pages worth. They were very detailed. MSM5, 22 years old, fashion designer by profession, with 12 years of education

Thus, the content needs to be centered around user-friendly language, which shall improve trust. In addition, the collection of personal information should be minimized to encourage service use.

Subtheme 3.3. Users request the provision of comprehensive services through digital order

Participants have recommended the provision of comprehensive HIV services digitally, reflecting the community's readiness for uptake of digital services. These services should not be limited to HIVST but include condoms and lubricants as well as digital counselling for HIV test-takers.

One participant stated that

Condoms or lubricants, I believe, should be made available.. MSM 2

Similarly, another participant emphasized the same idea that

And apart from that, there should be an option for condoms as well. For example, if someone wants to use condoms but doesn't feel comfortable asking for them openly, then they should be able to get them delivered discreetly to their home, too. That would also be very beneficial in terms of protection. MSM6, 26 years old, paramedic by profession, 14 years of education

Other participants recommended HIV digital counselling. One participant highlighted the need for the same as

It is a digital service, and they are providing a kit, so counselling should also be done as the kits are provided. There can be digital counselling, and it can also be done via video call. Now, obviously, it is the age of digital. If there can be digital classes and digital meetings. So why can't counselling be done digitally? MSM 2

These excerpts highlight that the digital intervention can have a scope of expansion to comprehensive services, given the community demand.

Subtheme 3.4. Using diverse demand generation through in-person interactions and digital means

The participants recommended the need for a multipronged digital demand generation. These include outreach at dating applications, as well as social media. However, digital demand generation should not replace human interaction. In fact, interpersonal interaction can complement digital demand generation, ensuring uptake of services. One participant stated that

We can't send it through just by sharing the link, as it is sensitive. You can only share it after a deep conversation. Only then will the next person understand it and be able to use it positively. You can also give advertisements on Grindr and other dating applications. FGD1

While some people have stressed the use of dating applications for advertising, others have stated the use of social media applications for the demand generation of HIV services and self-testing.

Not everyone uses Grindr. For example, I don't use Grindr. Most people use Instagram and Facebook (for hookups and dates). Even within the gay community, not everyone is on Grindr. MSM7, 23 years old, part-time employee, 14 years of education

One participant stated that there should be group sessions to complement digital messaging, and stated that people may take digital messaging for granted.

There should be more awareness sessions in the CBOs, too. They should hold regular sessions so people can learn. Because most people don't read everything on the app. They either skip through or just don't bother going into full detail. FGD2

These reflections from the participants highlight the need for multipronged and well-connected digital outreach and demand generation, leveraging social media and dating applications, with interpersonal interactions, individually or as a group, rather than just sharing a platform link.

Discussion

Framework for evaluating digital HIVST dispensation

The real-world effectiveness of interventions depends on several factors, including factors other than the characteristics of the intervention itself. [14,15]Various theories, models, and frameworks have been proposed to ascertain the effectiveness of interventions.[14,15] One of the most influential frameworks is CFIR, proposed by Damschroder et al. The framework includes five major domains for evaluating complex interventions: innovation, process, inner setting, outer setting, and individual characteristics of users, as well as facilitators and implementers.[13] Our evaluation highlights that each of these domains influences user experiences and contributes to intervention uptake.

Acceptability of HIVST intervention

Zhu et al. conducted a randomized controlled trial comparing HIVST delivery through digital dispensation and in-person dispensation. The study showed that the option of digital dispensation increased HIV testing two-fold. [18] In two separate RCTs that used Internet-based HIVST dispensation, it was noted that testing rates increased 1.47 to 1.77-fold. [19] However, there is significant heterogeneity observed in acceptance and preference for digital HIVST interventions. In a recent review, the acceptance of digital HIVST ranged from 64.55% to 99.0%. The preference

for digital HIVST interventions ranged as low as 4.6% to 99.6%.[20] In a meta-analysis pooling results of digital HIVST interventions from low- and middle-income countries, HIV testing uptake was similar between digital and non-digital interventions.[21]

The participants in our sample emphasize the convenience and resource efficiency of digital dispensation of HIVST, stating relative advantage over facility-based HIV testing. In a previous pilot intervention of HIVST dispensation through CBO workers, 96.6% of MSM reported HIVST convenient to use. In the study, 55.3% of MSM preferred outreach through social media.[22] The current evaluation highlights that HIVST itself and its digital dispensation are acceptable to the MSM community. However, there are several caveats to this acceptability and preference which are covered in sections below.

Digital HIVST dispensation, stigma and mental health support

MSMs are marginalized in many muslim-majority countries, with Pakistan being one of them.[3] This poses unique challenges in reaching out to the community with health and HIV interventions. Stigma is faced in family settings, legal frameworks, as well as internalized or perceived stigma within community settings[23]. In the current study, participants have described how stigma, even within community settings such as CBOs, serves as a barrier to HIV testing uptake. HIVST through digital means provides an additional testing option for the MSM community in that context. However, the stigma in CBO testing is replaced by fear of stigma in home settings, an interaction of inner and outer settings. Thus, digital dispensation of HIVST is influenced by stigma but in different contexts. The fear of enacted stigma is not unfounded, as MSM and people living with HIV (PLHIV) are highly stigmatized in the context of Pakistan. In the National HIV Stigma Index 2.0, 10% of MSM living with HIV reported that they were excluded from family settings in the last twelve months because of their identity as MSM.[5] Around 21.53% of MSM PLHIV also reported that they felt they were gossiped about and discriminated against by family in the last year because they are MSM. The stigma is not limited to same sex prefrences or behaviors. PLHIV are also excluded and discriminated against in family settings.[5] Around 5% of PLHIV report that they are excluded from family events in the last twelve months. Due to this stigma, people refrain from sharing their HIV status with their family.[5] In the National Stigma Index 2.0, 26.6% of MSM PLHIV reported they feel uncomfortable sharing their HIV status with those close to them due to stigma. Testing in such a stigmatizing environment thus brings anxiety, as reported by the

participants[5]. In a meta-synthesis of HIVST interventions among MSM in Asia, it was found that mental distress is in fact a barrier for HIVST uptake.[10] The participants from our study have, in fact, stressed the need for counselling services, though provided digitally for users of digital dispensation.

Comprehensive service provision

According to the World Health Organization, comprehensive HIV prevention services include HIV testing services, condoms, PrEP, and the management of sexually transmitted infections. [24] The digital platform in our intervention provided HIV informational care and HIVST kits. The participants, however, recommended comprehensive services that they can access at CBOs, such as condoms and lubricants. This reflects the participants' needs and readiness for uptake of services. Provision of these services shall also require ‘relational connections’ with law enforcement agencies. Condoms are not positively seen in society, and dispensation using riders may precipitate harassment from police.[25] Online prescription and dispensation of PrEP can also be explored. However, medical regulators' mechanisms for online assessment and prescription shall need to be assessed for implementation. In Malaysia, another muslim-majority context, researchers have evaluated a digital application that provided a comprehensive HIV prevention package, including HIVST and PrEP. [26] This study employed a pre-post design. Among 50 MSM using the application, 84% used HIVST, with 42% receiving dispensation more than once.[26]

Digital platform integration

While information on these is available on the Sehat Dost platform, participants highlighted that the presence of a separate platform for HIVST dispensation (Quickres) may be cumbersome to use. Although the current model of intervention relies on digital dispensation and mailing of HIVST, participants stated that they shared the link to the digital dispensation platform with other community members. It should be noted that this method may be used to engage MSM who cannot be reached by conventional outreach by CBOs. These themes illustrate the need for innovation and adaptability. In addition, the users also highlighted provider-centered language and data capture that need to be addressed. These recommendations are important, given that innovation is inherently diffusing to late adopters, who may be less motivated to use the service.[27] Providing client-centered and easy usability of the platform will improve user satisfaction and increase use of the platform.

Demand generation

In terms of demand generation, the participants in the current study emphasized digital means. This includes the use of social media applications, which may be used for various purposes but may include cruising clients or dates. These platforms include Facebook and Instagram. However, the participants also highlighted the use of digital dating applications exclusively by MSM, such as Grindr or Blued. While the profile and risk behaviors of MSM who use dating applications have not been studied in Pakistan, it is not known if their risk behaviors are higher than those of MSM who do not use dating applications. Researchers from the United States have shown that MSM using Grindr had higher rates of chlamydia or gonorrhea infection than those who did not use Grindr, higher rates of HIV infection, condomless anal intercourse, and a higher number of male sexual partners. However, the cohort was more likely to initiate HIV PrEP than non-users.[28] This highlights the systematic differences among MSM who use dating applications. Thus, the digital dispensation of HIVST can be an effective method for engaging this community by linking Quickres with dating applications for demand generation. However, it should also be noted that dating applications are banned in Pakistan by the government; thus, advertising on these applications may require unbanning of the applications. Despite being banned by the government, proxy web administrators use these applications.[29]

Peer-driven intervention diffusion

Most of the study participants were young, educated, and employed. Previous research has shown that young and socioeconomically advantaged individuals are adopters of health technology. In addition, all participants in IDIs had index HIVST in DIC, followed by ordering HIVST on a digital platform. This highlights the increasing confidence of the MSM community in the HIVST intervention. According to the social network model of diffusion of innovation, opinion leaders help uptake of innovation or intervention. Our study highlights that the MSM were first promoted for use of HIVST in the DIC, followed by ordering through the digital platform. The study participants also highlighted that they recommended digital HIVST intervention to their peers, who were previously out of network reach by the CBOs. Thus, the intervention was able to reach them through social networks. This can increase the testing coverage of HIV among MSM in Pakistan. The global targets for HIV by 2025 were that 95% of people living with HIV know their status.[2] In the last national HIV strategy 2021-2025, it was revealed that only 2% of MSM had

been tested for HIV in the last 12 months.[6] According to the National AIDS Control Program (www.cmu.gov.pk), 84,421 PLHIV know their HIV status out of 330,000 infected. This reflects that only 25% of PLHIV in the country are aware of their HIV status. The use of digital HIVST intervention can thus offer support in increasing testing coverage for the MSM community in the country.

Limitation of the study

The current study, however, has several limitations. First, it explored the qualitative experiences of the participants. The participants were interviewed by a researcher who was a former manager at the CBO; this may induce reporting and desirability bias, where participants only reported favorable themes. The participants were from two cities in Pakistan; thus, their experience may not be generalizable to all MSM residing in the country. Furthermore, we only used CFIR for post-hoc inductive analysis. CFIR is a comprehensive tool for appraisal and planning of intervention. Many of the CFIR domains are related to implementer, organizational, and donor perspectives. Our study, thus, uses CFIR as a lens for capturing user perspectives while not evaluating the intervention from the viewpoint of other stakeholders. Nonetheless, the study has several strengths allowing for greater understanding of how outer settings influence HIVST digital dispensation and how the innovation can be adapted for improved efficiency.

Conclusion

Digital HIVST dispensation is acceptable. However, participants noted difficulty in platform navigation. While digital dispensation empowers people to have greater control over HIV and sexuality disclosure, it also introduces vulnerability of disclosure and stigma in home settings. There is a community readiness to uptake a comprehensive package of HIV digitally, in addition to HIVST. The intervention also holds promise of network expansion of services driven by peers to MSM not reached by the CBOs. The participants recommend digital demand generation and interpersonal interaction at demand generation as well as the testing stage in the form of video counselling. The current banning of MSM dating sites poses a significant barrier to demand generation.

Authors contribution

References

[1] Integrated Biological and Behavioral Surveillance of HIV in Pakistan: Round 5 - 2016-17. 2017.

[2] AIDS, crisis and the power to transform: UNAIDS Global AIDS Update. 2025.

[3] Human Dignity Trust. Map of Jurisdictions that Criminalise LGBT People. Https://WwwHumandignitytrustOrg/ 2024.

[4] Hear us out: Community HIV measuring HIV-related stigma and discrimination. 2023.

[5] Usman Ali. National HIV Stigma Index. 2024.

[6] Pakistan AIDS Strategy IV 2021-2025. 2020.

[7] End inequalities, end AIDS. Global AIDS Strategy 2021-2026. 2020.

[8] Ali U, Fatima B, Azmat S, Khan MS, Chaudhary R, Mirzazadeh A. The Effect of Emotional Intelligence and Employment on the Psychological Well-Being of HIV Key Population Workers. Journal of the College of Physicians and Surgeons Pakistan 2025;2025:1294–300. https://doi.org/10.29271/jcpsp.2025.10.1294.

[9] Ali U, Pasha SK, Patel R, Schmidt HM, Altaf A. Assessment of Acceptability of Pre-exposure Prophylaxis for HIV Prevention Among Men Who Have Sex with Men and Transgender Community in Pakistan. Journal of the College of Physicians and Surgeons Pakistan 2023;33:983–9. https://doi.org/10.29271/jcpsp.2023.09.983.

[10] Pasha SK, Ali U. Facilitators, Barriers, and Recommendations for Overcoming Barriers to HIV Self-Testing (HIVST) Uptake Among Men Who Have Sex with Men and Transgender People in Asia: A Meta-Synthesis. J Coll Physicians Surg Pak 2025;35.

[11] EpiC. QuickRes Technical Guide; A Global Online Reservation and Case Management Tool for HIV Programs. 2020.

[12] Dhakal K. NVivo. Journal of the Medical Library Association 2022;110. https://doi.org/10.5195/jmla.2022.1271.

[13] Damschroder LJ, Reardon CM, Opra Widerquist MA, Lowery J. Conceptualizing outcomes for use with the Consolidated Framework for Implementation Research (CFIR): the CFIR Outcomes Addendum. Implementation Science 2022;17. https://doi.org/10.1186/s13012-021-01181-5.

[14] Longworth GR, Goh K, Agnello DM, Messiha K, Beeckman M, Zapata-Restrepo JR, et al. A review of implementation and evaluation frameworks for public health interventions to inform co-creation: a Health CASCADE study. Health Res Policy Syst 2024;22. https://doi.org/10.1186/s12961-024-01126-6.

[15] Damschroder LJ, Aron DC, Keith RE, Kirsh SR, Alexander JA, Lowery JC. Fostering implementation of health services research findings into practice: A consolidated framework for advancing implementation science. Implementation Science 2009;4. https://doi.org/10.1186/1748-5908-4-50.

[16] Adu, Philip. A Step-by-Step Guide to Qualitative Data Coding. 2019.

[17] Tong A, Sainsbury P, Craig J. Consolidated criteria for reporting qualitative research (COREQ): a 32-item checklist for interviews and focus groups. International Journal for Quality in Health Care 2007;19:349–57. https://doi.org/10.1093/intqhc/mzm042.

[18] Zhu X, Zhang W, Operario D, Zhao Y, Shi A, Zhang Z, et al. Effects of a Mobile Health Intervention to Promote HIV Self-testing with MSM in China: A Randomized Controlled Trial. AIDS Behav 2019;23:3129–39. https://doi.org/10.1007/s10461-019-02452-5.

[19] Young SD, Cumberland WG, Singh P, Coates T. A Peer-Led Online Community to Increase HIV Self-Testing Among African American and Latinx MSM: A Randomized Controlled Trial. J Acquir Immune Defic Syndr (1988) 2022;90:20–6. https://doi.org/10.1097/QAI.0000000000002919.

[20] Beecroft A, Vaikla O, Engel N, Duchaine T, Liang C, Pant Pai N. Evidence on Digital HIV Self-Testing From Accuracy to Impact: Updated Systematic Review. J Med Internet Res 2025;27:e63110. https://doi.org/10.2196/63110.

[21] Nicely P, Xiang L, Smith-Sreen J, Garbern SC, Aluisio AR. Approaches and impacts of digital health in HIV self-testing uptake & use among populations from low- and middle-income countries: a descriptive systematic review and meta-analysis. J Public Health Emerg 2025;9:11–11. https://doi.org/10.21037/jphe-24-56.

[22] Altaf A, Pasha MSK, Wali MS, Majeed A. Acceptability and feasibility of intra-peer and social media approach for HIV self-testing among men who have sex with men in Karachi: lessons from a pilot study. J Pak Med Assoc 2023;73. https://doi.org/10.47391/JPMA.6846.

[23] Batchelder AW, Foley JD, Kim J, Thiim A, Kelly J, Mayer K, et al. Intersecting internalized stigmas and HIV self-care among men who have sex with men and who use substances. Soc Sci Med 2021;275. https://doi.org/10.1016/j.socscimed.2021.113824.

[24] Consolidated guidelines on HIV, viral hepatitis and STI prevention, diagnosis, treatment and care for key populations. 2022.

[25] Bano S, Rahat R, Fischer F. Inconsistent condom use for prevention of HIV/STIs among street-based transgender sex workers in Lahore, Pakistan: socio-ecological analysis based on a qualitative study. BMC Public Health 2023;23:635. https://doi.org/10.1186/s12889-023-15550-w.

[26] Shrestha R, Altice FL, Khati A, Azwa I, Gautam K, Gupta S, et al. Clinic-Integrated Smartphone App (JomPrEP) to Improve Uptake of HIV Testing and Pre-exposure Prophylaxis Among Men Who Have Sex With Men in Malaysia: Mixed Methods Evaluation of Usability and Acceptability. JMIR Mhealth Uhealth 2023;11:e44468. https://doi.org/10.2196/44468.

[27] Van Houtven CH, Drake C, Malo TL, Decosimo K, Tucker M, Sullivan C, et al. Ready, set, go! The role of organizational readiness to predict adoption of a family caregiver training program using the Rogers' diffusion of innovation theory. Implement Sci Commun 2023;4:69. https://doi.org/10.1186/s43058-023-00447-x.

[28] Hoenigl M, Little SJ, Grelotti D, Skaathun B, Wagner GA, Weibel N, et al. Grindr Users Take More Risks, but Are More Open to Human Immunodeficiency Virus (HIV) Pre-exposure Prophylaxis: Could This Dating App Provide a Platform for HIV Prevention Outreach? Clinical Infectious Diseases 2020;71:e135–40. https://doi.org/10.1093/cid/ciz1093.

[29] Gibran Naiyyar Peshimam. Pakistan blocks five dating apps including Tinder and Grindr. Reuters 2020.

Click here to access/download
Supporting Information
IRB LETTER.pdf

~~Qualitative evaluation of~~Evaluating the digital HIV self-testing (HIVST) ~~intervention~~dispensation among men who have sex with men (MSM) in Pakistan: Seeing through the Consolidated Framework for Implementation Research (CFIR) lens.

Formatted: Left, Line spacing: Multiple 1.08 li

Acknowledgements

We acknowledge XX and YY for their generous help ~~in~~with participant recruitment and ~~in facilitating~~ data collection for this study.

Disclosure

The authors~~(s)~~ declare no potential conflicts of interest concerning the research, authorship, and/or publication of this article.

Funding

The authors ~~(s)~~ received no financial support for the research, authorship, and/or publication of this article.

~~**Ethical statement**~~

~~**Ethics Statement**~~

~~Ethical approval for this study was obtained from the XXX (No. Dated 8 Jan 2025)~~

Abstract

HIV self-testing (HIVST) has recently been introduced through ~~an online~~a digital platform for men who have sex with men (MSM) in Pakistan, where they can order and receive OraQuick HIVST via mail. The current study aimed to ~~explore~~evaluate the ~~experiences~~digital dispensation of HIVST to the MSM community ~~who utilize HIVST services through online platforms~~in Pakistan. Semi-structured interviews with MSM who availed HIVST services were conducted through in-depth interviews (~~IDI~~IDIs) and focus group discussions (FGDs). Participants were recruited from HIV ~~community based organizations~~CBOs in Karachi and Lahore. The interviews were coded, and thematic analysis was performed using NVIVO 14.0. Twelve MSM were interviewed in the ~~IDI~~IDIs, and two FGDs were conducted with a total of 12 MSM. Key emergent themes highlighted ~~online ordering as a preferable method for~~that digital dispensation reduces HIV testing~~, as it~~

barriers and allows peer diffusion of testing services. In addition, the digital dispensation bypasses the stigma faced during in-person testing, ensures confidentiality, and provides convenience. Online HIVST ordering is acceptable given regular client support by a community-based organization (including in the CBOs) with the potential to increase HIV testing coverage through peer-to-peer sharing of an online ordering platform.. However, participantsMSM and HIV related stigma forces MSM to find innovative methods of HIVST delivery at home or a place of choice. The stigma in household settings also makes them vulnerable to enacted stigma in these settings. Participants noted difficulty in platform navigation, confidentiality, and stigma issues by ordering HIVST at home, and a lack of comprehensive condoms, lubricants, and onlinedigital counselling services. The participants also emphasized the importance of demand generation through social media and dating applications. Provision of a comprehensive service delivery package, digital demand generation through social media, and dating applications is recommended for strengthening online HIVST services. digital HIVST services. The current banning of MSM dating sites in Pakistan poses a significant barrier to demand generation.

Keywords:

Good health and well-being; gender equality; reduced inequalities

Introduction

Men who have sex with men (MSM) include gay and bisexual men, male sex workers, men who may not identify with these categories but engage in sexual activity with other men, and transgender women.[1] MSM are one of the five key populations at risk for HIV infection.(AIDS, Crisis and the Power to Transform: UNAIDS Global AIDS Update, 2025)[2] Globally, 7.6% of MSM live with HIV, which is ten times the global prevalence of HIV in the general population.(AIDS, Crisis and the Power to Transform: UNAIDS Global AIDS Update, 2025)[2] MSM are stigmatized and discriminated against in various jurisdictions. Around 63 countries have some law that prohibits same-sex relationships, with 12 countries proposing capital punishment for same-sex relationships.(AIDS, Crisis and the Power to Transform: UNAIDS Global AIDS Update, 2025; Human Dignity Trust, 2024)[2,3] Globally, 40.5% of MSM have reported facing stigma in the last 12 months.(Hear Us out: Community HIV Measuring HIV-Related Stigma and Discrimination, 2023)[4].

Formatted: Font color: Black

Pakistan is ~~also~~ one of the 12 countries that ~~proposed capital punishment~~proposes life imprisonment for ~~MSM~~same-sex relationships under Section 377 of the Pakistan Penal Code.~~(Usman Ali, 2024)~~ [5] Criminalization is one aspect of manifested stigma against MSM. A recent study found that 10% of MSM living with HIV reported exclusion from family because of their MSM identity, 3 % reported being blackmailed, and 13% reported being physically harassed.[5] Thus, MSM face layered stigma stemming from religion, culture, social values, and the legal system. At the same time, MSM are ~~a marginalized~~the largest key population ~~at risk of HIV infection. It is estimated that of the PLHIV pool~~ in the country, ~~25%~~with a population estimate of around 1 million; around 5.4% of these are ~~identified as MSM.(Pakistan AIDS Strategy IV 2021-2025, 2020) The~~HIV positive. This is in contrast to a 0.1% prevalence ~~of HIV infection among MSM in the country is 5.4%.(Integrated Biological and Behavioral Surveillance of HIV in Pakistan: Round 5 – 2016-17, 2017) It should be noted that the prevalence of HIV in the~~ in the general population. Thus, for every four people living with HIV in the country, 1 is MSM.[6] This highlights the health inequalities MSM face in Pakistan. [1]

Formatted: Font color: Black

Formatted: Font color: Black

Formatted: Font color: Black

Formatted: Font color: Black

Formatted: Font color: Black

Despite being at immense risk of HIV in the country, the coverage ~~is 0.1%.(Integrated Biological and Behavioral Surveillance~~ of HIV ~~in Pakistan: Round 5 – 2016-17, 2017) Despite the high prevalence of HIV, extending HIV prevention~~ services ~~to~~for MSM is ~~difficult~~low, partly because of ~~social stigma and their marginalized status~~the hidden nature of the community. According to the Pakistan AIDS Strategy, only 9% of MSM had access to HIV prevention, and 6% had access to HIV testing in 2019.~~(Pakistan AIDS Strategy IV 2021-2025, 2020)~~ [6] This is in contrast to the United Nations Goal of 90-90-90 by 2020, which stated that 90% of people know their HIV status; that is, they have been tested and counseled on their HIV status.~~(End Inequalities, End AIDS. Global AIDS Strategy 2021-2026, 2020)~~[7]

Formatted: Font color: Black

Formatted: Font color: Black

Formatted: Font color: Black

Formatted: Font color: Black

~~To curtail the HIV epidemic in MSM, Pakistan's National AIDS Strategy has focused on establishing community-based organizations (CBOs) for MSM that provide in-person HIV prevention and testing services.(Ali & Ali, 2023; End Inequalities, End AIDS. Global AIDS Strategy 2021-2026, 2020) However, in recent years, there has been an increasing shift in the MSM community to digital spaces to find sexual partners.(Ali et al., 2023) This includes dating applications and social media platforms. In a feasibility study on HIV pre-exposure prophylaxis (PrEP), 65.7% of MSM cruised partners through dating applications, whereas 34.7% used social~~

media applications.(Ali et al., 2023) As a result of this increasing use of digital spaces by MSM to socialize, the HIV prevention program aimed to launch a digital intervention for the MSM community in two major cities: Lahore and Karachi. Two online platforms were developed, with one providing information on HIV prevention, treatment, and facilities, called the Sehat Dost application (Figure 1; www.sehatdost.pk). Sehat Dost was linked to QuickRes (www.quickres.org). Quickres is a global platform used to make appointments and reservations for HIV services.(EpiC, 2020) The services provided in Pakistan through the platform include delivery of oral fluid-based HIV self-testing (HIVST). After an appointment for HIVST is booked, oral fluid-based HIVST (OraQuick) is delivered to the community in Lahore and Karachi. To generate demand for HIV services, advertisements are made in CBO drop-in centers (DIC) as well as messaging on WhatsApp, which includes a package of two videos, one focusing on how to perform HIVST and the other on how to use online platforms.

For a decade, HIV services had been solely provided by venue-based HIV testing either within the community-based organizations (CBOs) or through outreach at hotspots. [8] However, with increased digitalization, MSMs are increasingly using dating applications for partner cruising. In the Pre-exposure Proxphylaxis (PrEP) feasibility study conducted in 2020, MSM and transgender people reported that 65.7% used dating applications such as Grindr or Tinder. In addition, TikTok, Instagram, and Facebook were used by 34.7% of the participants for client cruising, while hotspot client cruising was also similar.[9] Thus, hotspot-based HIV service provision may pose serious limitations in reaching the MSM community. In addition, stigma and discrimination may deter MSM from engaging in venue-based testing services. HIV self-testing is a validated method of screening for HIV. People can self-sample and self-screen for HIVST with minimal guidance from a healthcare provider.[10] Leveraging the potential of digital outreach and HIVST. A digital HIV prevention and HIVST dispensation program is piloted in two major cities of Pakistan: Lahore and Karachi.

The current study aimed to exploreevaluate the experiences of the MSM community who utilizedigitally facilitated HIV self-testing services through these online platformsfor MSM in Pakistan.

Figure 1. Key modules on the Sehat Dost platform.

Formatted: Font color: Text 1

Formatted: Font color: Text 1

Formatted: Font color: Text 1

Formatted: Font color: Text 1

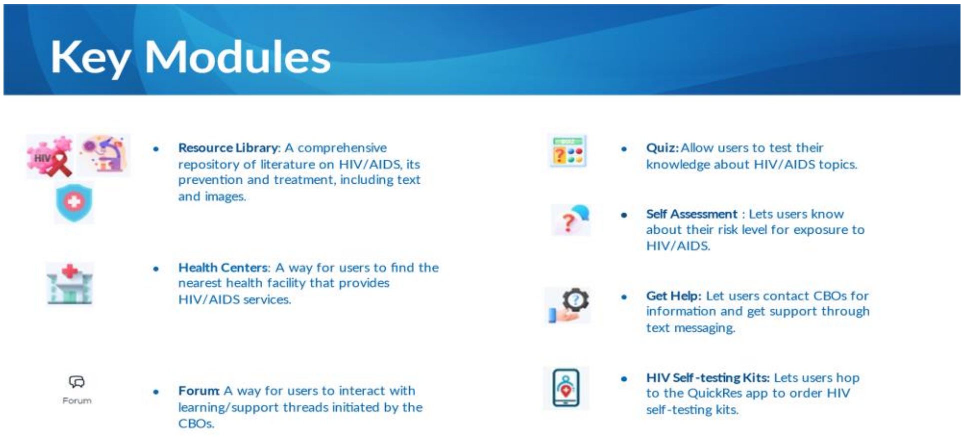

Materials and Methods

This qualitative exploratory study was conducted from May 2025 to July 2025 in Lahore and Karachi.

Ethics Statement

Ethical approval for this study was obtained from the ~~Health Services Academy of Islamabad~~ (No.XXX Fr. No. ~~000 I 7/l ISA/PhD-20231~~ Dated ~~8 Jan 2025~~XXXX2025)

Description of the Digital HIVST model

Two digital platforms were developed and linked: one providing information on HIV prevention, treatment, and facilities (Sehat Dost), and the other for ordering HIVST kits (QuickRes). The interface of Sehat Dost (www.sehatdost.com) has seven key modules. The last module facilitates digital HIVST dispensation. Once someone clicks on HIVST dispensation, it lands on a webpage of QuickRes (www.quickres.org). QuickRes is a global platform used to make appointments and reservations for HIV services.[11] MSM can place an order for oral fluid-based HIVST on QuickRes. The order is placed, and the venue and time for collecting HIVST are selected; at the given time and venue, the CBO staff provides HIVST kits through a digital service or staff.

To generate demand for HIV services, advertisements are made in CBO drop-in centers (DIC) as well as messaging on WhatsApp, which includes a package of two videos, one focusing on how to perform HIVST and the other on how to use digital platforms. The demand generation faces significant challenges, as CBOs have to work in relative secrecy given the legal and social atmosphere of the country.

Participants

Formatted: Font: Not Bold

People above 18 years of age, male, identified as men who have sex with men, who have received both in-person and onlinedigital HIV testing services, were identified and recruited by the outreach workers of two CBOs: one based in Lahore (Dostana) and one based in Karachi (Humraz). CBO workers and those who had been diagnosed with HIV were excluded from the study. Both CBOs provided services only to the MSM community. Convenient non-probabilityperformed. Purposive sampling was performed. Informed written consent was takenconducted.

Interviews and the data collection process

Once eligible participants were recruited, they were given the option of being interviewed in individual either individually (in-depth interviews (IDIinterview) or in a focus group discussionsdiscussion (FGD). InformedWritten informed consent was obtained, and the participants were interviewed in person before data collection. Semi-structured interviews were conducted face-to-face by a lead investigator using an interview guide. Semi-structured interviews were also conductedInterviews were conducted on CBO premises in a separate room, ensuring confidentiality. Interviews lasted from 25 minutes for IDIs to over an hour for FGDs. Remuneration was not provided to the participants. Participants were asked about their experience with digital platforms for HIVST, how it compares with in-person HIV testing at the CBO, perceived barriers, and benefits of onlinedigital HIVST. The services.

Formatted: Font color: Text 1

Formatted: Font color: Text 1

Formatted: Font color: Text 1

All interviews were conducted in Urdu, the national language of the country. Audio from interviews was recorded on a personal device,. This was followed by manual transcription and English translation. Data collection and preliminary analysis were conducted concurrently. Saturation was considered achieved when no new codes or concepts emerged from successive interviews. Saturation was reached after 12 interviews, after which 2 additional interviews were conducted to confirm the stability of the findings.

To ensure confidentiality, the data were anonymized by assigning codes to individual participants, such as MSM1 and MSM2. Only the interview personnel had access to the anonymous data.

Data analysis

The transcripts were imported into NVIVO version 14.0.~~(Dhakal, 2022)~~[12] Analysis was driven by an inductive thematic analysis followed by post-hoc interpretation using Consolidated Framework for Implementation Research (CFIR) as an analytic lens. CFIR is a key framework used in implementation science to evaluate complex interventions, including public health interventions.[13–15]

The data ~~were coded using descriptive~~transcript was available to two independent researchers. After becoming familiar with the data, the researcher independently conducted description- and interpretation-focused coding. ~~Key emergent~~In the description-focused coding technique, the researcher assigns a code that captures the participant's experience without inference. In interpretation-focused coding, the researcher assigns codes to capture the underlying processes of the experience. Any discrepancy in coding was settled by mutual consensus and deliberation guided by a third researcher. After coding had been completed, codes were sorted into clusters using an individual-based sorting strategy. Codes were sorted into clusters based on similarities and differences. Final themes were ~~identified in an iterative~~arrived at to answer the research questions. For this study, the key research questions were: 1) Is the current digital HIVST an acceptable and useful intervention for the MSM community? 2) What are the opportunities to adapt digital HIVST services to make them more effective for improving HIV testing among MSM?

In the final step, themes were interpreted onto CFIR domains and constructs. Where appropriate, themes were mapped on more than one CFIR domain to reflect their complexity and cross-cutting nature.

The whole process was iterative, conducted through ~~discussion~~deliberations between the ~~two authors (XX~~research team. The detailed methodology of coding and ~~TT). Thematic analysis~~sorting was ~~performed as described~~adapted by Adu et al~~, with consensus among both authors followed by review by YY.(Adu & Philip, 2019)~~. [16] The results were reported using Consolidated Criteria for Reporting Qualitative Studies (COREQ).~~(Tong et al., 2007)~~ [17]

Reflexivity Statement

Formatted: Font color: Text 1

Formatted: Font color: Black

Formatted: Font color: Black

Formatted: Font color: Black

Formatted: Font color: Black

Formatted: Font color: Black

Formatted: Font color: Black

Formatted: Font color: Black

Formatted: Font color: Black

The lead investigator has been working in HIV for nearly seven years, whereas XX has been working for nearly two decades. ~~TT~~YY has been involved in community research and various qualitative projects, ~~where~~whereas XX has worked at the national level in HIV response with various agencies. ~~Both coded~~The lead investigator is a community advocate. Throughout the ~~data after mutual~~research, reflexive discussions~~.~~ were undertaken to examine the impact of researchers' backgrounds and experiences on research. The practice of independent coding by two researchers, followed by iterative sorting, helped bracket individual perspectives of the researchers and enhance methodological rigor. Findings were supported by verbatim from the transcripts to ensure credibility and transparency.YY has worked in public health for over two decades, providing an overview of the data coding and analysis processes.

Results

Twelve ~~in-depth interviews (IDI)~~IDIs were conducted in total. All participants had at least intermediate education (12 years of education). All except one were employed. All participants had their first HIVST (index) in a drop-in center (DIC). The summary characteristics of the participants interviewed through the IDI are given in the table below.

Table I. Characteristics of in-depth interviews, n=12.

**Serial no.**	**Participant ID**	**Age in years**	**Education**	**Occupation**	**Income in PKR**	**Index HIVST**
1.	MSM1	25	Bachelors	Lawyer	70000	DIC
2.	MSM2	23	Bachelors	Veterinary doctor	60,000	DIC
3.	MSM3	21	Bachelors	Freelance advertiser	100,000	DIC
4.	MSM4	21	Bachelors	Unemployed	0	DIC
5.	MSM5	22	Intermediate	Fashion designer	40,000	DIC
6.	MSM6	28	Bachelors	Paramedic	25000	DIC
7.	MSM7	23	Bachelors	Part-time employee	25,000	DIC
8.	MSM8	47	Masters	Artist	100,000	DIC

Formatted: Font color: Text 1

Formatted: Font: Times New Roman, 12 pt, Not Bold

Formatted: Font: Times New Roman, 12 pt

Formatted: Font: Not Bold

Formatted: Heading 1, Left, Line spacing: single

Formatted Table

9.	MSM9	24	Bachelors	Advertising agency	140,000	DIC
10.	MSM10	25	Bachelors	Office boy	45,000	DIC
11.	MSM11	27	Masters	Display designer	100,000	DIC
12.	MSM12	28	Bachelors	Sex work/ office work	45,000	DIC

In addition, two focus group discussions were conducted with 12 MSM. The mean age of the participants was 25.85 years. All participants had at least matriculated. Three of the 12 participants were unemployed. The mean ~~age~~income of participants was 24,500 PKR. The summary characteristics of the participants interviewed through FGD are presented in the table below.

Table II. Characteristics of participants interviewed in the focus group discussion (FGD), n=12

Serial no.	Characteristics	Frequency (Percentage)
1.	Mean age of participants in years	25.85
2.	Education	
	No education	0 (0%)
	Under Matric	0 (0%)
	Matric to Intermediate	9 (75%)
	Graduation and above	3 (25%)
3.	Employment status	
	Employed	9 (75%)
	Unemployed	3 (25%)
4.	Mean income of participants in Pakistani Rupees	24,500

Based on ~~the~~ thematic analysis of the qualitative interviews, ~~five~~three themes ~~emerged, along with~~ and eleven subthemes emerged. The table III below presents the thematic analysis mapping on CFIR domains and constructs.

~~**Theme 1. Benefits of HIVST delivery to community individuals**~~

Formatted: Font: Times New Roman, 12 pt, Not Bold

Formatted: Heading 1, Left, Line spacing: single, Tab stops: Not at 1.24"

Formatted: Font: Not Bold

Formatted Table

Formatted: Font color: Black

This theme covers the self-perceived benefits of the intervention. This theme includes three subthemes. Overall, the theme reflects reasons for preferring the online ordering of HIVST in comparison with HIVST in the CBO.

Subtheme 1.1. Prefer online ordering of HIVST to avoid the stigma faced in the CBO

One self-perceived benefit of online ordering of HIVST is that it helps avoid the stigma faced by the CBO. Participants reported that they felt stigmatized by getting HIVST in the CBO drop-in center. One participant stated his fears about running into someone whom they knew in their personal life, but not out to them as MSM.

…because a lot of people still don't understand how to respond sensitively to this kind of thing (HIV). So there's fear. There is no issue coming here (CBO), but home is better. No fear of running into someone, no awkward eye contact.

Table III. Mapping of themes and sub-themes on CFIR domains and constructs.

**Theme**	**Subtheme**	**CFIR domain**	**CFIR constructs**
Digital dispensation reduces HIV testing barriers and allows peer diffusion to MSM not reached by CBOs	Digital HIVST dispensation provides better privacy and control over sexuality and HIV status disclosure	Innovation, Individuals	Innovation relative advantage, Individual recipient's capability
	HIVST reduces cost and time barriers to HIV testing	Innovation	Innovation relative advantage
	Peer-driven diffusion of HIV testing to MSM not accessible by the CBOs	Innovation, outer setting, Individuals	Innovation adaptability, Innovation recipient needs, and resources
	Digital guidance and a simple interface facilitate platform usability	Innovation, outer setting, Individuals	Innovation adaptability, Communications, Implementation facilitators
Digital platform users trade off between stigma at CBOs vs at home for HIV testing	Fear of enacted stigma while dispensing HIVST at home	Innovation, Outer setting	Local attitudes, Local conditions, Innovation relative advantage
	Lack of privacy at home	Innovation, Outer setting	Local attitudes, Innovation relative advantage
HIVST digital platform needs system maturation, demand generation, and integration in broader HIV services	Users face problems related to the design and structure of the digital platform	Innovation, Inner setting	Innovation design, Information technology infrastructure, Compatibility
	Users face problems related to the content of the platform	Innovation	Innovation design
	Users request the provision of comprehensive services through digital order	Individual	Innovation recipient need
	Using diverse demand generation through in-person interactions and digital means	Outer setting, Inner setting	Communications, Partnership, and connections

Theme 1. Digital dispensation reduces HIV testing barriers and allows peer diffusion to MSM not reached by CBOs

Based on participant interviews, it was highlighted that the intervention had been favored positively by the users at individual levels, as well as having an impact at the community level. The participants preferred HIVST dispensation using a digital platform as it allows them to keep their HIV status and sexuality confidential. Having a positive experience with digital HIVST dispensation allowed them to overcome time and cost barriers to CBO-based HIV testing. The participants agreed on user-friendly guidance to use the digital dispensation system. Given their positive experience, they engaged their peers and included them in the HIV testing network, allowing the intervention to reach MSM not reached by CBOs directly, through secondary distribution.

Subtheme 1.1. Digital HIVST dispensation provides better privacy and control over sexuality and HIV status disclosure

The participants compared digital HIVST dispensation with HIV testing in CBOs. The participants reported greater control, as they can now choose not to disclose their sexuality or HIV status to anyone. The perceived stigma from the community within CBOs is seen as a barrier. Other community members in CBOs may speculate about HIV test results based on behavior, while HIVST at home through digital dispensation bypasses this barrier, creating a favorable perception of digital HIVST compared to testing in CBOs. A participant reported that

~~*FGD 1*~~

~~Yet another MSM stated that the stigma attached to HIV testing was one reason for preferring to order HIVST through the online platform at home.~~

~~*Here, it's like you feel hesitant… But if it's positive, how will people treat you? Even with a negative result, how will people respond? On the other hand, if you do it on your own, in a personal space, you don't feel like you're sitting alone; there's just no one there to judge you. Also, if someone doesn't feel comfortable coming here, it's better for them to take the test on their own and see the results privately. Then they can decide how to proceed from there. MSM7, 23 years old, part time employee, 14 years of education*~~

~~**Subtheme 1.2. Online HIVST ordering allows maintaining confidentiality of HIV status and sexuality**~~

Related the subtheme 1.1, the community stated a benefit of placing the order of HIVST online as it offers more confidentiality both in terms of sexuality and in terms of one's HIV status. The participants stated that in the DIC, others can judge the results of their HIV test through facial expressions, and thus, may lead to a lack of confidentiality, and if the result is positive, it leads to stigma. One participant stated the following:

Because at home, no one else is around in that moment. But if you're doingtaking the test here at the center, your facial expressions and the wayhow you react can give something away. If you get tense or scared, people around you who are watching might notice. They'll realize you're doing an HIV self-test and might assume the result is bad. MSM7, 23 years old, part--time employee, 14 years of education

In comparison with a rapid diagnostic test conducted by a healthcare provider, the participants stated that they preferred placing an online order because they wanted to keep their details confidential from the providers (in comparison to the participants above, who wanted to keep their HIV status and sexuality confidential from community-visiting CBOs.

Though CBOs are a place for the MSM community, there are varying positions, such as paramedics or doctors who are not community members. Participant reported that through digital HIVST dispensation, they have more autonomy in terms of confidentiality.

When we come here, we have to first go to the office and getgive our ID. Then we visit the doctor,; he's a paramedic, and he conducts the test. But at home, we do it ourselves. We already know how. In Punjabi, we say "don't open your mouth… meaning, just do it quietly and easily."FGD1

Another participant stated that those who are cautious about revealing their sexuality or who have social anxiety may prefer placing an online order.

Community members have varying levels of comfort in disclosing their sexuality and varying levels of self-acceptance or self-identity within the MSM spectrum. Coming to CBO may have meaning that they have to identify with a particular identity for which they are not ready. The digital intervention also empowers participants to seek HIV testing services while still processing their identities.

Some people hesitate a lot while coming to the ~~NGO~~CBOs. They don't want to accept (their sexuality) for whatever reason they have. So, self-testing is very easy for them. They can test themselves at their place or anywhere. Some people have social anxiety; they don't want to face it. They don't know what will happen if they come here. ~~So, I think it is very good for them and for me too. Because my house is quite far. It's not easy for me to visit the NGO. So, I also do self-testing.~~ MSM4, 21 years old, graduate, unemployed

~~**Subtheme 1.3. Online ordering of HIVST is more convenient**~~

~~The participants also highlighted that ordering HIVST online is convenient. This is because they do not have to take time out of their routine and place order. Thus, the online order saves time. The other reason for ordering HIVST online was the travel expenses they had to bear while visiting the CBO, which were saved as the CBO delivered HIVST free of cost.~~These findings suggest that digital HIVST dispensation empowers the participants in terms of privacy and confidentiality, thus making it more acceptable.

Subtheme 1.2. HIVST reduces cost and time barriers to HIV testing

Even though HIV testing is free at the CBO, there are time and travel-related costs for availing of on-site HIV testing. Digital HIVST dispensation reduces these barriers, thus participants favor it compared to CBO-based HIV testing. One participant during the FGD stated that

I think it has a lot of benefits, because sometimes you don't have money to go to the ~~NGO~~CBO. They provide it free of cost, including delivery, which is very convenient. You get it for free. Many friends are busy during the day and don't have time. FGD 1

Another participant from the second FGD stated

Yes, I had done the test before. But because of my busy schedule with university and classes, it was a bit difficult to come here. So doing it at home was much easier for me. I ordered it through a link from ~~an online~~a digital platform. .... I received the kit, and I did the test myself. I rubbed the swab on my gums and then dipped it, just like the instructions said. FGD2

~~**Theme 2. Acceptability of the online intervention**~~

These accounts emphasize how digital HIVST dispensation can be taken up by reducing financial and time-related barriers, without interfering with daily routine tasks.

Subtheme ~~2.1. WhatsApp messaging, complemented by~~ 1.3. Peer-driven diffusion of HIV testing to MSM not accessible by the CBOs

Having a favorable experience with the digital HIVST dispensation, there is peer-driven diffusion of HIV services to other MSM. This creates a mechanism of secondary dispensation, allowing an increase in testing coverage and network expansion. This can have a communal benefit. Participants who were not healthcare staff of the CBO stated that they can now provide a link to services to other MSM who have not yet been reached by the CBOs, and would refrain from getting in touch with the CBO staff because of stigma, and maintain privacy. The potential of this digital intervention is recognized by one MSM, who stated that:

If we limit it to CBOs, then it will not reach everyone. MSM are those who don't believe in themselves. It is a good thing to reach them. MSM4, 21 years old, graduate, unemployed

Another participant recalled his experience of getting his friends tested through this service

I feel that the test done here is different from the one done at home.... When two of my friends visited me, I got their tests done at home. MSM5, 22 years old, fashion designer by profession, with 12 years of education

Another participant recalled a similar experience

I found out through an XX worker (at the CBO) that you can even get it (HIVST) delivered to your home. Then I passed that on to my friends I guided them too. I explained how to use the kit and how the process works. I showed them everything step by step. MSM6, 26 years old, paramedic by profession, 14 years of education

~~easy user~~The accounts validate the diffusion of intervention, ensuring network expansion with users playing the role of network expanders.

Subtheme 1.4. Digital guidance and a simple interface~~, makes the online~~ facilitate platform ~~easy to use~~usability

As mentioned above, the current structure of the intervention is the demand generation of HIVST by sharing instructions in the form of videos on how to operate and place an order on the ~~online~~digital platform, and a video on how to perform HIVST. These instructions, as well as the simple interface of the digital platform, are positively appraised by the community, enhancing confidence in the digital platform regarding its usability. During the FGD, the participant stated:

Formatted: Font: Not Bold, Font color: Accent 1

Formatted: Font: Not Bold

Formatted: Font: Times New Roman, 12 pt, Not Bold

Formatted: Font: Times New Roman, 12 pt, Not Bold

Formatted: Heading 2, Left, Line spacing: single, Tab stops: Not at 1.24"

Formatted: Font: Not Bold

It is very good. I had no idea about the website. But the video has made it easy for us. We have sent it to many friends. It is very good. It has a lot of clarity about how to use the website in Urdu, what questions are there, and how to do it. It is very easy. It has all the to-the-point information. FGD1

Another participant highlighted that sending videos on WhatsApp made it easy to use the ~~online~~digital platform.

If you want to book a kit ~~online~~digital, then you have to go to QuickRes, right? Now, they also send videos. Like, XX (CBO worker~~)~~), who sent me the video, he told me that if you want to tell someone further, then first send him a video so that he understands what it is, how to use it. So, sending a video is very beneficial. I also sent a link to him. There are some Facebook friends with whom I have never met, but there is a lot of talk on Facebook. MSM6, 26 years old, paramedic by profession, 14 years of education

The participants acknowledged that the use of the Urdu language made navigation easier on ~~an online~~a digital platform.

No, it was very smooth. The website had a full Urdu language option. I didn't even know it could be done ~~online~~digitally until a friend told me. FGD2

However, these findings were not universal, and some gaps were highlighted by the participants, as given in Theme ~~5~~3.

Theme ~~**3. Potential benefit of digital intervention on community health as a whole**~~

~~**Subtheme 3.1 Online ordering of HIVST can increase HIV testing coverage**~~

~~Beyond personal benefits, digital intervention has a broader impact on community health. Participants who were not healthcare staff of the CBO stated that they can now provide a link to services to other MSM, who have not yet been reached by the CBOs, and would refrain from getting in touch with the CBO staff because of stigma and maintain privacy. The potential of this digital intervention is recognized by one MSM, who stated that:~~

~~*If we limit it to CBOs, then it will not reach everyone. MSM are those who don't believe in themselves. It is a good thing to reach them. MSM4, 21 years old, graduate, unemployed*~~

Formatted: Font: Not Bold

Another participant recalled his experience of getting his friends tested through this service

*I feel that the test done here is different from the one done at home. The test we do*2. Digital platform users trade off between stigma at CBOs vs at home *is private. If we don't want to share our result, we don't have to tell anyone. And we still get to know what our result is. When two of my friends visited me, I got their tests done at home. MSM5, 22 years old, fashion designer by profession with 12 years of education*for HIV testing

Another participant recalled a similar experience

I found out through an XX worker (at the CBO) that you can even get it (HIVST) delivered to your home. Then I passed that on to my friends I guided them too. I explained how to use the kit, how the process works. I showed them everything step by step. MSM6, 26 years old, paramedic by profession, 14 years of education

Theme 4. Perceived stigma and discrimination while placing the online HIVST order

Digital order for HIVST bypasses the stigma faced by individuals in CBO settings. The MSM community faces a similar stigma while ordering HIVST at home and has similar confidentiality issues.

As mentioned above, the digital dispensation of HIVST offers an acceptable private and confidential testing service; it does not eliminate stigma. Stigma from testing in CBOs is replaced by stigma in testing at home. This is compounded by increased scrutiny and a lack of privacy at home. The latter risks enacted stigma, because HIV is linked to sexual transmission and homosexuals, by default. To overcome these barriers, MSM, instead of getting HIV dispensed at home, may choose dispensation in the vicinity to avoid any risk of disclosure. Thus, MSMs weigh the benefits of testing at home as mentioned in the theme above, with potential risks highlighted in this section, choosing a personally suited strategy. However, digital HIVST dispensation provides them with an additional testing option to choose from in HIV services.

Subtheme 42.1. PerceivedFear of enacted stigma while orderingdispensing HIVST at home

The participants reported not being out to family members as MSM, and risk exclusion from the family due to discrimination. One participant reported.

Formatted: Heading 1, Left, Line spacing: single, Tab stops: Not at 1.24"

Formatted: Font: Times New Roman, 12 pt, Not Italic

Formatted: Font: Not Italic

Formatted: Font: Times New Roman, 12 pt, Not Bold

Formatted: Font: Times New Roman, 12 pt, Not Bold

Formatted: Font: Not Bold

Formatted: Heading 2, Left, Line spacing: single, Tab stops: Not at 1.24"

Formatted: Font: Times New Roman, 12 pt, Not Bold

Formatted: Font: Times New Roman, 12 pt, Not Bold

That fear is always there… if your MSM status is disclosed, your reputation (as a straight man) could be tarnished. You have your whole family whose prestige is at stake; it's not just about you. MSM8, 47 years old, artist by profession, 16 years of education

~~stated that they ordered HIVST not at home~~To navigate in this fearful environment, they choose to get HIVST dispensed other than in their homes, but in the vicinity~~, fearing questioning from family members.~~

Many ~~clients~~people do not give the address of their house, but give the ~~pin~~nearby location of their house. For example, if there is a shop or a famous thing, if there is a shop a little ahead of their house, the rider goes there and calls them. FGD 1~~We call the client before sending the kit to confirm the timing and the schedule. FGD 1~~

~~Speaking about stigma, and lack of privacy at home, one participant stated that~~

~~That fear is always there that your privacy might be exposed. And if it does, your reputation (as a straight man) could be tarnished. You have your whole family whose prestige is at stake it's not just about you. MSM8, 47 years old, artist by profession, 16 years of education~~

Another participant in the FGD said that

~~Yes, absolutely the~~The first thing that comes to mind is, where should I have it delivered? Because I can't have it sent to my home. So I try to make sure it's delivered somewhere far from home. Apart from that, I worry about how it will be. It's difficult for a newbie, but for a guide, it's not an issue. FGD2

~~Compared with HIVST at home and in DIC, one MSM stated that~~

For this reason, some MSMs prefer CBO testing, preferring a supportive environment over inadvertent disclosure of sexuality at home. One participant stated that

Here, the environment is friendly. It feels easier and more comfortable. At home, the family doesn't know about this. You have to keep the testing private from them. Naturally, we can't tell them. Some families are friendly, but in some households, you just can't share things like this. I don't think anyone would openly share something like this with their family. The good thing here is that there are friends around at the center, and the environment is friendly like home, but better. Still,

Formatted: Font color: Text 1

Formatted: Font color: Black

if you think about it, going to and from the center takes time. At home, you can just take the test. MSM2, 23 years old, Veterinary doctor by profession

These fears may deter participants from getting HIVST dispensed at home, and instead, they choose a nearby place or prefer HIV testing in the CBO.

Subtheme 4.2. Confidentiality issues2. Lack of privacy at home

InterlinkedLinked closely with the issuefear of perceivedenacted stigma areis the intrusiveness of family members and a lack of confidentiality. MSM stated that they feared that their family might open the HIVST parcel deliveredlack of privacy at home. Family members may later question why they use HIVST.This lack of privacy serves as the mechanism for the fear. The participants reported that hiding HIV testing kits at home is not possible, due to the lack of privacy and questioning in family settings. A participant saidstated that

I don't think anyone can hide it and keep it at home. If he uses a kit, they (family) will check it. He should not keep it in front of his family and face their questions. MSM2, 23 years old, Veterinary doctor by profession

Another MSM stated that

When probed if a change of packaging without mention of HIV testing kits would solve this lack of privacy, a participant responded;

No matter what kind of packaging you use, if someone in the family even has the slightest doubt, they'll open it. Either that, or your reputation at home needs to be so solid that your privacy is respected. MSM8, 47 years old, artist by profession, 16 years of education

However, this lack of privacy and perceived stigma are not universal. One participant highlighted while talking about HIV and self-testing.

The lack of privacy is a concern because of the association with HIV-stigmatized communities. A participant reported that he could justify HIV kits at home because everyone is at risk, and it is not only contracted through sexual means; however, this participant did not challenge the lack of privacy at home either.

Even if you get it delivered to your home, there's no issue because this is common. It's not a gay disease or anything. It can happen randomly, even if you just go to a barber,; their instruments

Formatted: Font: Times New Roman, 12 pt, Not Bold

Formatted: Font: Times New Roman, 12 pt, Not Bold

Formatted: Heading 2, Left, Line spacing: single, Tab stops: Not at 1.24"

Formatted: Font: Times New Roman, 12 pt, Not Bold

Formatted: Font: Not Bold

Formatted: Font color: Text 1

can also be a source. This is a disease that can be contracted in many ways. ~~So it's not necessary that~~So family members don't need to find out about it just because it's ordered at home. MSM9, 24 years old, working in an advertising agency, 14 years of education

The above excerpts from the interview focus on the prevalent generalized stigma against the HIV and MSM community in the households, triggering fear and posing barriers to HIV testing.

Theme ~~5. Gaps and recommendations for strengthening~~3. HIVST digital platform needs system maturation, demand generation, and integration in broader HIV services

Although the intervention was valued by the participants, several technical limitations were also highlighted, which may impact sustainability. In addition, the participants called for system integration with comprehensive HIV services. The finding reflects community readiness and the realization of a need for associated services. Participants also highlighted that for maximum impact, diverse digital demand generation is needed, leveraging social media and dating applications. However, digital interventions should not replace human interaction, both in demand generation at the community level but also individual counselling for service users.

~~Some participants stated that the use of video messaging made online platform navigation easy. Some participants highlighted these gaps. These were sorted into two sub-themes. Subtheme 5.1 pertains to the design and structure of the online platform, and Subtheme 5.2 pertains to the content of the platform.~~

~~Subtheme 5.1. Problems~~Subtheme 3.1. Users face problems related to the design and structure of the ~~online~~digital platform

~~Participants identified several issues related to the design and structure of online platforms. The most important of these platforms is their presence. One for information on HIV, including the use of condoms, lubricants, etc., and one for HIV. The online platform is described by some participants as difficult to navigate and confusing. One participant stated the following:~~

~~*Basically, its link was very difficult. I want that if a person who does not know how to write or read goes to their link, and have an option to call. Call and get help. Not everyone knows how to write. People are uneducated. You already know how low the literacy rate is. For someone who can't read or write properly, ordering a kit online can be really difficult.*~~ The current structure of having two platforms, one for HIV services and one for HIVST dispensation, was seen as complex by participants. The participants reported inconsistent linkages between the two, which make

Formatted: Font: Times New Roman, 12 pt, Not Bold

Formatted: Font: Times New Roman, 12 pt, Not Bold

Formatted: Font: Not Bold

Formatted: Font: Not Bold

Formatted: Heading 2, Left, Line spacing: single, Tab stops: Not at 1.24"

Formatted: Font: Times New Roman, 12 pt, Not Bold

Formatted: Font: Times New Roman, 12 pt, Not Bold

Formatted: Font: Not Bold

navigation difficult. Thus, optimal operation of the platforms requires a good level of digital literacy and comfort. However, given the literacy levels in MSM, who are marginalized, this is not optimal. These may limit the use of the platform to certain MSM rather than the MSM community as a whole. One participant stated that the platform is not designed for average MSM.

This platform isn't really for the average person; only someone who's a graduate or at least well-educated can manage to place an order. An uneducated or less literate person wouldn't be able to do it easily. Only graduates can do this. But there should also be an option to call. You should call and receive a call from the representative. You should tell him all the details, your address, and it should reach you. FGD2

Since HIVST can be ordered from Quickres, but HIV prevention information is on the Sehat Dost platform, it may be cumbersome for some participants, as one participant stated:

As far as I know, both things should be on the same platform....So, I will not need to visit other platforms.... MSM6, 26 years old, paramedic by profession, 14 years of education

Another participant highlighted that even among the two platforms, the linkage is inadequate

No, there's no option for Sehat Dost in QuickRes. The QuickRes option is available on Sehat Dost, but not vice versa. FGD2

This lack of linkage between platforms causes people to miss content on the complementary platform.

Some people know about it, and some don't. But it depends on the chain. As I know, if someone consults me, I will guide them properly. MSM6, 26 years old, paramedic by profession, 14 years of education

Apart from having two platforms as part of the digital intervention, participants also highlightedreported some technical problems related to bookings. One participant stated the following:

There are time slots shown inat the beginning; you're asked to select one for delivery. Different time slots appear, and you're supposed to choose one. But at first, they don't get selected. Then, when you move to the next page, it shows that the slots are available, but on the next screen, they

aren't. The system just assigns a random time on its own sometimes at a time when you're not available or there is any problem, then… MSM1, 25 years old, lawyer by profession

These reflections emphasize the need for system maturation, comprehensive single platforms designed for those with low digital literacy for maximum utilization.

Subtheme ~~5~~3.2. ~~Problems~~Users face problems related to the content of the platform

The current language and data collection at the digital platform reflect use of technical jargon related to sexual identities. This creates confusion and mistrust among users. In addition, long data collection creates fear of data security and privacy. The participants' emphasis on these points reflects that the current content is not user-centered. ~~Some participants also criticized the platform for asking for too much information before booking.~~

~~*I saw their questions; there were quite a lot, maybe 2–3 pages worth. They were very detailed. So either the questions should be shortened a bit, or made more efficient, so that it saves people time. MSM5, 22 years old, fashion designer by profession, with 12 years of education*~~

Speaking about the technical language used on the platform, one participant stated that

There are a few questions that confuse people, like MSW, MSM, or bisexual. When we explain that these terms are for the community and that there's nothing to be afraid of and that their data is safe, then they're more comfortable. Some people did ask me what MSW, MSM, or bisexual means. These are just written differently, and people often get confused. But it's not a problem once it's explained~~,~~; they're fine. Yes, people are a little afraid at first. FGD2

Some participants also criticized the platform for asking for too much information before booking. The participants are concerned about digital privacy, as giving personal information on a digital platform has risks and triggers anxiety.

I saw their questions; there were quite a lot, maybe 2–3 pages worth. They were very detailed. MSM5, 22 years old, fashion designer by profession, with 12 years of education

Thus, the content needs to be centered around user-friendly language, which shall improve trust. In addition, the collection of personal information should be minimized to encourage service use.

Formatted: Font: Times New Roman, 12 pt, Not Bold

Formatted: Font: Times New Roman, 12 pt, Not Bold

Formatted: Heading 2, Left, Line spacing: single, Tab stops: Not at 1.24"

Formatted: Font: Times New Roman, 12 pt, Not Bold

Formatted: Font: Not Bold

Subtheme ~~5.3. Community recommends~~3.3. Users request the provision of comprehensive services through ~~online~~digital order

~~We also asked participants about any~~Participants have recommended the provision of comprehensive HIV services ~~they thought should be included in the current package of~~ digitally, reflecting the community's readiness for uptake of digital services ~~through digital intervention. The participants stated that comprehensive~~ . These services should ~~be provided. This includes the provision of~~ not be limited to HIVST but include condoms and lubricants as well as digital counselling ~~services.~~for HIV test-takers.

One participant stated that

~~At least when it comes to medication, you could say that it can't be provided without a prescription, so you have to go through that process. But when it comes to condoms~~Condoms or lubricants, I believe ~~those~~, should be made available. ~~Otherwise, if you look at how it's done in other countries, you either upload a prescription or hand it over directly. They usually have a system; for example, if someone provides a phone number, it gets linked to their data, and everything is saved. So based on that data, your medication is sent to you directly.~~. MSM 2

Similarly, another participant ~~stated~~emphasized the same idea that

And apart from that, there should be an option for condoms as well. For example, if someone wants to use condoms but doesn't feel comfortable asking for them openly, then they should be able to get them delivered discreetly to their home, too. That would also be very beneficial in terms of protection. MSM6, 26 years old, paramedic by profession, 14 years of education

Other participants recommended ~~online~~HIV digital counselling. One participant ~~suggested counselling online with a counsellor~~highlighted the need for the same as

It is ~~an online~~a digital service, and they are providing a kit, so counselling should also be done as the kits are provided. There can be ~~online~~digital counselling, and it can also be done via video call. Now, obviously, it is the age of digital. If there can be ~~online~~digital classes~~, online~~ and digital meetings. So why can't counselling be done ~~online~~digitally? MSM 2

These excerpts highlight that the digital intervention can have a scope of expansion to comprehensive services, given the community demand.

Formatted: Font: Times New Roman, 12 pt, Not Bold

Formatted: Heading 2, Left, Line spacing: single, Tab stops: Not at 1.24"

Formatted: Font: Times New Roman, 12 pt, Not Bold

Formatted: Font: Times New Roman, 12 pt, Not Bold

Formatted: Font: Not Bold

Formatted: Tab stops: 1.24", Left

Subtheme 53.4. DemandUsing diverse demand generation through personal and community in-person interactions, as well as online and digital means such as

The participants recommended the need for a multipronged digital demand generation. These include outreach at dating applications and, as well as social media

The interviewed community members provided recommendations on demand generation for digital services. Few have recommended sharing links to digital platforms after personal communication, whereas others have recommended the use of social media and dating applications for this purpose.

. However, digital demand generation should not replace human interaction. In fact, interpersonal interaction can complement digital demand generation, ensuring uptake of services. One participant stated that

We can't send it through just by sharing the link, as it is sensitive. You can only share it after a deep conversation. Only then will the next person understand it and be able to use it positively. You can also give advertisements on Grindr and other dating applications. FGD1

While some people have stressed the use of dating applications for advertising, others have stated the use of social media applications for the demand generation of HIV services and self-testing.

Not everyone uses Grindr. For example, I don't use Grindr. Most people use Instagram and Facebook (for hookups and dates). Even within the gay community, not everyone is on Grindr. MSM7, 23 years old, part-time employee, 14 years of education

One participant stated that there should be group sessions to complement digital messaging, and stated that people may take digital messaging for granted.

There should be more awareness sessions in the CBOs, too. They should hold regular sessions so people can learn. Because most people don't read everything on the app. They either skip through or just don't bother going into full detail. FGD2

These reflections from the participants highlight the need for multipronged and well-connected digital outreach and demand generation, leveraging social media and dating applications, with interpersonal interactions, individually or as a group, rather than just sharing a platform link.

Discussion

Formatted: Font: Times New Roman, 12 pt, Not Bold

Formatted: Font: Times New Roman, 12 pt, Not Bold

Formatted: Font: Times New Roman, 12 pt, Not Bold

Formatted: Font: Times New Roman, 12 pt, Not Bold

Formatted: Font: Times New Roman, 12 pt, Not Bold

Formatted: Font: Not Bold, Font color: Black

Formatted: Font: Not Bold, Font color: Black

~~To the best of our knowledge, the current study is the second study that evaluated~~***Framework for evaluating* digital HIVST** ~~in a Muslim-majority country. A previous study conducted in Malaysia evaluated a digital application that provided a comprehensive HIV prevention package~~***dispensation***

Formatted: Font: Bold, Italic

The real-world effectiveness of interventions depends on several factors, including factors other than the characteristics of the intervention itself. [14,15]Various theories, models, and frameworks have been proposed to ascertain the effectiveness of interventions.[14,15] One of the most influential frameworks is CFIR, proposed by Damschroder~~HIVST and pre-exposure prophylaxis (PrEP).(Shrestha~~ et al~~., 2023) This study employed a pre-post design. Among 50 MSM using the application, 84% used HIVST, with 42% ordering more than once.(Shrestha et al., 2023) Globally, HIVST delivery through digital platforms is acceptable~~. The framework includes five major domains for evaluating complex interventions: innovation, process, inner setting, outer setting, and ~~accurate.(Beecroft et al., 2025)~~ individual characteristics of users, as well as facilitators and implementers.[13] Our evaluation highlights that each of these domains influences user experiences and contributes to intervention uptake.

Formatted: Font color: Text 1

Formatted: Font color: Text 1

Acceptability of HIVST intervention

Zhu et al. conducted a randomized controlled trial comparing HIVST delivery through digital ~~ordering~~dispensation and in-person dispensation. The study showed that the option of digital ~~ordering~~dispensation increased HIV testing two-fold.~~(Zhu et al., 2019)~~ [18] In two separate RCTs that used Internet-based HIVST dispensation, it was noted that testing rates increased 1.47 to 1.77-fold. ~~(Young et al., 2022)~~[19]~~(Wang et al., 2018)However, intervention design in Pakistan has major limitations. One of these is the lack of comprehensive HIV prevention services.~~ However, there is significant heterogeneity observed in acceptance and preference for digital HIVST interventions. In a recent review, the acceptance of digital HIVST ranged from 64.55% to 99.0%. The preference for digital HIVST interventions ranged as low as 4.6% to 99.6%.[20] In a meta-analysis pooling results of digital HIVST interventions from low- and middle-income countries, HIV testing uptake was similar between digital and non-digital interventions.[21]

The participants in our sample emphasize the convenience and resource efficiency of digital dispensation of HIVST, stating relative advantage over facility-based HIV testing. In a previous pilot intervention of HIVST dispensation through CBO workers, 96.6% of MSM reported HIVST

convenient to use. In the study, 55.3% of MSM preferred outreach through social media.[22] The current evaluation highlights that HIVST itself and its digital dispensation are acceptable to the MSM community. However, there are several caveats to this acceptability and preference which are covered in sections below.

Digital HIVST dispensation, stigma and mental health support

MSMs are marginalized in many muslim-majority countries, with Pakistan being one of them.[3] This poses unique challenges in reaching out to the community with health and HIV interventions. Stigma is faced in family settings, legal frameworks, as well as internalized or perceived stigma within community settings[23]. In the current study, participants have described how stigma, even within community settings such as CBOs, serves as a barrier to HIV testing uptake. HIVST through digital means provides an additional testing option for the MSM community in that context. However, the stigma in CBO testing is replaced by fear of stigma in home settings, an interaction of inner and outer settings. Thus, digital dispensation of HIVST is influenced by stigma but in different contexts. The fear of enacted stigma is not unfounded, as MSM and people living with HIV (PLHIV) are highly stigmatized in the context of Pakistan. In the National HIV Stigma Index 2.0, 10% of MSM living with HIV reported that they were excluded from family settings in the last twelve months because of their identity as MSM.[5] Around 21.53% of MSM PLHIV also reported that they felt they were gossiped about and discriminated against by family in the last year because they are MSM. The stigma is not limited to same sex prefrences or behaviors. PLHIV are also excluded and discriminated against in family settings.[5] Around 5% of PLHIV report that they are excluded from family events in the last twelve months. Due to this stigma, people refrain from sharing their HIV status with their family.[5] In the National Stigma Index 2.0, 26.6% of MSM PLHIV reported they feel uncomfortable sharing their HIV status with those close to them due to stigma. Testing in such a stigmatizing environment thus brings anxiety, as reported by the participants[5]. In a meta-synthesis of HIVST interventions among MSM in Asia, it was found that mental distress is in fact a barrier for HIVST uptake.[10] The participants from our study have, in fact, stressed the need for counselling services, though provided digitally for users of digital dispensation.

Comprehensive service provision

According to the World Health Organization, comprehensive HIV prevention services include HIV testing services, condoms, PrEP, and the management of sexually transmitted infections.(Consolidated Guidelines on HIV, Viral Hepatitis and STI Prevention, Diagnosis, Treatment and Care for Key Populations, 2022) [24] The digital platform in our intervention provided HIV informational care and HIVST kits. The participants, however, recommended comprehensive services that they can access at CBOs, such as condoms and lubricants. This reflects the participants' needs and readiness for uptake of services. Provision of these services shall also require 'relational connections' with law enforcement agencies. Condoms are not positively seen in society, and dispensation using riders may precipitate harassment from police.[25] Online prescription and dispensation of PrEP can also be explored. However, medical regulators' mechanisms for online assessment and prescription shall need to be assessed for implementation. In Malaysia, another muslim-majority context, researchers have evaluated a digital application that provided a comprehensive HIV prevention package, including HIVST and PrEP. [26] This study employed a pre-post design. Among 50 MSM using the application, 84% used HIVST, with 42% receiving dispensation more than once.[26]

Digital platform integration

While information on these is available on the Sehat Dost platform, participants highlighted that the presence of a separate platform for HIVST orderingdispensation (Quickres) may be cumbersome to use.

Previously, two community pilot projects were evaluated for HIVST using OraQuick, one each for MSM and transgender individuals.(Altaf et al., 2022, 2023) Both pilot projects involved the dispensation of HIVST through outreach workers based on CBOs. Among transgender individuals, 90.6% reported that HIVST was easy to perform and 95% stated that the instructions for HIVST were easy. Approximately 95% of transgender individuals in the study stated that they would recommend HIVST to community friends.(Altaf et al., 2022) Although the current model of intervention relies on digital orderingdispensation and mailing of HIVST, participants stated that they shared the link ofto the online orderingdigital dispensation platform with other community members. It should be noted that this method may be used to engage MSM who cannot be reached by conventional outreach by CBOs. These themes illustrate the need for innovation and adaptability. In addition, the users also highlighted provider-centered language and data capture

Formatted: Font color: Black

that need to be addressed. These recommendations are important, given that innovation is inherently diffusing to late adopters, who may be less motivated to use the service.[27] Providing client-centered and easy usability of the platform will improve user satisfaction and increase use of the platform.

Demand generation

In terms of demand generation, the participants in the current study emphasized digital means. This includes the use of social media applications, which may be used for various purposes but may include cruising clients or dates. These platforms include Facebook and Instagram. However, the participants also highlighted the use of digital dating applications exclusively by MSM, such as Grindr or Blued. In the previous pilot of HIVST, which relied on in-person HIVST dispensation, 55.3% of MSM preferred social media, while 45.3% preferred peer-to-peer methods for demand generation.(Altaf et al., 2023) While the profile and risk behaviors of MSM who use dating applications have not been studied in Pakistan, it is not known if their risk behaviors are higher than those of MSM who do not use dating applications. Researchers from the United States have shown that MSM using Grindr had higher rates of chlamydia or gonorrhea infection than those who did not use Grindr, higher rates of HIV infection, condomless anal intercourse, and a higher number of male sexual partners. However, the cohort was more likely to initiate HIV PrEP than non-users.(Hoenigl et al., 2020)[28] This highlights the systematic differences among MSM who use dating applications. Thus, the digital dispensation of HIVST can be an effective method for engaging this community by linking Quickres with dating applications for demand generation. However, it should also be noted that dating applications are banned in Pakistan by the government; thus, advertising on these applications may require unbanning of the applications. Despite being banned by the government, proxy web administrators use these applications.(Gibran Naiyyar Peshimam, 2020)[29]

Peer-driven intervention diffusion

Most of the study participants were young, educated, and employed. Previous research has shown that young and socioeconomically advantaged individuals are adopters of health technology. In addition, all participants in IDIs had index HIVST in DIC, followed by ordering HIVST on a digital platform. This highlights the increasing confidence of the MSM community in the HIVST intervention. According to the social network model of diffusion of innovation, opinion leaders

help uptake of innovation or intervention. Our study highlights that the MSM were first promoted for use of HIVST in the DIC, followed by ordering through the digital platform. The study participants also highlighted that they recommended digital HIVST intervention to their peers, who were previously out of network reach by the CBOs. Thus, the intervention was able to reach them through social networks. This can increase the testing coverage of HIV among MSM in Pakistan. The global targets for HIV by 2025 were that 95% of people living with HIV know their status.[2] In the last national HIV strategy 2021-2025, it was revealed that only 2% of MSM had been tested for HIV in the last 12 months.[6] According to the National AIDS Control Program (www.cmu.gov.pk), 84,421 PLHIV know their HIV status out of 330,000 infected. This reflects that only 25% of PLHIV in the country are aware of their HIV status. The use of digital HIVST intervention can thus offer support in increasing testing coverage for the MSM community in the country.

Limitation of the study

The current study, however, has several limitations. First, it explored the qualitative experiences of the participants. In addition,The participants were recruited through non-probability sampling usinginterviewed by a researcher who was a former manager at the CBO staff, thus introducing selection; this may induce reporting and desirability bias., where participants only reported favorable themes. The participants were from two cities in Pakistan; thus, their experience may not be generalizable to all MSM residing in the country. Furthermore, we only used CFIR for post-hoc inductive analysis. CFIR is a comprehensive tool for appraisal and planning of intervention. Many of the CFIR domains are related to implementer, organizational, and donor perspectives. Our study, thus, uses CFIR as a lens for capturing user perspectives while not evaluating the intervention from the viewpoint of other stakeholders. Nonetheless, the study has several strengths allowing for greater understanding of how outer settings influence HIVST digital dispensation and how the innovation can be adapted for improved efficiency.

Conclusion

OnlineDigital HIVST orderingdispensation is acceptable given regular client support by a community-based organization (CBOs) with the potential to increase HIV testing coverage through peer-to-peer sharing of an online ordering platform.. However, participants noted difficulty in platform navigation, confidentiality,. While digital dispensation empowers people to

have greater control over HIV and sexuality disclosure, it also introduces vulnerability of disclosure and stigma ~~issues by ordering HIVST at~~ in home~~, and a lack of~~ settings. There is a community readiness to uptake a comprehensive ~~condoms, lubricants, and online counselling~~package of HIV digitally, in addition to HIVST. The intervention also holds promise of network expansion of services~~.~~ driven by peers to MSM not reached by the CBOs. The participants ~~also highlighted the need for~~recommend digital demand generation ~~through social media and dating applications. Provision of a comprehensive service delivery package, digital~~ and interpersonal interaction at demand generation ~~through social media, and dating applications is recommended for strengthening online HIVST services.~~ as well as the testing stage in the form of video counselling. The current banning of MSM dating sites poses a significant barrier to demand generation.

Authors contribution

References

[1] Integrated Biological and Behavioral Surveillance of HIV in Pakistan: Round 5 - 2016-17. 2017.

~~Adu, & Philip. (2019). *A Step-by-Step Guide to Qualitative Data Coding*.~~

[2] AIDS, crisis and the power to transform: UNAIDS Global AIDS Update. ~~(~~2025~~).~~ ~~http://www.wipo.int/amc/en/mediation/rules.~~

[3] Human Dignity Trust. Map of Jurisdictions that Criminalise LGBT People. ~~Ali, U., & Ali, S. A. (2023). Prevalence of Depression and Subjective Job Stress Among Men who have Sex with Men and Transgender~~Https://WwwHumandignitytrustOrg/ 2024.

[4] Hear us out: Community ~~Health~~HIV measuring HIV-related stigma and discrimination. 2023.

[5] Usman Ali. National HIV Stigma Index. 2024.

[6] Pakistan AIDS Strategy IV 2021-2025. 2020.

[7] End inequalities, end AIDS. Global AIDS Strategy 2021-2026. 2020.

[8] Ali U, Fatima B, Azmat S, Khan MS, Chaudhary R, Mirzazadeh A. The Effect of Emotional Intelligence and Employment on the Psychological Well-Being of HIV Key Population Workers~~ in Pakistan~~. Journal of the College of Physicians and Surgeons Pakistan~~, *33(7)*, 779–783.~~ 2025;2025:1294–300. https://doi.org/10.29271/jcpsp.~~2023.07.779~~2025.10.1294.

Formatted: Font: Not Italic, Font color: Black
Formatted: Font color: Black
Formatted: Font color: Black
Formatted: Indent: Left: 0.44", Hanging: 0.44"
Formatted: Font color: Black
Formatted: Font color: Black
Formatted: Font: Not Italic, Font color: Black
Formatted: Font color: Black
Formatted: Font color: Black
Formatted: Indent: Left: 0.44", Hanging: 0.44"
Formatted: Font color: Black
Formatted: Font color: Black
Formatted: Font: Not Italic, Font color: Black
Formatted: Font color: Black
Formatted: Font color: Black

[9] Ali, U., Pasha, S. K., SK, Patel, R., Schmidt, H. M., & HM, Altaf, A. (2023), Assessment of Acceptability of Pre-exposure Prophylaxis for HIV Prevention Among Men Who Have Sex with Men and Transgender Community in Pakistan. Journal of the College of Physicians and Surgeons Pakistan, 2023;33(9), :983–9899. https://doi.org/10.29271/jcpsp.2023.09.983.

[10] Pasha SK, Ali U. Facilitators, Barriers, and Recommendations for Overcoming Barriers to HIV Self-Testing (HIVST) Uptake Among Men Who Have Sex with Men and Transgender People in Asia: A Meta-Synthesis. J Coll Physicians Surg Pak 2025;35.

[11] EpiC. QuickRes Technical Guide; A Global Online Reservation and Case Management Tool for HIV Programs. 2020.

[12] Dhakal K. NVivo. Journal of the Medical Library AssociationAltaf, A., Pasha, M. S. K., Majeed, A., Ali, W., Alaama, A. S., & Jamil, M. S. (2022). Acceptability and feasibility of HIV self-testing among transgender people in Larkana, Pakistan: Results from a pilot project. *PloS One*, *17*(7), e0270857. https://doi.org/10.1371/journal.pone.0270857

Altaf, A., Pasha, M. S. K., Wali, M. S., & Majeed, A. (2023). 2022;110. https://doi.org/10.5195/jmla.2022.1271.

[13] Damschroder LJ, Reardon CM, Opra Widerquist MA, Lowery J. Conceptualizing outcomes for use with the Consolidated Framework for Implementation Research (CFIR): the CFIR Outcomes Addendum. Implementation Science 2022;17. https://doi.org/10.1186/s13012-021-01181-5.

[14] Longworth GR, Goh K, Agnello DM, Messiha K, Beeckman M, Zapata-Restrepo JR, et al. A review of implementation and evaluation frameworks for public health interventions to inform co-creation: a Health CASCADE study. Health Res Policy Syst 2024;22. https://doi.org/10.1186/s12961-024-01126-6.

[15] Damschroder LJ, Aron DC, Keith RE, Kirsh SR, Alexander JA, Lowery JC. Fostering implementation of health services research findings into practice: A consolidated framework for advancing implementation science. Implementation Science 2009;4. https://doi.org/10.1186/1748-5908-4-50.

[16] Adu, Philip. A Step-by-Step Guide to Qualitative Data Coding. 2019.

[17] Tong A, Sainsbury P, Craig J. Consolidated criteria for reporting qualitative research (COREQ): a 32-item checklist for interviews and focus groups. International Journal for Quality in Health Care 2007;19:349–57. https://doi.org/10.1093/intqhc/mzm042.

[18] Zhu X, Zhang W, Operario D, Zhao Y, Shi A, Zhang Z, et al. Effects of a Mobile Health Intervention to Promote HIV Self-testing with MSM in China: A Randomized Controlled Trial. AIDS Behav 2019;23:3129–39. https://doi.org/10.1007/s10461-019-02452-5.

[19] Young SD, Cumberland WG, Singh P, Coates T. A Peer-Led Online Community to Increase HIV Self-Testing Among African American and Latinx MSM: A Randomized

Formatted: Font color: Black

Formatted: Font color: Black

Formatted: Font color: Black

Formatted: Font color: Black

Formatted: Font color: Black

Formatted: Font color: Black

Formatted: Font color: Black

Formatted: Font color: Black

Formatted: Font color: Black

Formatted: Font: Not Italic, Font color: Black

Formatted: Font: Not Italic, Font color: Black

Formatted: Font color: Black

Formatted: Font color: Black

Formatted: Font color: Black

Formatted: Font color: Black

Formatted: Font: Not Italic, Font color: Black

Formatted: Font color: Black

Formatted: Font color: Black

Formatted: Font: Not Italic, Font color: Black

Formatted: Font color: Black

Formatted: Font: Not Italic, Font color: Black

Formatted: Font color: Black

Formatted: Font color: Black

Controlled Trial. J Acquir Immune Defic Syndr (1988) 2022;90:20–6. https://doi.org/10.1097/QAI.0000000000002919.

[20] Beecroft A, Vaikla O, Engel N, Duchaine T, Liang C, Pant Pai N. Evidence on Digital HIV Self-Testing From Accuracy to Impact: Updated Systematic Review. J Med Internet Res 2025;27:e63110. https://doi.org/10.2196/63110.

[21] Nicely P, Xiang L, Smith-Sreen J, Garbern SC, Aluisio AR. Approaches and impacts of digital health in HIV self-testing uptake & use among populations from low- and middle-income countries: a descriptive systematic review and meta-analysis. J Public Health Emerg 2025;9:11–11. https://doi.org/10.21037/jphe-24-56.

[22] Altaf A, Pasha MSK, Wali MS, Majeed A. Acceptability and feasibility of intra-peer and social media approach for HIV self-testing among men who have sex with men in Karachi: lessons from a pilot study. *Journal of the Pakistan Medical Association,* J Pak Med Assoc 2023;73(3). https://doi.org/10.47391/JPMA.6846.

Beecroft, A., Vaikla, O., Engel, N., Duchaine, T., Liang, C., & Pant Pai, N. (2025).[23] Batchelder AW, Foley JD, Kim J, Thiim A, Kelly J, Mayer K, et al. Intersecting internalized stigmas and HIV self-care among men who have sex with men and who use substances. Soc Sci Med 2021;275. https://doi.org/10.1016/j.socscimed.2021.113824.

[24] Evidence on Digital HIV Self-Testing From Accuracy to Impact: Updated Systematic Review. *Journal of Medical Internet Research*, *27*, e63110. https://doi.org/10.2196/63110

Consolidated guidelines on HIV, viral hepatitis and STI prevention, diagnosis, treatment and care for key populations. (2022).

[25] Bano S, Rahat R, Fischer F. Inconsistent condom use for prevention of HIV/STIs among street-based transgender sex workers in Lahore, Pakistan: socio-ecological analysis based on a qualitative study. BMC Public Health 2023;23:635. https://doi.org/10.1186/s12889-023-15550-w.

[26] Shrestha R, Altice FL, Khati A, Azwa I, Gautam K, Gupta S, et al. Clinic-Integrated Smartphone App (JomPrEP) to Improve Uptake of HIV Testing and Pre-exposure Prophylaxis Among Men Who Have Sex With Men in Malaysia: Mixed Methods Evaluation of Usability and Acceptability. Dhakal, K. (2022).JMIR Mhealth Uhealth 2023;11:e44468. NVivo. Journal of the Medical Library Association, *110*(2). https://doi.org/10.5195/jmla.2022.12712196/44468.

End inequalities, end AIDS. Global AIDS Strategy 2021-2026. (2020). https://www.unaids.org/sites/default/files/PCBSS_Global_AIDS_Strategy_2021--2026_EN.pdf

EpiC. (2020).[27] Van Houtven CH, Drake C, Malo TL, Decosimo K, Tucker M, Sullivan C, et al. Ready, set, go! The role of organizational readiness to predict adoption of a family caregiver training program using the Rogers' diffusion of innovation theory. Implement Sci Commun 2023;4:69. https://doi.org/10.1186/s43058-023-00447-x.

Formatted: Font color: Black

Formatted: Font color: Black

Formatted: Indent: Left: 0.44", Hanging: 0.44"

Formatted: Font: Not Italic, Font color: Black

Formatted: Font color: Black

Formatted: Font color: Black

Formatted: Font color: Black

Formatted: Font: Not Italic, Font color: Black

Formatted: Indent: Left: 0.44", Hanging: 0.44"

Formatted: Font color: Black

Formatted: Font color: Black

Formatted: Font color: Black

Formatted: Indent: Left: 0.44", Hanging: 0.44"

Formatted: Font color: Black

Formatted: Font color: Black

Formatted: Font: Not Italic, Font color: Black

Formatted: Font color: Black

Formatted: Font color: Black

[28] QuickRes Technical Guide; A Global Online Reservation and Case Management Tool for HIV Programs. https://quickres.org

Gibran Naiyyar Peshimam. (2020, September 2). Pakistan blocks five dating apps including Tinder and Grindr. Reuters.

Hear us out: Community HIV measuring HIV-related stigma and discrimination. (2023). https://www.stigmaindex.org/wp-content/uploads/2023/11/PLHIV-Stigma-Index-Global-Report-2023-2.pdf

Hoenigl, M., Little, S. J., SJ, Grelotti, D., Skaathun, B., Wagner, G. A., GA, Weibel, N., Stockman, J. K., & Smith, D. M. (2020)., et al. Grindr Users Take More Risks, but Are More Open to Human Immunodeficiency Virus (HIV) Pre-exposure Prophylaxis: Could This Dating App Provide a Platform for HIV Prevention Outreach? Clinical Infectious Diseases, 2020;71(7), ;e135–e14040. https://doi.org/10.1093/cid/ciz1093.

Human Dignity Trust. (2024). Map of Jurisdictions that Criminalise LGBT People. Https://Www.Humandignitytrust.Org/.

Integrated Biological and Behavioral Surveillance of HIV in Pakistan: Round 5 - 2016-17. (2017).

Pakistan AIDS Strategy IV 2021-2025. (2020).

Shrestha, R., Altice, F. L., Khati, A., Azwa, I., Gautam, K., Gupta, S., Sullivan, P. S., Ni, Z., Kamarulzaman, A., Phiphatkunarnon, P., & Wickersham, J. A. (2023). Clinic-Integrated Smartphone App (JomPrEP) to Improve Uptake of HIV Testing and Pre-exposure Prophylaxis Among Men Who Have Sex With Men in Malaysia: Mixed Methods Evaluation of Usability and Acceptability. *JMIR MHealth and UHealth*, *11*, e44468. https://doi.org/10.2196/44468

Tong, A., Sainsbury, P., & Craig, J. (2007). Consolidated criteria for reporting qualitative research (COREQ): a 32-item checklist for interviews and focus groups. International Journal for Quality in Health Care, *19*(6), 349–357. https://doi.org/10.1093/intqhc/mzm042

Usman Ali. (2024). *National HIV Stigma Index*.

Wang, Z., Lau, J. T. F., Ip, M., Ho, S. P. Y., Mo, P. K. H., Latkin, C., Ma, Y. L., & Kim, Y. (2018). A Randomized Controlled Trial Evaluating Efficacy of Promoting a Home-Based HIV Self-Testing with Online Counseling on Increasing HIV Testing Among Men Who Have Sex with Men. *AIDS and Behavior*, *22*(1), 190–201. https://doi.org/10.1007/s10461-017-1887-2

Young, S. D., Cumberland, W. G., Singh, P., & Coates, T. (2022). A Peer-Led Online Community to Increase HIV Self-Testing Among African American and Latinx MSM: A Randomized Controlled Trial. *Journal of Acquired Immune Deficiency Syndromes*, *90*(1), 20–26. https://doi.org/10.1097/QAI.0000000000002919

Formatted: Font color: Black
Formatted: Font: Not Italic, Font color: Black
Formatted: Font color: Black
Formatted: Font color: Black
Formatted: Font: Not Italic, Font color: Black
Formatted: Font color: Black
Formatted: Font color: Black
Formatted: Font color: Black
Formatted: Font color: Black
Formatted: Font color: Black
Formatted: Font color: Black
Formatted: Font color: Black
Formatted: Indent: Left: 0.44", Hanging: 0.44"
Formatted: Font color: Black
Formatted: Font color: Black
Formatted: Font color: Black
Formatted: Font color: Black
Formatted: Font: Not Italic, Font color: Black
Formatted: Font: Not Italic, Font color: Black
Formatted: Font color: Black
Formatted: Font color: Black
Formatted: Font color: Black
Formatted: Font color: Black
Formatted: Font: Not Italic, Font color: Black
Formatted: Font color: Black
Formatted: Font color: Black
Formatted: Font color: Black
Formatted: Font: Not Italic, Font color: Black
Formatted: Font color: Black

Zhu, X., Zhang, W., Operario, D., Zhao, Y., Shi, A., Zhang, Z., Gao, P., Perez, A., Wang, J., Zaller, N., Yang, C., Sun, Y., & Zhang, H. (2019). Effects of a Mobile Health Intervention to Promote HIV Self-testing with MSM in China: A Randomized Controlled Trial. [29] Gibran Naiyyar Peshimam. Pakistan blocks five dating apps including Tinder and Grindr. Reuters*AIDS and Behavior*, *23*(11), 3129–3139. https://doi.org/10.1007/s10461-019-02452-5 2020.

Formatted: Font color: Black

Formatted: Font color: Black

Formatted: Font: Not Italic, Font color: Black

The Editor,

PLOS Global Public Health,

22 June 2026

Dear Editor,

Thank you for sharing the reviewers' comments for the manuscript. I am making pointwise response to the concerns raised below.

Reviewer #2: Summary

This study qualitatively assesses a digital HIV self-testing (HIVST) intervention among men who have sex with men (MSM) in Pakistan, focusing on user experiences with an online platform that allows ordering of OraQuick self-test kits for home delivery. Using 12 in-depth interviews and two focus group discussions, the authors found that digital HIVST is generally acceptable and preferred because it reduces stigma, enhances confidentiality, and offers convenience compared to facility-based testing. Participants also noted the importance of peer sharing and digital platforms in potentially increasing HIV testing uptake within the MSM community. However, they identified key barriers, including concerns about confidentiality at home, ongoing stigma, challenges in navigating the platform, and fragmentation between different digital services. Participants further emphasized the need for a more comprehensive service package, including condoms, lubricants, and online counseling, as well as improved demand generation through social media and dating apps. Overall, the study indicates that while digital HIVST holds great potential in this context, optimizing platform design, service integration, and delivery strategies is necessary for effective scale-up.

Major Comments

1. The study does not clearly demonstrate whether data saturation was achieved, which is essential for qualitative rigor.

Data collection and preliminary analysis were conducted concurrently. Saturation was considered achieved when no new codes or concepts emerged from successive interviews. Saturation was reached after ten IDIs, following which two additional interviews were conducted to confirm the stability of the findings.

2. Sampling bias is a significant limitation, as participants were recruited through community-based organizations and are likely more engaged with HIV services than the broader MSM population.

Explained using diffusion of innovation theory. In the discussion we added:

Formatted: Font: 11 pt

Formatted: Line spacing: Multiple 1.15 li

Formatted: Font: (Default) Times New Roman, 11 pt

Formatted: Font: 11 pt

Formatted: Font: (Default) Times New Roman, 11 pt

According to the social network model of diffusion of innovation, opinion leaders help uptake of innovation or intervention. Our study highlights that the MSM were first promoted for use of HIVST in the DIC, followed by ordering through the digital platform

Formatted: Font: 11 pt

Also theme 1.3, states peer-driven diffusion of testing.

This even though sampling bias is a limitation, which is acknowleged, newer interventions are taken by early adopters who have health seeking behaviors. However the change is perpetuated to later adopters from them

Formatted: Font: (Default) Times New Roman, 11 pt

3. The sample size is small and lacks diversity (e.g., mostly educated, urban participants), limiting generalizability.

Acknowledged and explain using diffusion of innovation theory as above

4. The methodological description lacks detail, particularly regarding coding strategy, development of themes, and how discrepancies between coders were resolved.

Readdressed and revised, see section data analysis, also pasted below

The transcripts were imported into NVIVO version 14.0.[12] Analysis was driven by an inductive thematic analysis followed by post-hoc interpretation using Consolidated Framework for Implementation Research (CFIR) as an analytic lens. CFIR is a key framework used in implementation science to evaluate complex interventions, including public health interventions.[13–15]

Formatted: Font: 11 pt

Formatted: Font: 11 pt

Formatted: Font: 11 pt

Formatted: Font: 11 pt

Formatted: Font: 11 pt

The data transcript was available to two independent researchers. After becoming familiar with the data, the researcher independently conducted description- and interpretation-focused coding. In the description-focused coding technique, the researcher assigns a code that captures the participant's experience without inference. In interpretation-focused coding, the researcher assigns codes to capture the underlying processes of the experience. Any discrepancy in coding was settled by mutual consensus and deliberation guided by a third researcher. After coding had been completed, codes were sorted into clusters using an individual-based sorting strategy. Codes were sorted into clusters based on similarities and differences. Final themes were arrived at to answer the research questions. For this study, the key research questions were: 1) Is the current digital HIVST an acceptable and useful intervention for the MSM community? 2) What are the opportunities to adapt digital HIVST services to make them more effective for improving HIV testing among MSM?

In the final step, themes were interpreted onto CFIR domains and constructs. Where appropriate, themes were mapped on more than one CFIR domain to reflect their complexity and cross-cutting nature.

Formatted: Font: (Default) Times New Roman, 11 pt

5. The analysis is largely descriptive, with limited interpretive depth or engagement with relevant theoretical frameworks (e.g., stigma, digital health adoption).

Readdressed as much as possible, rewritten analysis and reduced themes from 5 to 3 to reduce overlap. Also used CFIR framework to add interpretive angle to the study. See table III, as well as re-analyzed results

6. There is conceptual overlap between themes (e.g., stigma, confidentiality, and acceptability), which weakens the clarity and structure of the findings.

Reduced and clubbed stigma themes for clarity, acceptance is covered in theme 1 under same heading

7. The manuscript does not sufficiently explore contradictory perspectives (e.g., home testing as both private and potentially risky).

Addressed where theme 2 explain how stigma is displaced from CBO settings to home settings where ordering HIVST at home.

Theme 2. Digital platform users trade off between stigma at CBOs vs at home for HIV testing

8. The discussion section lacks depth, particularly in translating findings into actionable policy or programmatic implications within the Pakistani context.

Readdressed, and rewritten with subheading. We divided discussion in various headings,

Programmatic implications are covered in two subheadings

Digital HIVST dispensation, stigma and mental health support

and

Digital platform integration

9. Ethical considerations are underdeveloped, given the criminalization and stigmatization of MSM, especially in relation to digital privacy and potential risks of home delivery.

Theme 2 caters this.

10. The data availability statement ("available on request") may not meet journal requirements and needs clarification or revision.

We can provide transcripts based on reasonable request, the data is otherwise in mansuscript. This adheres to journal policy

Minor Comments

Formatted: Font: (Default) Times New Roman, 11 pt, Pattern: Clear

Formatted: Font: (Default) Times New Roman, 11 pt

Formatted: Font: 11 pt

Formatted: Font: (Default) Times New Roman, 11 pt

Formatted: Font: 11 pt, Not Bold

Formatted: Justified, Space Before: 6 pt, Line spacing: Multiple 1.15 li

Formatted: Font: (Default) Times New Roman, 11 pt, Italic, Font color: Black

Formatted: Font: (Default) Times New Roman, 11 pt

Formatted: Line spacing: Multiple 1.15 li

1. Several grammatical and language issues are present; the manuscript would benefit from professional editing for clarity and flow.

Thank you, have taken help from grammarly and reviewed manuscript again

2. There is repetition of participant quotes and ideas, particularly around stigma and confidentiality.

All quotes are unique

3. Table II contains an error ("mean age… 24,500 PKR"), indicating incorrect units and poor formatting.

Corrected

4. Some references are incorrectly formatted or incomplete, and links (e.g., UNAIDS citation) appear inaccurate.

Corrected, chose Vancouver style which is accepted by PLOS journals.

5. The ethical statement is repeated, suggesting duplication that should be corrected.

Deleted from title page

6. Terminology such as MSM, MSW, and bisexual is noted as confusing by participants, but not clearly defined in the manuscript.

I think they are largely in the literature but here reflect implementer centered lanugage which is explictly mentioned in subtheme 3.2. It needs to be changed in for optimization of platform

Thus, the content needs to be centered around user-friendly language, which shall improve trust. In addition, the collection of personal information should be minimized to encourage service use.

7. The relationship between the two digital platforms (Sehat Dost and QuickRes) is not clearly explained and may confuse readers.

We have rephrased this section and moved it in the methods

8. Transitions between sections and themes are abrupt, affecting overall readability.

Readdressed, now themes are accpetance, context and recommendations (theme1, 2 and 3 in that order)

9. The manuscript would benefit from clearer subheadings in the discussion to improve structure.

Added

10. Some sentences are awkwardly phrased or unclear, requiring refinement for precision and readability.

Revisited and revisions are made

Description of the Digital HIVST model

Formatted: Font: (Default) Times New Roman, 11 pt, Not Bold

Formatted: Font: (Default) Times New Roman, 11 pt

Formatted: Font: (Default) Times New Roman, 11 pt, Not Bold

Formatted: Font: (Default) Times New Roman, 11 pt, No underline

Formatted: Font: 11 pt

Formatted: Font: (Default) Times New Roman, 11 pt

Formatted: Font: (Default) Times New Roman, 11 pt, Not Bold

Formatted: Font: (Default) Times New Roman, 11 pt

Formatted: Font: (Default) Times New Roman, 11 pt, Not Bold

Formatted: Font: (Default) Times New Roman, 11 pt

Formatted: Font: (Default) Times New Roman, 11 pt, Not Bold

Formatted: Font: (Default) Times New Roman, 11 pt

Formatted: Font: 11 pt, Not Bold

Two digital platforms were developed and linked: one providing information on HIV prevention, treatment, and facilities (Sehat Dost), and the other for ordering HIVST kits (QuickRes). The interface of Sehat Dost (www.sehatdost.com) has seven key modules. The last module facilitates digital HIVST dispensation. Once someone clicks on HIVST dispensation, it lands on a webpage of QuickRes (www.quickres.org). QuickRes is a global platform used to make appointments and reservations for HIV services.[11] MSM can place an order for oral fluid-based HIVST on QuickRes. The order is placed, and the venue and time for collecting HIVST are selected; at the given time and venue, the CBO staff provides HIVST kits through a digital service or staff.

To generate demand for HIV services, advertisements are made in CBO drop-in centers (DIC) as well as messaging on WhatsApp, which includes a package of two videos, one focusing on how to perform HIVST and the other on how to use digital platforms. The demand generation faces significant challenges, as CBOs have to work in relative secrecy given the legal and social atmosphere of the country.

Reviewer 1 comments

The ethical statement need to explain how you main the ethical issues during the interview conduct with the ethical approval. Institutional approval does not give you all rights to conduct the interview, study participants also have some rights. How you ensure this? Do you get consent from the study participant?)

Written informed consent was obtained before data collection. (see section Interviews and the data collection process)

1. Please specify the study settings clearly?

Please see methods section, study was conducted in Dostana and Hamraz, two of MSM CBOs in Lahore and Karachi.

2. Please specify the study design clearly?

Mentioned in methods section

This qualitative exploratory study was conducted from May 2025 to July 2025 in Lahore and Karachi.

3. Who collect the data? e.g, their background, related experience? (Please see reflexivity statement)

The lead investigator has been working in HIV for nearly seven years, whereas XX has been working for nearly two decades. YY has been involved in community research and various qualitative projects, whereas XX has worked at the national level in HIV response with various agencies. The lead investigator is a community advocate. Throughout the research, reflexive discussions were undertaken to examine the impact of researchers' backgrounds and experiences on research. The practice of independent coding by two researchers, followed by iterative sorting, helped bracket individual perspectives of the researchers and enhance methodological rigor. Findings were supported by verbatim from the transcripts to ensure credibility and transparency.YY has worked in public health for over two decades, providing an overview of the data coding and analysis processes.

Formatted: Font: 11 pt

Formatted: Font: 11 pt

Formatted: Font: 11 pt

Formatted: Font: 11 pt

Formatted: Font: 11 pt

Formatted: Font: (Default) Times New Roman, 11 pt, Italic

Formatted: Font: 11 pt, Not Bold

Formatted: Font: 11 pt

Formatted: Justified, Space Before: 6 pt, Line spacing: Multiple 1.15 li

Formatted: Font: 11 pt, Not Bold

Formatted: Font: (Default) Times New Roman

Formatted: Line spacing: Multiple 1.15 li

Formatted: Font: 11 pt, Not Bold

Formatted: Font: (Default) Times New Roman

Formatted: Font: 11 pt

Formatted: Justified, Space Before: 6 pt, Line spacing: 1.5 lines

Formatted: Font: 11 pt, Not Bold

Formatted: Line spacing: Multiple 1.15 li

Formatted: Font: 11 pt

Formatted: Font: 11 pt, Not Bold

4. Why the study participant give time to you?

They have volunteered, based on repute of investigators as the lead was also from community, no renumeration was provided

5. Where interview were conducted? is there any ethical issues related to data collection and manage for interview? We had no issue in data collection process, as CBOs are safe spaces.

6. Duration of the interviews?

For 1-6 please see section Interviews and data collection process)

Once eligible participants were recruited, they were given the option of being interviewed either individually (in-depth interview) or in a focus group discussion (FGD). Written informed consent was obtained before data collection. Semi-structured interviews were conducted face-to-face by a lead investigator using an interview guide. Interviews were conducted on CBO premises in a separate room, ensuring confidentiality. Interviews lasted from 25 minutes for IDIs to over an hour for FGDs. Remuneration was not provided to the participants. Participants were asked about their experience with digital platforms for HIVST, how it compares with in-person HIV testing at the CBO, perceived barriers, and benefits of digital HIVST services.

7. How you control the interview session need to explain? if any unintended event happened as MSM is unauthorized?

There were no unintended events. Service provision is legal in Pakistan. But having sex with men is illegal.

Please mention what specific sampling procedure you used? like snowbal?

They were identified by outreach workers based on personal contacts. As mentioned above

How many researcher coded the data?

Two researchers coded the data see data analysis section also pasted below:

The data transcript was available to two independent researchers. After becoming familiar with the data, the researcher independently conducted description- and interpretation-focused coding. In the description-focused coding technique, the researcher assigns a code that captures the participant's experience without inference. In interpretation-focused coding, the researcher assigns codes to capture the underlying processes of the experience. Any discrepancy in coding was settled by mutual consensus and deliberation guided by a third researcher. After coding had been completed, codes were sorted into clusters using an individual-based sorting strategy. Codes were sorted into clusters based on similarities and differences. Final themes were arrived at to answer the research questions. For this study, the key research questions were: 1) Is the current digital HIVST an acceptable and useful intervention for the MSM community? 2) What are the opportunities to adapt digital HIVST services to make them more effective for improving HIV testing among MSM?

How you enssure about the true meaning of the data?

Formatted: Font: (Default) Times New Roman

Formatted: Line spacing: Multiple 1.15 li

Formatted: Font: 11 pt, Not Bold

Formatted: Font: 11 pt

Formatted: Justified, Space Before: 6 pt, Line spacing: Multiple 1.15 li

Formatted: Font: 11 pt, Not Bold, Font color: Black

Formatted: Font: 11 pt, Not Bold

Formatted: Line spacing: Multiple 1.15 li

Formatted: Font: 11 pt

Formatted: Font: 11 pt, Not Bold

Formatted: Font: 11 pt

Formatted: Justified, Space Before: 6 pt, Line spacing: Multiple 1.15 li

Formatted: Font: 11 pt, Not Bold, Font color: Black

Formatted: Font: 11 pt, Not Bold

Formatted: Font: (Default) Times New Roman

Two independent coders, followed by deliberations between teams and mapping on CFIR domains helped interpret the data

How you address the unusal data during analysis?

I am not clear what this meant

The service recipient did not tested HIV earlier due to stigma? if so, then that would be come first that experience help to use the on-line HIVST service?

They have tested but perceived stigma deterring them in some ways. So they prefer online, if you look at Table 1, it states index test was in DIC

That indicate that DIC previously does not care about the privacy issue of MSM? It is a big claim!

Perceived stigma differs from experienced stigma, within community 'internalized stigma' exists and is well reported includin minority stress theory

In online, it does not require any personal detail? e.g, name, address, mobile number etc. Then how MSM receive HIVST?

The online system does require, it is just that they don't have to reveal full name, face etc

In above findings seems conflicting with this findings! (stigma at home)

We have explained this in theme 2 more. It is the stigma displacing

Why the environment is friendly and comfortable? (in CBOs)

It is more friendly because all CBOs employ MSM as workers, the stigma is less than general community setting but internalized stigma does exists, stigma thus operates differently depending on the context

That indicate there have chances of privacy breach? (in home testing)

Yes

It would be good to compare the findings of in-person and digital use to get the clear picture?

We have rewritten discussion focusing on policy implications and using CFIR frame

In qualitative study does not have any selction bias if you select the study participants based on your criteria? you have mention why this participant you selected? and the term selection bias need to remove and need to mention specific limitation.

Removed and limitations now read as

The current study, however, has several limitations. First, it explored the qualitative experiences of the participants. The participants were interviewed by a researcher who was a former manager at the CBO; this may induce reporting and desirability bias, where participants only reported favorable themes. The participants were from two cities in Pakistan; thus, their experience may not be generalizable to all MSM residing in the country. Furthermore, we only used CFIR for post-hoc inductive analysis. CFIR is a

Formatted: Line spacing: Multiple 1.15 li

Formatted: Font: 11 pt, Not Bold

Formatted: Font: (Default) Times New Roman

Formatted: Line spacing: Multiple 1.15 li

Formatted: Font: 11 pt, Not Bold

Formatted: Font: (Default) Times New Roman

Formatted: Line spacing: Multiple 1.15 li

Formatted: Font: 11 pt, Not Bold

Formatted: Font: (Default) Times New Roman

Formatted: Line spacing: Multiple 1.15 li

Formatted: Font: 11 pt, Not Bold

Formatted: Font: (Default) Times New Roman

Formatted: Line spacing: Multiple 1.15 li

Formatted: Font: 11 pt, Not Bold

Formatted: Font: (Default) Times New Roman

Formatted: Line spacing: Multiple 1.15 li

Formatted: Font: 11 pt, Not Bold

Formatted: Font: (Default) Times New Roman

Formatted: Line spacing: Multiple 1.15 li

Formatted: Font: 11 pt, Not Bold

Formatted: Font: (Default) Times New Roman

Formatted: Line spacing: Multiple 1.15 li

Formatted: Font: 11 pt, Not Bold

Formatted: Font: (Default) Times New Roman

Formatted: Line spacing: Multiple 1.15 li

Formatted: Font: 11 pt, Not Bold

Formatted: Font: (Default) Times New Roman

Formatted: Font: 11 pt

Formatted: Justified, Space Before: 6 pt, Line spacing: 1.5 lines

comprehensive tool for appraisal and planning of intervention. Many of the CFIR domains are related to implementer, organizational, and donor perspectives. Our study, thus, uses CFIR as a lens for capturing user perspectives while not evaluating the intervention from the viewpoint of other stakeholders. Nonetheless, the study has several strengths allowing for greater understanding of how outer settings influence HIVST digital dispensation and how the innovation can be adapted for improved efficiency.

Is this? then need to explain that problem analysis more in details (platform navigation)

We have elaborated it in following section

Digital platform integration

While information on these is available on the Sehat Dost platform, participants highlighted that the presence of a separate platform for HIVST dispensation (Quickres) may be cumbersome to use. Although the current model of intervention relies on digital dispensation and mailing of HIVST, participants stated that they shared the link to the digital dispensation platform with other community members. It should be noted that this method may be used to engage MSM who cannot be reached by conventional outreach by CBOs. These themes illustrate the need for innovation and adaptability. In addition, the users also highlighted provider-centered language and data capture that need to be addressed. These recommendations are important, given that innovation is inherently diffusing to late adopters, who may be less motivated to use the service.[27] Providing client-centered and easy usability of the platform will improve user satisfaction and increase use of the platform.

They also mention there have issue in social media for opened post! please maintain the findings and recommendation accordingly. (Social media demand generation)

Please see theme 3.4

Subtheme 3.4. Using diverse demand generation through in-person interactions and digital means

The participants recommended the need for a multipronged digital demand generation. These include outreach at dating applications, as well as social media. However, digital demand generation should not replace human interaction. In fact, interpersonal interaction can complement digital demand generation, ensuring uptake of services. One participant stated that

We can't send it through just by sharing the link, as it is sensitive. You can only share it after a deep conversation. Only then will the next person understand it and be able to use it positively. You can also give advertisements on Grindr and other dating applications. FGD1

Formatted: Font: 11 pt, Not Bold, Font color: Black

Formatted: Font: 11 pt, Not Bold

Formatted: Font: (Default) Times New Roman

Formatted: Font: 11 pt

Formatted: Justified, Space Before: 6 pt, Line spacing: 1.5 lines

Formatted: Font: 11 pt

Formatted: Font: 11 pt

Formatted: Font: 11 pt, Not Bold

While some people have stressed the use of dating applications for advertising, others have stated the use of social media applications for the demand generation of HIV services and self-testing.

Not everyone uses Grindr. For example, I don't use Grindr. Most people use Instagram and Facebook (for hookups and dates). Even within the gay community, not everyone is on Grindr. MSM7, 23 years old, part-time employee, 14 years of education

One participant stated that there should be group sessions to complement digital messaging, and stated that people may take digital messaging for granted.

There should be more awareness sessions in the CBOs, too. They should hold regular sessions so people can learn. Because most people don't read everything on the app. They either skip through or just don't bother going into full detail. FGD2

These reflections from the participants highlight the need for multipronged and well-connected digital outreach and demand generation, leveraging social media and dating applications, with interpersonal interactions, individually or as a group, rather than just sharing a platform link.

Formatted: Font: 11 pt, Not Bold

Formatted: Line spacing: Multiple 1.15 li
