# Supplementary material for: Evaluating the digital dispensation of HIV self-testing (HIVST) among men who have sex with men (MSM) in Pakistan: Seeing through the Consolidated Framework for Implementation Research (CFIR) lens
Source: PLOS Glob Public Health. 2026 Sep 11;6(9):e0005994. doi: 10.1371/journal.pgph.0005994 (PMC13567783; doi:10.1371/journal.pgph.0005994)
Supplement: Supplementary file 4 [file pgph.0005994.s004.docx]

The Editor,

PLOS Global Public Health,

22 June 2026

Dear Editor,

Thank you for sharing the reviewers' comments for the manuscript. I am making pointwise response to the concerns raised below.

Reviewer #2: Summary
This study qualitatively assesses a digital HIV self-testing (HIVST) intervention among men who have sex with men (MSM) in Pakistan, focusing on user experiences with an online platform that allows ordering of OraQuick self-test kits for home delivery. Using 12 in-depth interviews and two focus group discussions, the authors found that digital HIVST is generally acceptable and preferred because it reduces stigma, enhances confidentiality, and offers convenience compared to facility-based testing. Participants also noted the importance of peer sharing and digital platforms in potentially increasing HIV testing uptake within the MSM community. However, they identified key barriers, including concerns about confidentiality at home, ongoing stigma, challenges in navigating the platform, and fragmentation between different digital services. Participants further emphasized the need for a more comprehensive service package, including condoms, lubricants, and online counseling, as well as improved demand generation through social media and dating apps. Overall, the study indicates that while digital HIVST holds great potential in this context, optimizing platform design, service integration, and delivery strategies is necessary for effective scale-up.


Major Comments
1. The study does not clearly demonstrate whether data saturation was achieved, which is essential for qualitative rigor.

Data collection and preliminary analysis were conducted concurrently. Saturation was considered achieved when no new codes or concepts emerged from successive interviews. Saturation was reached after ten IDIs, following which two additional interviews were conducted to confirm the stability of the findings.

2. Sampling bias is a significant limitation, as participants were recruited through community-based organizations and are likely more engaged with HIV services than the broader MSM population.

Explained using diffcusion of innovation theory

3. The sample size is small and lacks diversity (e.g., mostly educated, urban participants), limiting generalizability.

Acknowledged and explain using diffusion of innovation theory
4. The methodological description lacks detail, particularly regarding coding strategy, development of themes, and how discrepancies between coders were resolved.

Readdressed and revised
5. The analysis is largely descriptive, with limited interpretive depth or engagement with relevant theoretical frameworks (e.g., stigma, digital health adoption).

Readdressed as muhc as possible, rewritten analysis and reduced themes to 5
6. There is conceptual overlap between themes (e.g., stigma, confidentiality, and acceptability), which weakens the clarity and structure of the findings.

Reduced and clubeed stigma themes for clarity
7. The manuscript does not sufficiently explore contradictory perspectives (e.g., home testing as both private and potentially risky).

Addressed

8. The discussion section lacks depth, particularly in translating findings into actionable policy or programmatic implications within the Pakistani context.

Readdressed, and rewritten with subheading
9. Ethical considerations are underdeveloped, given the criminalization and stigmatization of MSM, especially in relation to digital privacy and potential risks of home delivery.

Theme 2 caters this
10. The data availability statement (“available on request”) may not meet journal requirements and needs clarification or revision.


Minor Comments
1. Several grammatical and language issues are present; the manuscript would benefit from professional editing for clarity and flow.

Thank you, have taken help from grammarly and reviewed manuscript again
**2. There is repetition of participant quotes and ideas, particularly around stigma and confidentiality.**

3. Table II contains an error (“mean age… 24,500 PKR”), indicating incorrect units and poor formatting.

Corrected
4. Some references are incorrectly formatted or incomplete, and links (e.g., UNAIDS citation) appear inaccurate.

Corrected, chose Vancouver style which is accepted by PLOS journals.
5. The ethical statement is repeated, suggesting duplication that should be corrected.

Deleted from title page
**6. Terminology such as MSM, MSW, and bisexual is noted as confusing by participants, but not clearly defined in the manuscript.**

**I think they are largely in the literature but here reflect implementer centered lanugage which is explictly mentioned
7. The relationship between the two digital platforms (Sehat Dost and QuickRes) is not clearly explained and may confuse readers.**

We have rephrased this section

**8. Transitions between sections and themes are abrupt, affecting overall readability.**

**Readdressed, now themes are accpetance, context and recommendations (theme1, 2 and 3 in that order)**

**9. The manuscript would benefit from clearer subheadings in the discussion to improve structure.**

Added
10. Some sentences are awkwardly phrased or unclear, requiring refinement for precision and readability.

Revisited and revisions are made
