# Supplementary material for: Evaluating the digital dispensation of HIV self-testing (HIVST) among men who have sex with men (MSM) in Pakistan: Seeing through the Consolidated Framework for Implementation Research (CFIR) lens
Source: PLOS Glob Public Health. 2026 Sep 11;6(9):e0005994. doi: 10.1371/journal.pgph.0005994 (PMC13567783; doi:10.1371/journal.pgph.0005994)
Supplement: Supplementary file 5 [file pgph.0005994.s006.docx]

The Editor,

PLOS Global Public Health,

22 June 2026

Dear Editor,

Thank you for sharing the reviewers' comments for the manuscript. I am making pointwise response to the concerns raised below.

Reviewer #2: Summary
This study qualitatively assesses a digital HIV self-testing (HIVST) intervention among men who have sex with men (MSM) in Pakistan, focusing on user experiences with an online platform that allows ordering of OraQuick self-test kits for home delivery. Using 12 in-depth interviews and two focus group discussions, the authors found that digital HIVST is generally acceptable and preferred because it reduces stigma, enhances confidentiality, and offers convenience compared to facility-based testing. Participants also noted the importance of peer sharing and digital platforms in potentially increasing HIV testing uptake within the MSM community. However, they identified key barriers, including concerns about confidentiality at home, ongoing stigma, challenges in navigating the platform, and fragmentation between different digital services. Participants further emphasized the need for a more comprehensive service package, including condoms, lubricants, and online counseling, as well as improved demand generation through social media and dating apps. Overall, the study indicates that while digital HIVST holds great potential in this context, optimizing platform design, service integration, and delivery strategies is necessary for effective scale-up.


Major Comments
1. The study does not clearly demonstrate whether data saturation was achieved, which is essential for qualitative rigor.

Data collection and preliminary analysis were conducted concurrently. Saturation was considered achieved when no new codes or concepts emerged from successive interviews. Saturation was reached after ten IDIs, following which two additional interviews were conducted to confirm the stability of the findings.

2. Sampling bias is a significant limitation, as participants were recruited through community-based organizations and are likely more engaged with HIV services than the broader MSM population.

Explained using diffusion of innovation theory. In the discussion we added:

According to the social network model of diffusion of innovation, opinion leaders help uptake of innovation or intervention. Our study highlights that the MSM were first promoted for use of HIVST in the DIC, followed by ordering through the digital platform

Also theme 1.3, states peer-driven diffusion of testing.

This even though sampling bias is a limitation, which is acknowleged, newer interventions are taken by early adopters who have health seeking behaviors. However the change is perpetuated to later adopters from them

3. The sample size is small and lacks diversity (e.g., mostly educated, urban participants), limiting generalizability.

Acknowledged and explain using diffusion of innovation theory as above

4. The methodological description lacks detail, particularly regarding coding strategy, development of themes, and how discrepancies between coders were resolved.

Readdressed and revised, see section data analysis, also pasted below

The transcripts were imported into NVIVO version 14.0.[12] Analysis was driven by an inductive thematic analysis followed by post-hoc interpretation using Consolidated Framework for Implementation Research (CFIR) as an analytic lens. CFIR is a key framework used in implementation science to evaluate complex interventions, including public health interventions.[13–15]

The data transcript was available to two independent researchers. After becoming familiar with the data, the researcher independently conducted description- and interpretation-focused coding. In the description-focused coding technique, the researcher assigns a code that captures the participant's experience without inference. In interpretation-focused coding, the researcher assigns codes to capture the underlying processes of the experience. Any discrepancy in coding was settled by mutual consensus and deliberation guided by a third researcher. After coding had been completed, codes were sorted into clusters using an individual-based sorting strategy. Codes were sorted into clusters based on similarities and differences. Final themes were arrived at to answer the research questions. For this study, the key research questions were: 1) Is the current digital HIVST an acceptable and useful intervention for the MSM community? 2) What are the opportunities to adapt digital HIVST services to make them more effective for improving HIV testing among MSM?

In the final step, themes were interpreted onto CFIR domains and constructs. Where appropriate, themes were mapped on more than one CFIR domain to reflect their complexity and cross-cutting nature.

5. The analysis is largely descriptive, with limited interpretive depth or engagement with relevant theoretical frameworks (e.g., stigma, digital health adoption).

Readdressed as much as possible, rewritten analysis and reduced themes from 5 to 3 to reduce overlap. Also used CFIR framework to add interpretive angle to the study. See table III, as well as re-analyzed results

6. There is conceptual overlap between themes (e.g., stigma, confidentiality, and acceptability), which weakens the clarity and structure of the findings.

Reduced and clubbed stigma themes for clarity, acceptance is covered in theme 1 under same heading
7. The manuscript does not sufficiently explore contradictory perspectives (e.g., home testing as both private and potentially risky).

Addressed where theme 2 explain how stigma is displaced from CBO settings to home settings where ordering HIVST at home.

# Theme 2. Digital platform users trade off between stigma at CBOs vs at home for HIV testing

8. The discussion section lacks depth, particularly in translating findings into actionable policy or programmatic implications within the Pakistani context.

Readdressed, and rewritten with subheading. We divided discussion in various headings,

Programmatic implications are covered in two subheadings

*Digital HIVST dispensation, stigma and mental health support*

*and*

*Digital platform integration*

9. Ethical considerations are underdeveloped, given the criminalization and stigmatization of MSM, especially in relation to digital privacy and potential risks of home delivery.

Theme 2 caters this.
10. The data availability statement (“available on request”) may not meet journal requirements and needs clarification or revision.

We can provide transcripts based on reasonable request, the data is otherwise in mansuscript. This adheres to journal policy
Minor Comments
1. Several grammatical and language issues are present; the manuscript would benefit from professional editing for clarity and flow.

Thank you, have taken help from grammarly and reviewed manuscript again
2. There is repetition of participant quotes and ideas, particularly around stigma and confidentiality.

All quotes are unique

3. Table II contains an error (“mean age… 24,500 PKR”), indicating incorrect units and poor formatting.

Corrected
4. Some references are incorrectly formatted or incomplete, and links (e.g., UNAIDS citation) appear inaccurate.

Corrected, chose Vancouver style which is accepted by PLOS journals.
5. The ethical statement is repeated, suggesting duplication that should be corrected.

Deleted from title page
6. Terminology such as MSM, MSW, and bisexual is noted as confusing by participants, but not clearly defined in the manuscript.

I think they are largely in the literature but here reflect implementer centered lanugage which is explictly mentioned in subtheme 3.2. It needs to be changed in for optimization of platform

Thus, the content needs to be centered around user-friendly language, which shall improve trust. In addition, the collection of personal information should be minimized to encourage service use.

7. The relationship between the two digital platforms (Sehat Dost and QuickRes) is not clearly explained and may confuse readers.

We have rephrased this section and moved it in the methods

8. Transitions between sections and themes are abrupt, affecting overall readability.

Readdressed, now themes are accpetance, context and recommendations (theme1, 2 and 3 in that order)

9. The manuscript would benefit from clearer subheadings in the discussion to improve structure.

Added
10. Some sentences are awkwardly phrased or unclear, requiring refinement for precision and readability.

Revisited and revisions are made

Description of the Digital HIVST model

Two digital platforms were developed and linked: one providing information on HIV prevention, treatment, and facilities (Sehat Dost), and the other for ordering HIVST kits (QuickRes). The interface of Sehat Dost (www.sehatdost.com) has seven key modules. The last module facilitates digital HIVST dispensation. Once someone clicks on HIVST dispensation, it lands on a webpage of QuickRes ([www.quickres.org](https://quickres.org)). QuickRes is a global platform used to make appointments and reservations for HIV services.[11] MSM can place an order for oral fluid-based HIVST on QuickRes. The order is placed, and the venue and time for collecting HIVST are selected; at the given time and venue, the CBO staff provides HIVST kits through a digital service or staff.

To generate demand for HIV services, advertisements are made in CBO drop-in centers (DIC) as well as messaging on WhatsApp, which includes a package of two videos, one focusing on how to perform HIVST and the other on how to use digital platforms. The demand generation faces significant challenges, as CBOs have to work in relative secrecy given the legal and social atmosphere of the country.

Reviewer 1 comments

The ethical statement need to explain how you main the ethical issues during the interview conduct with the ethical approval. Institutional approval does not give you all rights to conduct the interview, study participants also have some rights. How you ensure this? Do you get consent from the study participant?)

Written informed consent was obtained before data collection. (see section Interviews and the data collection process)

1. Please specify the study settings clearly?

Please see methods section, study was conducted in Dostana and Hamraz, two of MSM CBOs in Lahore and Karachi.

2. Please specify the study design clearly?

Mentioned in methods section

This qualitative exploratory study was conducted from May 2025 to July 2025 in Lahore and Karachi.

3. Who collect the data? e.g, their background, related experience? (Please see reflexivity statement)The lead investigator has been working in HIV for nearly seven years, whereas XX has been working for nearly two decades. YY has been involved in community research and various qualitative projects, whereas XX has worked at the national level in HIV response with various agencies. The lead investigator is a community advocate. Throughout the research, reflexive discussions were undertaken to examine the impact of researchers' backgrounds and experiences on research. The practice of independent coding by two researchers, followed by iterative sorting, helped bracket individual perspectives of the researchers and enhance methodological rigor. Findings were supported by verbatim from the transcripts to ensure credibility and transparency.YY has worked in public health for over two decades, providing an overview of the data coding and analysis processes.

4. Why the study participant give time to you?

They have volunteered, based on repute of investigators as the lead was also from community, no renumeration was provided

5. Where interview were conducted? is there any ethical issues related to data collection and manage for interview? We had no issue in data collection process, as CBOs are safe spaces.

6. Duration of the interviews?

For 1-6 please see section Interviews and data collection process)

Once eligible participants were recruited, they were given the option of being interviewed either individually (in-depth interview) or in a focus group discussion (FGD). Written informed consent was obtained before data collection. Semi-structured interviews were conducted face-to-face by a lead investigator using an interview guide. Interviews were conducted on CBO premises in a separate room, ensuring confidentiality. Interviews lasted from 25 minutes for IDIs to over an hour for FGDs. Remuneration was not provided to the participants. Participants were asked about their experience with digital platforms for HIVST, how it compares with in-person HIV testing at the CBO, perceived barriers, and benefits of digital HIVST services.

7. How you control the interview session need to explain? if any unintended event happened as MSM is unauthorized?

There were no unintended events. Service provision is legal in Pakistan. But having sex with men is illegal.

Please mention what specific sampling procedure you used? like snowbal?

They were identified by outreach workers based on personal contacts. As mentioned above

How many researcher coded the data?

Two researchers coded the data see data analysis section also pasted below:

The data transcript was available to two independent researchers. After becoming familiar with the data, the researcher independently conducted description- and interpretation-focused coding. In the description-focused coding technique, the researcher assigns a code that captures the participant's experience without inference. In interpretation-focused coding, the researcher assigns codes to capture the underlying processes of the experience. Any discrepancy in coding was settled by mutual consensus and deliberation guided by a third researcher. After coding had been completed, codes were sorted into clusters using an individual-based sorting strategy. Codes were sorted into clusters based on similarities and differences. Final themes were arrived at to answer the research questions. For this study, the key research questions were: 1) Is the current digital HIVST an acceptable and useful intervention for the MSM community? 2) What are the opportunities to adapt digital HIVST services to make them more effective for improving HIV testing among MSM?

How you enssure about the true meaning of the data?

Two independent coders, followed by deliberations between teams and mapping on CFIR domains helped interpret the data

How you address the unusal data during analysis?

I am not clear what this meant

The service recipient did not tested HIV earlier due to stigma? if so, then that would be come first that experience help to use the on-line HIVST service?

They have tested but perceived stigma deterring them in some ways. So they prefer online, if you look at Table 1, it states index test was in DIC

That indicate that DIC previously does not care about the privacy issue of MSM? It is a big claim!

Perceived stigma differs from experienced stigma, within community ‘internalized stigma’ exists and is well reported includin minority stress theory

In online, it does not require any personal detail? e.g, name, address, mobile number etc. Then how MSM receive HIVST?

The online system does require, it is just that they don’t have to reveal full name, face etc

In above findings seems conflicting with this findings! (stigma at home)

We have explained this in theme 2 more. It is the stigma displacing

Why the environment is friendly and comfortable? (in CBOs)

It is more friendly because all CBOs employ MSM as workers, the stigma is less than general community setting but internalized stigma does exists, stigma thus operates differently depending on the context

That indicate there have chances of privacy breach? (in home testing)

Yes

It would be good to compare the findings of in-person and digital use to get the clear picture?

We have rewritten discussion focusing on policy implications and using CFIR frame

In qualitative study does not have any selction bias if you select the study participants based on your criteria? you have mention why this participant you selected? and the term selection bias need to remove and need to mention specific limitation.

Removed and limitations now read as

The current study, however, has several limitations. First, it explored the qualitative experiences of the participants. The participants were interviewed by a researcher who was a former manager at the CBO; this may induce reporting and desirability bias, where participants only reported favorable themes. The participants were from two cities in Pakistan; thus, their experience may not be generalizable to all MSM residing in the country. Furthermore, we only used CFIR for post-hoc inductive analysis. CFIR is a comprehensive tool for appraisal and planning of intervention. Many of the CFIR domains are related to implementer, organizational, and donor perspectives. Our study, thus, uses CFIR as a lens for capturing user perspectives while not evaluating the intervention from the viewpoint of other stakeholders. Nonetheless, the study has several strengths allowing for greater understanding of how outer settings influence HIVST digital dispensation and how the innovation can be adapted for improved efficiency.

Is this? then need to explain that problem analysis more in details (platform navigation)

We have elaborated it in following section

***Digital platform integration***

While information on these is available on the Sehat Dost platform, participants highlighted that the presence of a separate platform for HIVST dispensation (Quickres) may be cumbersome to use. Although the current model of intervention relies on digital dispensation and mailing of HIVST, participants stated that they shared the link to the digital dispensation platform with other community members. It should be noted that this method may be used to engage MSM who cannot be reached by conventional outreach by CBOs. These themes illustrate the need for innovation and adaptability. In addition, the users also highlighted provider-centered language and data capture that need to be addressed. These recommendations are important, given that innovation is inherently diffusing to late adopters, who may be less motivated to use the service.[27] Providing client-centered and easy usability of the platform will improve user satisfaction and increase use of the platform.

They also mention there have issue in social media for opened post! please maintain the findings and recommendation accordingly. (Social media demand generation)

Please see theme 3.4

## Subtheme 3.4. Using diverse demand generation through in-person interactions and digital means

The participants recommended the need for a multipronged digital demand generation. These include outreach at dating applications, as well as social media. However, digital demand generation should not replace human interaction. In fact, interpersonal interaction can complement digital demand generation, ensuring uptake of services. One participant stated that

*We can't send it through just by sharing the link, as it is sensitive. You can only share it after a deep conversation. Only then will the next person understand it and be able to use it positively. You can also give advertisements on Grindr and other dating applications*. *FGD1*

While some people have stressed the use of dating applications for advertising, others have stated the use of social media applications for the demand generation of HIV services and self-testing.

*Not everyone uses Grindr. For example, I don’t use Grindr. Most people use Instagram and Facebook (for hookups and dates). Even within the gay community, not everyone is on Grindr. MSM7, 23 years old, part-time employee, 14 years of education*

One participant stated that there should be group sessions to complement digital messaging, and stated that people may take digital messaging for granted.

*There should be more awareness sessions in the CBOs, too. They should hold regular sessions so people can learn. Because most people don’t read everything on the app. They either skip through or just don’t bother going into full detail. FGD2*

These reflections from the participants highlight the need for multipronged and well-connected digital outreach and demand generation, leveraging social media and dating applications, with interpersonal interactions, individually or as a group, rather than just sharing a platform link.
