# Supplementary material for: Evaluating the digital dispensation of HIV self-testing (HIVST) among men who have sex with men (MSM) in Pakistan: Seeing through the Consolidated Framework for Implementation Research (CFIR) lens
Source: PLOS Glob Public Health. 2026 Sep 11;6(9):e0005994. doi: 10.1371/journal.pgph.0005994 (PMC13567783; doi:10.1371/journal.pgph.0005994)
Supplement: Supplementary file 6 [file pgph.0005994.s007.docx]

The Editor,

PLOS Global Public Health,

22 June 2026

Dear Editor,

Thank you for sharing the reviewers' comments for the manuscript. I am making a point-by-point response to the concerns raised below.

**Reviwer 1:**

Need to clearly explain difficult to understand

**Rephrased the abstract**

No coherence from previous sentence to this sentence

Rephrased

You need to come up with some solution in the last line of the abstract; however, you come with barriers. You can move this sentence aboven and write potential recommendation of this study.

Rephrased the abstract

Key emergent themes highlighted that digital dispensation reduces HIV testing barriers and allows peer diffusion of testing services. MSMs can order HIVST digitally to dispense at the place of their choice. This allows them to bypass perceived stigma faced during in-person testing, including in the CBOs. However, MSM still fear stigma at home where HIVST might potentially be delivered. Participants noted difficulty in navigating the digital platform and a lack of comprehensive services inlcuding, including condoms, lubricants, and counselling. The participants emphasized the importance of demand generation through social media and dating applications. Provision of a comprehensive service delivery package, digital demand generation through social media, and dating applications is recommended for strengthening digital HIVST services.

MSM, HIVST can also be keywords for this study

Edited

Use refences before the full stop.

Edited

What is the key argument point of this study? why this study needed?

Stated following

Leveraging the potential of digital dispensation and HIVST. A digital HIV prevention and HIVST dispensation program is piloted in two major cities of Pakistan: Lahore and Karachi. The intervention was rolled out to improve testing coverage for MSM in the country . However, the intervention has not been rigorously evaluated. In the current study, we aim to by MSM community in Pakistan and identify gaps with recommendations for intervention improvement.

This ethics statment does not refelect how you ensure the participants ethics, please explain in deatils e,g. do you receive consent from the participants? if yes how? write it here.

Mentioned now

Explain the study settings above the study participants

Stated setting before participants, also the digital dispensation description sets the background for intervention which is mentioned first thing in the methods section

The study was conducted at two CBOs, Dostana (Lahore) and Humraz (Karachi). People above 18 years of age, male, identified as men who have sex with men, who have received both in-person and digital HIV testing services, were identified and recruited by the outreach workers of two CBOs. CBO workers and those who had been diagnosed with HIV were excluded from the study. Purposive sampling was conducted.

This is a qualitative study then why you use exclusion criteria and you mention you use purposive sampling write it based on what criteria you purposively select the participants.

The reasons of excluding HIV positive people is because they are ineligible for HIVST servcies as they are already diagnosed, we also excluded CBO workers because they have high health literacy and we aimed to capture the experiences of common MSM. Mentioned now in methods

Section now reads a below.

CBO workers were excluded even if they were MSM, as they have high health literacy and are providers of the same service that was evaluated, while people already living with HIV do not require testing services. Purposive sampling was conducted.

What is your eligibility criteria? is this quantitive study? or qualitative study? in qualitative eligibility criteria terms are not familiar with? before revise this manuscript please read few more articile in qualitative nature related to HIVST

It is qualitative study as menitioned, eligibility for interviews was set as reasons mentioned above

Write it ethics section

Done

Researcher, background, years of experience, and language you apply explain it

Given in reflexivity statement which reads as follow:

The lead investigator has been working in HIV for nearly seven years, whereas XX has been working for nearly two decades. YY has been involved in community research and various qualitative projects, whereas XX has worked at the national level in HIV response with various public health agencies. The lead investigator identifies as a gay man and is a community health rights advocate, a psychiatrist, and a public health professional. The lead investigator advocates for readily available services free of stigma and discrimination for the MSM community. YY has worked in public health for over two decades, providing an overview of the data coding and analysis processes.

How you reach them?

As mentioned above with the help of outreach workers of organization

How you build rapport with the respondent?

The lead PI is a psychiatrist and trained in engaging with people. He used ice breaking questions of variable to nature to ensure comfort and develop rapport.

What kind of personal device?

An android workphone was used

**How you ensure the data saturation?**

How many IDIs and FGDs are you conducted for this study?

This is mentioned explicitly in results section, 11 IDI and 2 FGDs

What was you purpose for IDIs and What was your purpose for FGDs?

There was no separate objectives for IDI and FGD. The reason for chosing FGD was to ensure MSMs feel empowered and are able to communicate themselves freely

How you ensure the no new data are emerged?

It is out of the hands of researcher to ensure that no new data emerged

What is this? explain it clearly in detail

Explain how many coder coded the data? write it clearly

Mentioned

The data transcript was available to two independent researchers. After becoming familiar with the data, the researcher independently conducted description- and interpretation-focused coding. In the description-focused coding technique, the researcher assigns a code that captures the participant's experience without inference. In interpretation-focused coding, the researcher assigns codes to capture the underlying processes of the experience. Any discrepancy in coding was settled by mutual consensus and deliberation guided by a third researcher. After coding had been completed, codes were sorted into clusters using an individual-based sorting strategy. Codes were sorted into clusters based on similarities and differences.

This may not need to mention here. You can a supplementary table where all the theme, sub-theme and code can be show

What kinds of researcher role are applying though out the data collection, data management, team management, respondent selection and data analysis. Is there any potential biases to conduct this study?

The researchers have been transparent about their experience, identity with community and the advocacy, however, the PI was supervised by professor of public health well versed in data analysis to ensure reflective purpose and that bracketing of personal opinions

The lead investigator has been working in HIV for nearly seven years, whereas XX has been working for nearly two decades. YY has been involved in community research and various qualitative projects, whereas XX has worked at the national level in HIV response with various public health agencies. The lead investigator identifies as a gay man and is a community health rights advocate, a psychiatrist, and a public health professional. The lead investigator advocates for readily available services free of stigma and discrimination for the MSM community. YY has worked in public health for over two decades, providing an overview of the data coding and analysis processes.

**Why those (marginalised) repondent give them interview? how tresearcher manage them to do it?**

This is the part of data analysis

Included in data analysis mainheading

You do not conduct IDIs with any participants who received HIVST through online? If not how you get their experience of implementation related facilitators and barriers?

We have, however, HIVST as mentioned was also provided in drop-in-center, so participants first got introduced to HIVST in drop-in-center and got their first HIVST test there (index test) and later ordered online as they developed trust.

Whwre is intervention caharcteristics? and process? as per CFIR domain?

In CFIR 2.0 intervention characteristics is replaced by the word innovation, please see: <https://cfirguide.org/constructs>

**How it favoured postively? interpretation are very surface level**

What kind of user friendly guidance?

Video guidance as mentioned in the description of digital dispensation section:

To generate demand for HIV services, advertisements are made in CBO drop-in centers (DIC) as well as messaging on WhatsApp, which includes a package of two videos, one focusing on how to perform HIVST and the other on how to use digital platforms. The demand generation faces significant challenges, as CBOs have to work in relative secrecy given the legal and social atmosphere of the country.

**What kind of benefits they gets from the digital dispensation write it**

We limit ourselves to the data and highlight recurring themes. The benefit such as evasion of stigma, time and cost efficiency are highlighted in the themes

**The verbatim you provide are not link with the Subtheme title!!!**

**We believe the verbatim captures subtheme, we have clarified by added clarity in the verbatim in brackets. Here the person refers to the fact that home HIVST is preferrred as your are alone, while if you do in drop-in-center people are noticing you and judge your results by facial expression. Since, HIVST dispensation at home bypasses this, it allows more control on disclosure**

**The verbatim now reads as below**

*Because at home (where I get HIVST dispensed), no one else is around in that moment. But if you're taking the test here at the center (CBO), your facial expressions and how you react can give something away. If you get tense or scared (after getting the test), people around you (in the drop-in center) who are watching might notice. They’ll realize you're doing an HIV self-test and might assume the result is bad. MSM7, 23 years old, part-time employee, 14 years of education* **The verbatim does not reflect with the text**

**We have clarified the verbatim for understanding**

**It reads as**

Participant reported that through digital HIVST dispensation, they have more autonomy in terms of confidentiality.

*When we come here, we have to first go to the office and give our identity. Then we visit the doctor ot the paramedic, and he conducts the test. But at home, we do it ourselves. We already know how. In Punjabi, we say “don’t open your mouth… meaning, just do it quietly and easily.’’FGD1*

**HIVST reduces cost or digital HIVST reduces the cost?**

**Digital HIVST; ammended**

**Same findings dicussed earlier, seems repetation**

**Here the theme refers to cost barriers not barriers in general, so it is different**

**You taling about stigma several times but not specify the what kind of stigma you found for the HIVST use and how digital platform help to reduce it?**

**This line captures the point**

Stigma from testing in CBOs is replaced by stigma in testing at home.

**Please explain what kind of fear you found?**

**In Muslim contexts or in Pakistan if your family gets to know you are MSM, family may discriminate or subject them to physical violence, the fear refers to that**

**Clarified as follow**

*That fear is always there (the fear of people knowing that you are MSM)… if people get to know, the reputation you have made as a straight man (by being in the closet) is tarnished. Your family prestige is at stake (as MSM is seen as a sin, abomination),. MSM8, 47 years old, artist by profession, 16 years of education*

**Why participants experice lack of privacy at home? what make them concern?**

**Because Pakistan is a collectivist society**

**The practices are highlight in the following verbatim**

if someone in the family even has the slightest doubt, they’ll open it. Either that, or your reputation at home needs to be so solid that your privacy is respected.

**What kind of demenqad they have please explain clearly**

**It refers to demand generation of services for including more and more MSM unreached by camapigns on grindr etc**

**The findings explain all domains of CFIR? How?**

**Mapped on CFIR domain as given in Table III**

**Discuss why people accept HIVST or not?**

**Discussing HIVST will be tangetial, we have added following line to document reason for aceptance of digital HIVST interventions**

The participants in our sample emphasize the convenience and resource efficiency of digital dispensation of HIVST, stating relative advantage over facility-based HIV testing

**Discuss how dogital HIVST dispention, stigma and mental health support work together and its relevance?**

**The following paragraph explain the concept**

MSMs are marginalized in many muslim-majority countries, with Pakistan being one of them[3]. This poses unique challenges in reaching out to the community with health and HIV interventions. Stigma is faced in family settings, legal frameworks, as well as internalized or perceived stigma within community settings[23]. In the current study, participants have described how stigma, even within community settings such as CBOs, serves as a barrier to HIV testing uptake. HIVST through digital means provides an additional testing option for the MSM community in that context. However, the stigma in CBO testing is replaced by fear of stigma in home settings, an interaction of inner and outer settings. Thus, digital dispensation of HIVST is influenced by stigma but in different contexts. The fear of enacted stigma is not unfounded, as MSM and people living with HIV (PLHIV) are highly stigmatized in the context of Pakistan. In the National HIV Stigma Index 2.0, 10% of MSM living with HIV reported that they were excluded from family settings in the last twelve months because of their identity as MSM[5]. Around 21.53% of MSM PLHIV also reported that they felt they were gossiped about and discriminated against by family in the last year because they are MSM. The stigma is not limited to same sex prefrences or behaviors. PLHIV are also excluded and discriminated against in family settings[5]. Around 5% of PLHIV report that they are excluded from family events in the last twelve months. Due to this stigma, people refrain from sharing their HIV status with their family[5]. In the National Stigma Index 2.0, 26.6% of MSM PLHIV reported they feel uncomfortable sharing their HIV status with those close to them due to stigma. Testing in such a stigmatizing environment thus brings anxiety, as reported by the participants[5]. In a meta-synthesis of HIVST interventions among MSM in Asia, it was found that mental distress is in fact a barrier for HIVST uptake[10]. The participants from our study have, in fact, stressed the need for counselling services, though provided digitally for users of digital dispensation.

**You can refer your experience related study findings of apps or digital platform**

**Unable to understand the point but we cannot share our experience**

**What is the take home message please clear it?**

**What is your core argument answere and how this research can make a policy action? who can be involved? who will be benefited from this study?**

**These two sections are ammended and policy implication section is added**

**Policy implications**

There are several policy implications from our study. First, there are some navigation difficulties for the MSM on the digital dispensation platform. Secondly, there are two platforms, one for HIV information and the second for ordering HIVST. These platforms should be simplified, and only one comprehensive platform serving both these purposes should be rolled out. Furthermore, comprehensive HIV services should be provided. This includes HIVST but also STI medications, PrEP, and condom dispensation digitally.

Rolling out these services by digital platform shall require review of current regulations of dispensation of medicinal products digitally in the country. It should also require liaison with law enforcement agencies, as there have been reports of people harassed for carrying condoms[30]. Thus, if digital dispensation of condoms is made, there is a need for engagement with critical enablers such as medical regulators and law enforcement agencies.

Increasingly, MSM have moved from conventional hotspots to digital dating spaces; however, bans on these applications make it impossible for national HIV programs to introduce digital demand generation. HIV advocates should lobby with the Government of Pakistan to unban these applications and launch a digital demand generation campaign for effective reach to unreached MSM.

**Conclusion**

Digital HIVST dispensation is acceptable. There is a community readiness to uptake a comprehensive package of HIV digitally, in addition to HIVST. The intervention also holds promise of network expansion of services driven by peers to MSM not reached by the CBOs. However, participants noted difficulty in platform navigation. Thus, the current digital dispensation platform should be improved and should include comprehensive HIV services such as dispensation of condoms and STI medications.

There is a need of digital demand generation and interpersonal interaction at demand generation as well as the testing stage in the form of video counselling. The current banning of MSM dating sites poses a significant barrier to demand generation. Policymakers should advocate for destigmatizing HIV testing and unbanning MSM dating websites so that HIVST demand generation could be fostered through these applications.

**Reviewer 3:**

**1. There are few stigma citations for the stigma faced among MSM in the introduction section.**

**I am unsure what this refers to
2. In the introduction section, UN's goal 90–90-90 was stated but only the first 90 was explained. Emphasize the importance of not just testing but continuum of care.**

**We believe explaining second two 90s would be tangential as it is out of scope of the paper
3. The method section did not specify clearly if the inclusion and exclusion criteria were the same for both IDIs and FGDS.**

**Interviews and the data collection process**

Once eligible participants were recruited, they were given the option of being interviewed either individually (in-depth interview) or in a focus group discussion (FGD).

Since this comes after eligibility, we imply that the inclusion and exclusion criteria is the same

Added this line to clarify further

The same eligibility criteria and interview guide were used for the in-depth interviews and FGDs.  **4. Did the FGDs and IDIs use the same interview guides? This was not clarified in the methods section and the reasons for using both IDIs and FGDs methods.**

The same eligibility criteria and interview guide were used for the in-depth interviews and FGDs.

**5. The analysis and findings section lacks clarity on stigma experiences and motivation for HIVST. Though enacted stigma has been mentioned in the manuscripts some selected participants' responses suggest instances of perceived community stigma with regards to receiving HIVST kits in their community, interpersonal stigma among peers and health care worker/facility stigma when utilizing traditional HIV testing options.**

**Clarity to the verbatims is added by adding phrases in brackets in verbatims. The benefits of cost and time efficiency was mentioned though we did not use the word motivation, we believe it is weighing and realizing of the benefits than motivation.**

**6. Issues around confidentiality and linkage to care were not explicitly highlighted with the digital HIVST Dispensation intervention. Please clarify if participants were required to report their HIVST results? If they did, was it through the same digital platforms and how was confidentiality ensured? This will be imperative to highlight in the results and discussion section.**

Added following line in the **Description of the digital dispensation HIVST model, section**

Reporting of HIVST is not necessary.  **7. The findings from CFIR domains and constructs framework were not thoroughly discussed with implication on the intervention in the discussion section.**

**We have made policy implication section to better address the point, we have used CFIR lens but engaged with diffusion of innovation theory as well to explain findings, thus not forcing data to fit in one framework**
