# Supplementary material for: Evaluating the digital dispensation of HIV self-testing (HIVST) among men who have sex with men (MSM) in Pakistan: Seeing through the Consolidated Framework for Implementation Research (CFIR) lens
Source: PLOS Glob Public Health. 2026 Sep 11;6(9):e0005994. doi: 10.1371/journal.pgph.0005994 (PMC13567783; doi:10.1371/journal.pgph.0005994)
Supplement: Peer review history

# Peer review: Decision Letter 0

**DOI:** 10.1371/journal.pgph.0005994.r001

**Authors:** Stephanie van Wyk

15 Jun 2026

PGPH-D-26-00385

Qualitative evaluation of the digital HIV self-testing (HIVST) intervention among men who have sex with men (MSM) in Pakistan.

PLOS Global Public Health

Dear Dr. Ali,

Thank you for submitting your manuscript to PLOS Global Public Health. After careful consideration, we feel that it has merit but does not fully meet PLOS Global Public Health’s publication criteria as it currently stands. Therefore, we invite you to submit a revised version of the manuscript that addresses the points raised during the review process.

Please submit your revised manuscript by Aug 14 2026 11:59PM. If you will need more time than this to complete your revisions, please reply to this message or contact the journal office at globalpubhealth@plos.org. When you're ready to submit your revision, log on to https://www.editorialmanager.com/pgph/ and select the 'Submissions Needing Revision' folder to locate your manuscript file.

Please include the following items when submitting your revised manuscript:

- A letter that responds to each point raised by the editor and reviewer(s). You should upload this letter as a separate file labeled 'Response to Reviewers'.
- A marked-up copy of your manuscript that highlights changes made to the original version. You should upload this as a separate file labeled 'Revised Manuscript with Track Changes'.
- An unmarked version of your revised paper without tracked changes. You should upload this as a separate file labeled 'Manuscript'.

Guidelines for resubmitting your figure files are available below the reviewer comments at the end of this letter.

As the corresponding author, your ORCID iD is verified in the submission system and will appear in the published article. PLOS supports the use of ORCID, and we encourage all coauthors to register for an ORCID iD and use it as well. Please encourage your coauthors to verify their ORCID iD within the submission system before final acceptance, as unverified ORCID iDs will not appear in the published article. *Only*  the individual author can complete the verification step; PLOS staff *cannot*  verify ORCID iDs on behalf of authors.

We look forward to receiving your revised manuscript.

Kind regards,

Stephanie van Wyk, Ph.D

Guest Editor

PLOS Global Public Health

Journal Requirements:

1. Please provide separate figure files in .tif or .eps format.

For more information about figure files please see our guidelines:  LINK

https://journals.plos.org/globalpublichealth/s/figures

https://journals.plos.org/globalpublichealth/s/figures#loc-file-requirements

2. The resolution of Figure 1 is very low and somewhat difficult to read. It is important that our Editors and Peer Reviewers are able to read all parts of a submission. Please replace these figures with higher resolution copies.

3. In the online submission form, you indicated that "Available on request".

All PLOS journals now require all data underlying the findings described in their manuscript to be freely available to other researchers, either

1. In a public repository,

2. Within the manuscript itself, or

3. Uploaded as supplementary information.

This policy applies to all data except where public deposition would breach compliance with the protocol approved by your research ethics board. If your data cannot be made publicly available for ethical or legal reasons (e.g., public availability would compromise patient privacy), please explain your reasons by return email and your exemption request will be escalated to the editor for approval. Your exemption request will be handled independently and will not hold up the peer review process, but will need to be resolved should your manuscript be accepted for publication. One of the Editorial team will then be in touch if there are any issues.

4. Some material included in your submission may be copyrighted. According to PLOS’s copyright policy, authors who use figures or other material (e.g., graphics, clipart, maps) from another author or copyright holder must demonstrate or obtain permission to publish this material under the Creative Commons Attribution 4.0 International (CC BY 4.0) License used by PLOS journals. Please closely review the details of PLOS’s copyright requirements here: PLOS Licenses and Copyright. If you need to request permissions from a copyright holder, you may use PLOS's Copyright Content Permission form.

Please respond directly to this email or email the journal office and provide any known details concerning your material's license terms and permissions required for reuse, even if you have not yet obtained copyright permissions or are unsure of your material's copyright compatibility.

Figure 1: Please confirm whether you drew the images / clip-art within the figure panels by hand. If you did not draw the images, please provide (a) a link to the source of the images or icons and their license / terms of use; or (b) written permission from the copyright holder to publish the images or icons under our CC-BY 4.0 license. Alternatively, you may replace the images with open source alternatives. See these open source resources you may use to replace images / clip-art:

- https://commons.wikimedia.org

- https://openclipart.org/

If the reviewer comments include a recommendation to cite specific previously published works, please review and evaluate these publications to determine whether they are relevant and should be cited. There is no requirement to cite these works unless the editor has indicated otherwise.

Additional Editor Comments (if provided):

[Note: HTML markup is below. Please do not edit.]

Reviewers' comments:

Reviewer's Responses to Questions

**Comments to the Author**

1. Does this manuscript meet PLOS Global Public Health’s publication criteria?

Reviewer #1: No

Reviewer #2: Partly

**********

2. Has the statistical analysis been performed appropriately and rigorously?

Reviewer #1: N/A

Reviewer #2: N/A

**********

3. Have the authors made all data underlying the findings in their manuscript fully available (please refer to the Data Availability Statement at the start of the manuscript PDF file)?

The PLOS Data policy

Reviewer #1: No

Reviewer #2: No

**********

4. Is the manuscript presented in an intelligible fashion and written in standard English?

Reviewer #1: No

Reviewer #2: No

**********

Reviewer #1: This manuscript requires substantial improvement in ethical, methodological, findings and interpretation and recommendation as well as conclusion. That is why it requires substantial improvement and my recommendation is rejected.

Reviewer #2: Summary

This study qualitatively assesses a digital HIV self-testing (HIVST) intervention among men who have sex with men (MSM) in Pakistan, focusing on user experiences with an online platform that allows ordering of OraQuick self-test kits for home delivery. Using 12 in-depth interviews and two focus group discussions, the authors found that digital HIVST is generally acceptable and preferred because it reduces stigma, enhances confidentiality, and offers convenience compared to facility-based testing. Participants also noted the importance of peer sharing and digital platforms in potentially increasing HIV testing uptake within the MSM community. However, they identified key barriers, including concerns about confidentiality at home, ongoing stigma, challenges in navigating the platform, and fragmentation between different digital services. Participants further emphasized the need for a more comprehensive service package, including condoms, lubricants, and online counseling, as well as improved demand generation through social media and dating apps. Overall, the study indicates that while digital HIVST holds great potential in this context, optimizing platform design, service integration, and delivery strategies is necessary for effective scale-up.

Major Comments

1. The study does not clearly demonstrate whether data saturation was achieved, which is essential for qualitative rigor.

2. Sampling bias is a significant limitation, as participants were recruited through community-based organizations and are likely more engaged with HIV services than the broader MSM population.

3. The sample size is small and lacks diversity (e.g., mostly educated, urban participants), limiting generalizability.

4. The methodological description lacks detail, particularly regarding coding strategy, development of themes, and how discrepancies between coders were resolved.

5. The analysis is largely descriptive, with limited interpretive depth or engagement with relevant theoretical frameworks (e.g., stigma, digital health adoption).

6. There is conceptual overlap between themes (e.g., stigma, confidentiality, and acceptability), which weakens the clarity and structure of the findings.

7. The manuscript does not sufficiently explore contradictory perspectives (e.g., home testing as both private and potentially risky).

8. The discussion section lacks depth, particularly in translating findings into actionable policy or programmatic implications within the Pakistani context.

9. Ethical considerations are underdeveloped, given the criminalization and stigmatization of MSM, especially in relation to digital privacy and potential risks of home delivery.

10. The data availability statement (“available on request”) may not meet journal requirements and needs clarification or revision.

Minor Comments

1. Several grammatical and language issues are present; the manuscript would benefit from professional editing for clarity and flow.

2. There is repetition of participant quotes and ideas, particularly around stigma and confidentiality.

3. Table II contains an error (“mean age… 24,500 PKR”), indicating incorrect units and poor formatting.

4. Some references are incorrectly formatted or incomplete, and links (e.g., UNAIDS citation) appear inaccurate.

5. The ethical statement is repeated, suggesting duplication that should be corrected.

6. Terminology such as MSM, MSW, and bisexual is noted as confusing by participants, but not clearly defined in the manuscript.

7. The relationship between the two digital platforms (Sehat Dost and QuickRes) is not clearly explained and may confuse readers.

8. Transitions between sections and themes are abrupt, affecting overall readability.

9. The manuscript would benefit from clearer subheadings in the discussion to improve structure.

10. Some sentences are awkwardly phrased or unclear, requiring refinement for precision and readability.

**********

what does this mean?). If published, this will include your full peer review and any attached files.

**Do you want your identity to be public for this peer review?**  If you choose “no”, your identity will remain anonymous but your review may still be made public.

For information about this choice, including consent withdrawal, please see our Privacy Policy

Reviewer #1: **Yes:** Md. Alamgir Hossain

Reviewer #2: No

**********

[NOTE: If reviewer comments were submitted as an attachment file, they will be attached to this email and accessible via the submission site. Please log into your account, locate the manuscript record, and check for the action link "View Attachments". If this link does not appear, there are no attachment files.]

Attachment Submitted filename: PGPH-D-26-00385.pdf

Attachment Submitted filename: PGPH reviwer comments.pdf

# Author comment: Author response to Decision Letter 1

**DOI:** 10.1371/journal.pgph.0005994.r002

1 Jul 2026

Attachment Submitted filename: Response to editor.docx

# Editor report: Decision Letter 1

**DOI:** 10.1371/journal.pgph.0005994.r003

**Authors:** Stephanie van Wyk

9 Jul 2026

PGPH-D-26-00385R1

Qualitative evaluation of the digital HIV self-testing (HIVST) intervention among men who have sex with men (MSM) in Pakistan.

PLOS Global Public Health

Dear Dr. Ali,

Thank you for submitting your manuscript to PLOS Global Public Health. After careful consideration, we feel that it has merit but does not fully meet PLOS Global Public Health’s publication criteria as it currently stands. Therefore, we invite you to submit a revised version of the manuscript that addresses the points raised during the review process. Please address the comments made by Reviewer one indicated in the PDF copy.

Please submit your revised manuscript by Sep 07 2026 11:59PM. If you will need more time than this to complete your revisions, please reply to this message or contact the journal office at globalpubhealth@plos.org. When you're ready to submit your revision, log on to https://www.editorialmanager.com/pgph/ and select the 'Submissions Needing Revision' folder to locate your manuscript file.

Please include the following items when submitting your revised manuscript:

- A letter that responds to each point raised by the editor and reviewer(s). You should upload this letter as a separate file labeled 'Response to Reviewers'.
- A marked-up copy of your manuscript that highlights changes made to the original version. You should upload this as a separate file labeled 'Revised Manuscript with Track Changes'.
- An unmarked version of your revised paper without tracked changes. You should upload this as a separate file labeled 'Manuscript'.

Guidelines for resubmitting your figure files are available below the reviewer comments at the end of this letter.

As the corresponding author, your ORCID iD is verified in the submission system and will appear in the published article. PLOS supports the use of ORCID, and we encourage all coauthors to register for an ORCID iD and use it as well. Please encourage your coauthors to verify their ORCID iD within the submission system before final acceptance, as unverified ORCID iDs will not appear in the published article. *Only*  the individual author can complete the verification step; PLOS staff *cannot*  verify ORCID iDs on behalf of authors.

We look forward to receiving your revised manuscript.

Kind regards,

Stephanie van Wyk, Ph.D

Guest Editor

PLOS Global Public Health

Journal Requirements:

If the reviewer comments include a recommendation to cite specific previously published works, please review and evaluate these publications to determine whether they are relevant and should be cited. There is no requirement to cite these works unless the editor has indicated otherwise.

Additional Editor Comments (if provided):

[Note: HTML markup is below. Please do not edit.]

Reviewers' comments:

[NOTE: If reviewer comments were submitted as an attachment file, they will be attached to this email and accessible via the submission site. Please log into your account, locate the manuscript record, and check for the action link "View Attachments". If this link does not appear, there are no attachment files.]

# Author comment: Author response to Decision Letter 2

**DOI:** 10.1371/journal.pgph.0005994.r004

9 Jul 2026

Attachment Submitted filename: Response_to_editor_auresp_2.docx

# Peer review: Decision Letter 2

**DOI:** 10.1371/journal.pgph.0005994.r005

**Authors:** Stephanie van Wyk

4 Aug 2026

PGPH-D-26-00385R2

Qualitative evaluation of the digital HIV self-testing (HIVST) intervention among men who have sex with men (MSM) in Pakistan.

PLOS Global Public Health

Dear Dr. Ali,

Thank you for submitting your manuscript to PLOS Global Public Health. After careful consideration, we feel that it has merit but does not fully meet PLOS Global Public Health’s publication criteria as it currently stands. Therefore, we invite you to submit a revised version of the manuscript that addresses the points raised during the review process.

Please submit your revised manuscript by Oct 03 2026 11:59PM. If you will need more time than this to complete your revisions, please reply to this message or contact the journal office at globalpubhealth@plos.org. When you're ready to submit your revision, log on to https://www.editorialmanager.com/pgph/ and select the 'Submissions Needing Revision' folder to locate your manuscript file.

Please include the following items when submitting your revised manuscript:

- A letter that responds to each point raised by the editor and reviewer(s). You should upload this letter as a separate file labeled 'Response to Reviewers'.
- A marked-up copy of your manuscript that highlights changes made to the original version. You should upload this as a separate file labeled 'Revised Manuscript with Track Changes'.
- An unmarked version of your revised paper without tracked changes. You should upload this as a separate file labeled 'Manuscript'.

Guidelines for resubmitting your figure files are available below the reviewer comments at the end of this letter.

As the corresponding author, your ORCID iD is verified in the submission system and will appear in the published article. PLOS supports the use of ORCID, and we encourage all coauthors to register for an ORCID iD and use it as well. Please encourage your coauthors to verify their ORCID iD within the submission system before final acceptance, as unverified ORCID iDs will not appear in the published article. *Only*  the individual author can complete the verification step; PLOS staff *cannot*  verify ORCID iDs on behalf of authors.

We look forward to receiving your revised manuscript.

Kind regards,

Stephanie van Wyk, Ph.D

Guest Editor

PLOS Global Public Health

Journal Requirements:

If the reviewer comments include a recommendation to cite specific previously published works, please review and evaluate these publications to determine whether they are relevant and should be cited. There is no requirement to cite these works unless the editor has indicated otherwise.

Additional Editor Comments (if provided):

[Note: HTML markup is below. Please do not edit.]

Reviewers' comments:

Reviewer's Responses to Questions

**Comments to the Author**

Reviewer #1: All comments have been addressed

Reviewer #3: All comments have been addressed

**********

publication criteria?

Reviewer #1: No

Reviewer #3: Partly

**********

3. Has the statistical analysis been performed appropriately and rigorously?

Reviewer #1: No

Reviewer #3: Yes

**********

4. Have the authors made all data underlying the findings in their manuscript fully available (please refer to the Data Availability Statement at the start of the manuscript PDF file)?

The PLOS Data policy

Reviewer #1: Yes

Reviewer #3: Yes

**********

5. Is the manuscript presented in an intelligible fashion and written in standard English?

Reviewer #1: No

Reviewer #3: Yes

**********

Reviewer #1: This manuscript has lots of scientific gap that requires to answer

Reviewer #3: The study qualitatively assessed HIVST dispensation and demand creation intervention among MSM in Pakistan using the CFIR model. The findings highlighted the process of navigating these platforms and the barriers encountered with the use of the digital dispensation platforms and demand creation activities. Criminalization of same-sex activities, stigma, cost and privacy were listed as barriers with use of HIVST in Pakistan among the participants. Overall, the study highlighted the preference for HIVST and the use of digital dispensation of test kits to the traditional testing at CBOs. The authors responded adequately to some of the comments by the early reviewers. Below are some comments for consideration for acceptance of the manuscript upon review.

1. There are few stigma citations for the stigma faced among MSM in the introduction section.

2. In the introduction section, UN's goal 90–90-90 was stated but only the first 90 was explained. Emphasize the importance of not just testing but continuum of care.

3. The method section did not specify clearly if the inclusion and exclusion criteria were the same for both IDIs and FGDS.

4. Did the FGDs and IDIs use the same interview guides? This was not clarified in the methods section and the reasons for using both IDIs and FGDs methods.

5. The analysis and findings section lacks clarity on stigma experiences and motivation for HIVST. Though enacted stigma has been mentioned in the manuscripts some selected participants' responses suggest instances of perceived community stigma with regards to receiving HIVST kits in their community, interpersonal stigma among peers and health care worker/facility stigma when utilizing traditional HIV testing options.

6. Issues around confidentiality and linkage to care were not explicitly highlighted with the digital HIVST Dispensation intervention. Please clarify if participants were required to report their HIVST results? If they did, was it through the same digital platforms and how was confidentiality ensured? This will be imperative to highlight in the results and discussion section.

7. The findings from CFIR domains and constructs framework were not thoroughly discussed with implication on the intervention in the discussion section.

**********

what does this mean?). If published, this will include your full peer review and any attached files.

**Do you want your identity to be public for this peer review?**  If you choose “no”, your identity will remain anonymous but your review may still be made public.

For information about this choice, including consent withdrawal, please see our Privacy Policy

Reviewer #1: **Yes:** Md. Alamgir Hossain

Reviewer #3: No

**********

[NOTE: If reviewer comments were submitted as an attachment file, they will be attached to this email and accessible via the submission site. Please log into your account, locate the manuscript record, and check for the action link "View Attachments". If this link does not appear, there are no attachment files.]

Attachment Submitted filename: PGPH-D-26-00385_R2_reviewer-Alm.pdf

# Author comment: Author response to Decision Letter 3

**DOI:** 10.1371/journal.pgph.0005994.r006

4 Aug 2026

Attachment Submitted filename: Comments from reviewers.docx

# Editor report: Decision Letter 3

**DOI:** 10.1371/journal.pgph.0005994.r007

**Authors:** Stephanie van Wyk

12 Aug 2026

Qualitative evaluation of the digital HIV self-testing (HIVST) intervention among men who have sex with men (MSM) in Pakistan.

PGPH-D-26-00385R3

Dear Dr Ali,

We are pleased to inform you that your manuscript 'Qualitative evaluation of the digital HIV self-testing (HIVST) intervention among men who have sex with men (MSM) in Pakistan.' has been provisionally accepted for publication in PLOS Global Public Health.

Before your manuscript can be formally accepted you will need to complete some formatting changes, which you will receive in a follow up email. A member of our team will be in touch with a set of requests.

Please note that your manuscript will not be scheduled for publication until you have made the required changes, so a swift response is appreciated.

IMPORTANT: The editorial review process is now complete. PLOS will only permit corrections to spelling, formatting or significant scientific errors from this point onwards. Requests for major changes, or any which affect the scientific understanding of your work, will cause delays to the publication date of your manuscript.

If your institution or institutions have a press office, please notify them about your upcoming paper to help maximize its impact. If they'll be preparing press materials, please inform our press team as soon as possible -- no later than 48 hours after receiving the formal acceptance. Your manuscript will remain under strict press embargo until 2 pm Eastern Time on the date of publication. For more information, please contact globalpubhealth@plos.org.

Thank you again for supporting Open Access publishing; we are looking forward to publishing your work in PLOS Global Public Health.

Best regards,

Stephanie van Wyk, Ph.D

Guest Editor

PLOS Global Public Health

***********************************************************

Reviewer Comments (if any, and for reference):
